# Spontaneous Cyclic Levitation of Glycerol‐Water Droplets at Low Pressures

**DOI:** 10.1002/advs.77772

**Published:** 2026-09-13

**Authors:** Chen Xu, Renjie Hua, Wei Ma, Fuqiang Chu, Dong Liang, Xueshi Li, Qirui Zhang, Yuan Dong, Xuehu Ma, Xiao Yan, Shuhuai Yao

**Affiliations:** ^1^ Department of Mechanical and Aerospace Engineering The Hong Kong University of Science and Technology Hong Kong China; ^2^ School of Mechanical Engineering Hangzhou Dianzi University Hangzhou China; ^3^ School of Energy and Environmental Engineering University of Science and Technology Beijing Beijing China; ^4^ Sichuan University‐Pittsburgh Institute Chengdu China; ^5^ Future Intelligent Wear Centre, School of Fashion and Textiles The Hong Kong Polytechnic University Hong Kong China; ^6^ State Key Laboratory of Fine Chemicals, Frontier Science Center For Smart Materials, Liaoning Key Laboratory of Clean Utilization of Chemical Resources, School of Chemical Engineering Dalian University of Technology Dalian China; ^7^ Key Laboratory of Low‐Grade Energy Utilization Technologies and Systems Chongqing University, Ministry of Education Chongqing China; ^8^ Institute of Engineering Thermophysics, School of Energy and Power Engineering Chongqing University Chongqing China

**Keywords:** droplet dynamics, freezing, low pressure, vaporization

## Abstract

At low pressures, water often experiences chaotic multiphase transitions that complicate fluid management. Here, we demonstrate that a minimal addition of glycerol to a water droplet can transform these disruptive behaviors into highly regulated dynamics. Instead of typical breakup or rapid self‐propulsion, freezing glycerol‐water droplets enter a state of gentle, cyclic levitation, repeatedly undergoing “dwelling, liftoff, flight, and impact.” We reveal that this phenomenon arises from the dual roles of glycerol. Freeze‐concentration preserves interconnected liquid regions and prevents the formation of a closed outer ice shell, thereby limiting the accumulation of expansion‐induced stress. In parallel, glycerol lowers the equilibrium freezing temperature, reducing the asymmetric vaporization recoil that drives self‐propulsion during recalescence. The suppression of these instabilities enables a cyclic droplet motion driven by self‐sustaining thermal oscillation, where basal heating enhances overpressure for liftoff and in‐flight vaporization cooling resets the cycle. Our findings unveil a distinct regime of vaporization‐driven droplet dynamics that is tunable through composition, offering new strategies for fluid management under extreme conditions, with implications ranging from thermal management to space exploration.

## Graphical abstract

Under low pressure, a minute glycerol addition transforms the chaotic dynamics of freezing water droplets into cyclic levitation. Freeze‐concentration preserves liquid pathways that relax expansion‐induced stress, while freezing point depression weakens asymmetric vaporization recoil. With fracturing and self‐propulsion suppressed, repeated dwelling, liftoff, flight, and impact arise from self‐sustained thermal oscillations between substrate heating and in‐flight vaporization cooling.

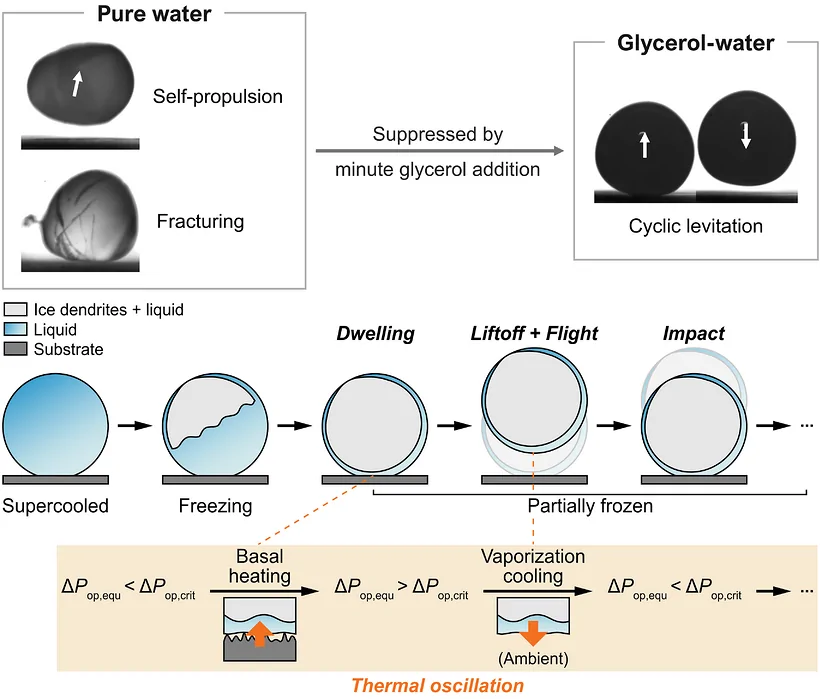

## 1 Introduction

Phase transitions of water are critical in fluid management, with applications ranging from biological preservation [1] and materials processing [2, 3, 4] to thermal management [5] and extraterrestrial resource utilization [6]. Unlike atmospheric phase changes, which are typically constrained by high saturation temperatures and heat transfer limits, low‐pressure environments allow water's internal sensible heat to drive intense vaporization [7]. This rapid removal of latent heat can induce deep supercooling and subsequent freezing, triggering chaotic instabilities [8, 9, 10, 11, 12, 13, 14]. Specifically, the anomalous volumetric expansion of freezing water generates immense internal stress within the ice shell, leading to brittle fracture and explosive breakup [8, 9]. In addition, the rapid release of latent heat during recalescence can induce localized vaporization, driving intense self‐propulsion [10, 11, 12, 13, 14, 15]. These phenomena not only highlight the intricate coupling of freezing and vaporization, but also present a fundamental challenge to the precise fluid control required in emerging engineering systems.

Here, we ask: Can this chaos be tamed? Introducing a solute has proven effective in modulating fluid behavior by tuning the system's fundamental thermo‐physical properties. For instance, the dynamic redistribution of solutes at interfaces can influence hydrodynamic outcomes such as droplet splashing [16], wetting [17], and particle transport [18]. Additionally, solutes can alter phase transitions through mechanisms such as colligative freezing point depression [19] and changes in ice crystal growth dynamics [20]. Yet, the coupled interplay between bulk and interfacial effects in governing multiphase dynamics remains largely unexplored, leaving a critical gap in our understanding of how solutes can mitigate the intense instabilities during freezing in low‐pressure environments.

In this study, we demonstrate that minute addition of glycerol to water fundamentally alters its freezing behavior under low‐pressure conditions. Glycerol suppresses two disruptive instabilities through distinct mechanisms: freeze‐concentration prevents the formation of a confining ice shell and preserves liquid channels that relax expansion‐induced stress, while the reduction in equilibrium freezing temperature decreases the asymmetric vaporization recoil that drives self‐propulsion during recalescence. This suppression reveals a metastable regime of cyclic levitation, where the droplet harvests thermal energy from the substrate to drive rhythmic, self‐sustaining flights. The resulting motion is fundamentally distinct from the continuous suspension of Leidenfrost solids [21] and the quasi‐elastic bouncing of deformable liquid droplets, such as droplets in the Leidenfrost state [22] and droplets undergoing low‐pressure trampolining [10]. Here, the partially frozen droplet exhibits discrete, rigid‐body motion cycles. In addition, cyclic levitation is driven by overpressure generated in the free molecular flow regime, necessitating a model different from the slip‐flow‐based model used to describe low‐pressure trampolining [10]. Through visualization and multiscale modeling, we show that this cyclic levitation is driven by the interplay between localized heating from the substrate during dwelling and vaporization cooling during flight. By mapping droplet freezing dynamics across various superhydrophobic substrates and glycerol concentrations, we establish that cyclic levitation reliably emerges at low glycerol fractions once the disruptive instabilities are suppressed. This work reveals a new regime of vaporization‐driven dynamics and introduces compositional tuning as a robust method for modulating complex phase‐change behaviors.

## 2 Results and Discussion

**FIGURE 1:**
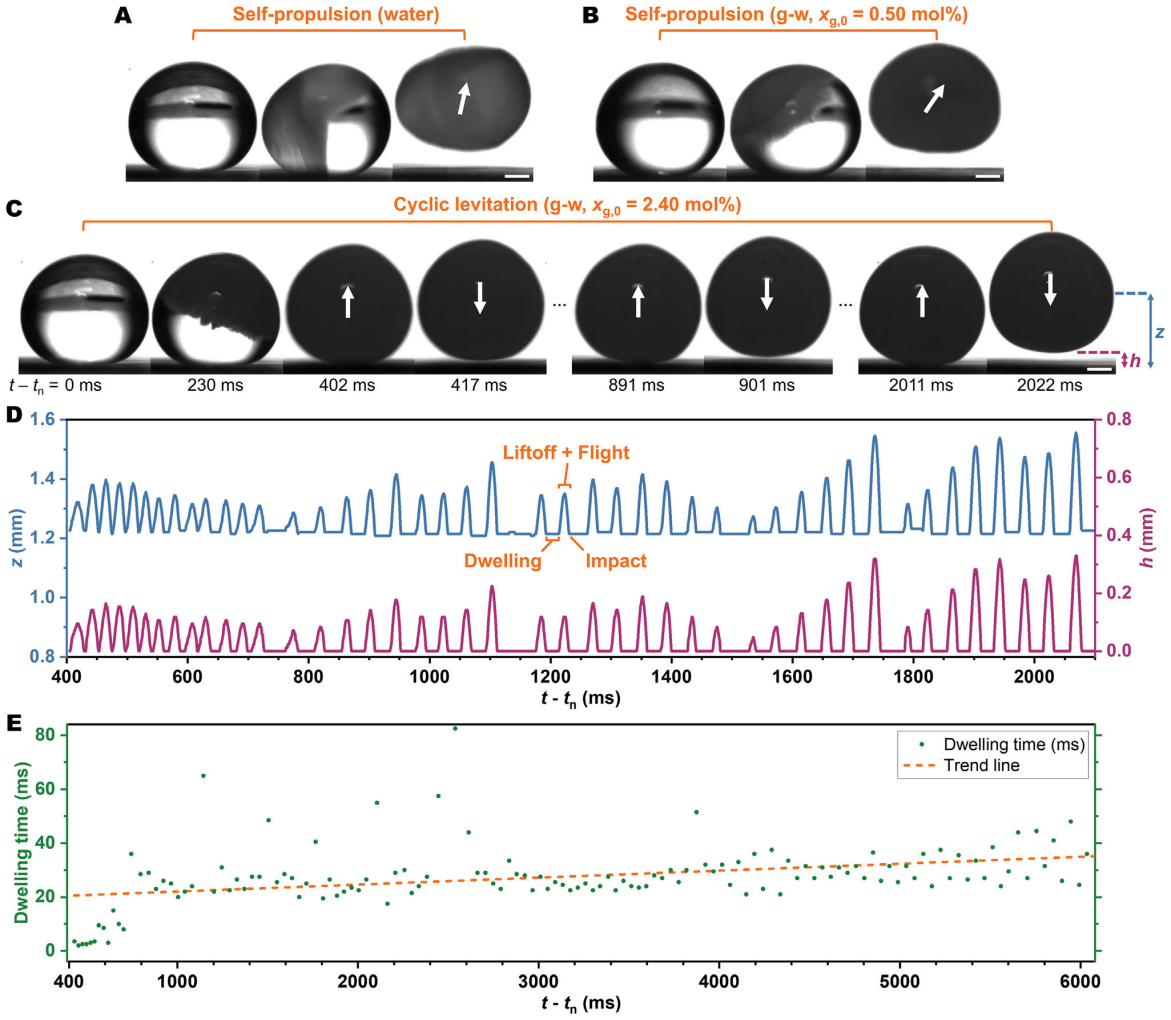
Droplet dynamics during freezing at low pressures. (A) Self‐propulsion of a freezing pure water droplet on Al‐0 substrate, a phenomenon consistent with previous reports [10, 11, 12, 13, 14]. The white arrow indicates the direction of self‐propulsion. (B) Similar self‐propulsion of a freezing glycerol‐water droplet with *x*_g,0_ = 0.50 mol% on the same substrate. (C) Cyclic levitation of a partially frozen glycerol‐water droplet with *x*_g,0_ = 2.40 mol% on the same substrate. White arrows indicate the directions of motion. Time *t* is shown relative to the moment of nucleation *t*_n_. (D) Temporal evolution of the droplet center height *z* from the contact line and visible vapor cushion thickness *h*, derived from the image sequence in panel (C). The synchronous change in *z* and *h* indicates rigid‐body motion, in contrast to the asynchronous behavior of Leidenfrost liquid droplets [22]. The levitations are characterized by cyclic “dwelling—liftoff—flight—impact” events (see also Movie S1). (E) Dwelling times derived from the image sequence in panel (C). Dwelling times (dots) are initially short and, in later cycles, remain on the order of 10 milliseconds while steadily increasing (dashed line). The scale bars in panels (A), (B), and (C) represent 0.5 mm. The uncertainty for *z* and *h* is ±0.012 mm (±1 pixel after spatial calibration); for dwelling time it is ±0.5 ms (±1 frame at 2000 fps). These values represent image‐analysis resolution limits rather than statistical variation across replicates.

We examined glycerol‐water droplets on superhydrophobic substrates under rapid depressurization (see Materials and Methods for experimental details). Starting from ambient conditions (295 ± 1 K, 101.3 kPa), a ∼10 µL droplet of the experimental fluid (glycerol‐water solutions with initial glycerol mole fraction *x*_g,0_ ranging from 0 to 4.69 mol%, where 0 mol% denotes pure water) was deposited onto a horizontal substrate inside a vacuum chamber. The chamber pressure was then rapidly reduced to below 100 Pa in ∼20 s (Figure S1C). This depressurization induced strong vaporization cooling, causing the droplet to supercool (Figure S1B) and minimizing water loss from vaporization. For a droplet with *x*_g,0_ of 2.40 mol%, the glycerol mole fraction was estimated to increase to 2.84 mol% prior to ice nucleation (Section S2). As shown in Figure 1A–C, the addition of glycerol fundamentally alters the droplet's vaporization‐induced dynamics. To elucidate this transformation, we first investigate how glycerol suppresses chaotic freezing instabilities (‘Suppression of initial freezing instabilities’ section), and then examine the core discovery of this work: the emergence of the cyclic levitation (‘Emergence of cyclic levitation’ section).

### 2.1 Suppression of Initial Freezing Instabilities

**FIGURE 2:**
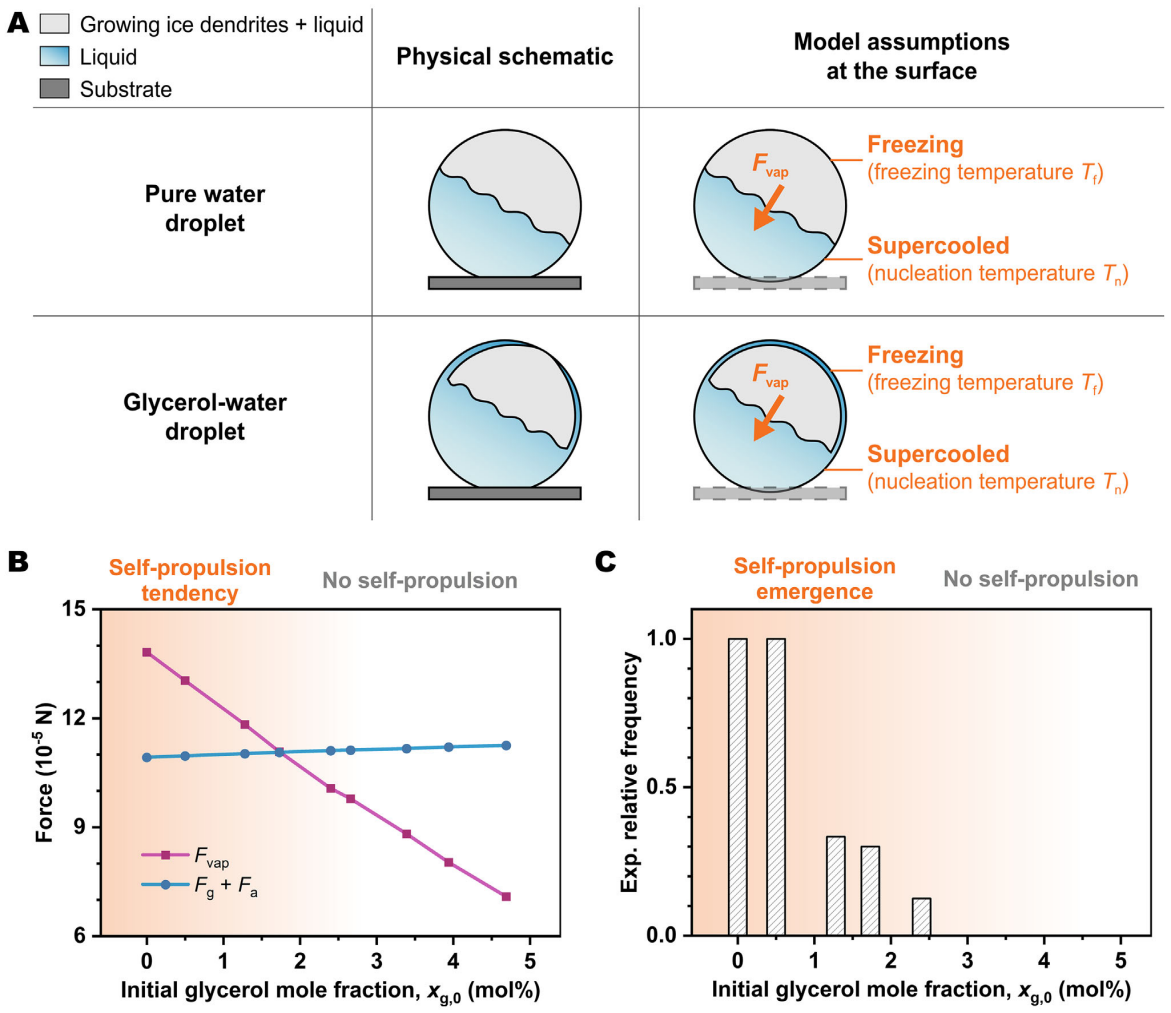
Suppression of self‐propulsion during recalescence. (A) Schematic of the simplified model for pure water and glycerol‐water droplets during recalescence. The left side depicts the physical scenario; the right side outlines temperature assumptions for the “freezing” and “supercooled” regions. The droplet is approximated as quasi‐spherical with constant geometry. Substrate contact is considered only for substrate adhesion *F*_a_. Consequently, the vaporization force *F*_vap_ acts from the freezing region to the supercooled region (orange arrow). (B) Modeled forces (vaporization force *F*_vap_, gravity and substrate adhesion *F*_g_ + *F*_a_) as a function of *x*_g,0_ for droplets of assumed fixed pre‐nucleation size (*R*_d_ = 1.26 mm, *R*_c_ = 0.419 mm). As *x*_g,0_ increases, *F*_vap_ decreases significantly, while *F*_g_ + *F*_a_ increases slightly. The background gradient indicates the progressive suppression of self‐propulsion predicted by the decreasing force ratio *F*_vap_/(*F*_g_ + *F*_a_). (C) Experimentally measured relative frequency of self‐propulsion on the Al‐0 substrate (from Figure 5). The background gradient indicates the decreasing trend in the emergence frequency of self‐propulsion with increasing *x*_g,0_.

Vaporization cooling induces spontaneous nucleation at the supercooled droplet's free surface [10, 12, 14, 23], followed by rapid crystallization that releases latent heat and causes a sharp temperature rise from the nucleation temperature, a process termed recalescence (Figure S1B; see also Section S9). Prior studies [10, 12, 13, 14, 15] reported that recalescence can produce self‐propulsion of pure water droplets (Figure 1A); here, we show that glycerol addition progressively suppresses this behavior by changing the droplet's thermodynamics. During recalescence, the droplet surface separates into distinct thermal regions [12, 13, 14, 15]: a “freezing” region governed by the equilibrium freezing temperature *T*_f_ and a “supercooled” region governed by the nucleation temperature *T*_n_ (Figure 2A). This thermal contrast generates an asymmetric vaporization recoil pressure *P*_r_ on the droplet surface, which can be estimated from the vaporization mass flux *J*_vap_: *P*_r_ ≈ *J*_vap_^2^/ρ_v_∝*P*_v_, where *ρ*_v_ is the local vapor density (see Section S6 for derivations). The net vaporization force *F*_vap_ propelling the droplet acts across its projected area from the freezing region towards the supercooled region: *F*_vap_ =  π*R*_d_^2^[*P*_r_(*T*_f_) − *P*_r_(*T*_n_)], where *R*_d_ is the equivalent droplet radius. For droplets of pure water or dilute glycerol‐water solutions (*x*_g,0_ = 0.5 mol%), *F*_vap_ significantly exceeds the combined resisting forces of gravity *F*_g_ and substrate adhesion *F*_a_, driving rapid self‐propulsion (Figure 1A,B and Movie S2). Remarkably, because the vapor pressure *P*_v_ rises exponentially with temperature, *P*_r_∝*P*_v_ is much larger in the warmer “freezing” region than in the “supercooled” region (Figure S4B). Increasing glycerol concentration therefore reduces *P*_r_ (and therefore *F*_vap_) through two coupled effects: it lowers *T*_f_ and decreases the water activity of the freeze‐concentrated liquid. Our model accounts for both effects, but it shows that the temperature effect is dominant over the composition effect within the tested concentration range (Figure S4B). Consequently, *F*_vap_ decreases relative to *F*_g_ + *F*_a_ (Figure 2B). Even though these forces may not be collinear, the ratio *F*_vap_/(*F*_g_ + *F*_a_) serves as an effective indicator of self‐propulsion propensity: its decrease with *x*_g,0_ agrees with the experimentally observed reduction in self‐propulsion frequency (Figure 2C). Here, substrate adhesion *F*_a_ is estimated using the Young‐Dupré relation and an ambient‐pressure receding contact angle; the limitations of this approximation are discussed in Section S7.

**FIGURE 3:**
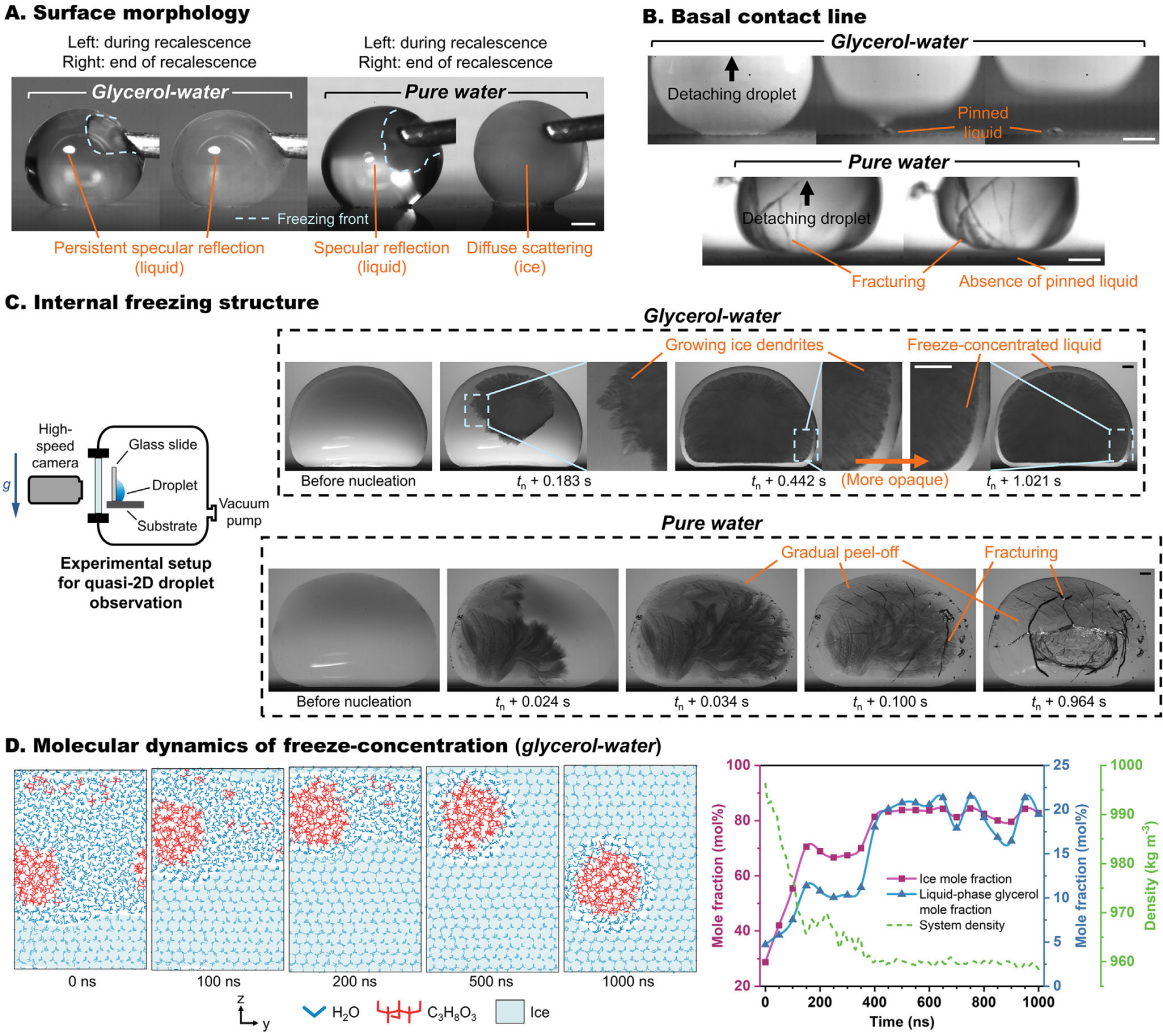
Composite freezing structure of glycerol‐water droplets. (A) Comparison of surface morphologies between a glycerol‐water droplet (*x*_g,0_ = 2.40 mol%) and a pure water droplet. The partially frozen glycerol‐water droplet maintains a smooth, liquid‐like surface (evidenced by the persistent specular reflection), in contrast to the rough, opaque surface of the pure water droplet (diffuse scattering due to surface ice). Identical lighting conditions were maintained for each liquid during its freezing process. To prevent self‐propulsion during recalescence, droplets were fixed on a thermocouple, where heterogeneous nucleation preferentially occurred. (B) Observation of the basal contact line during droplet detachment. Pinned liquid is visible for the partially frozen glycerol‐water droplet (*x*_g,0_ = 2.40 mol%), confirming the presence of peripheral liquid. In contrast, the detaching pure water droplet exhibits no pinned liquid and fractures significantly. (C) Quasi‐2D visualization of the internal freezing structure of a glycerol‐water droplet (*x*_g,0_ = 1.73 mol%) and a pure water droplet. A slightly lower *x*_g,0_ is used to enhance vaporization cooling in the experimental setup (far left, see ‘Quasi‐Two‐Dimensional (Quasi‐2D) Droplet’ in Materials and Methods for details). The high‐speed image sequence illustrates the freeze‐concentration process in the glycerol‐water droplet: following nucleation, the growth of the ice dendrites leads to the formation of a liquid layer around the core, while the stabilizing core becomes increasingly opaque, indicating ice dendrite growth within a concentrated liquid matrix. In contrast, the volumetric expansion of freezing water causes gradual peel‐off (from the front glass slide) and fracturing in the pure water droplet. Time is shown relative to the moment of nucleation *t*_n_. See Movies S3 and S4 for the corresponding videos. (D) Molecular dynamics (MD) simulation of a freezing glycerol‐water solution (∼3.3 mol%) at 250 K and 0 atm, where periodic boundary conditions are employed in the XYZ directions. Glycerol (C_3_H_8_O_3_) molecules are rejected from the growing ice lattice (shaded light blue) into the remaining liquid (See Section S5 for simulation details). After rapid crystallization during the first ∼400 ns, the finite simulated system approaches an ice mole fraction of ∼85 mol%, a liquid‐phase glycerol mole fraction of ∼20 mol%, and a density decrease of ∼4%. These values support the freeze‐concentration mechanism qualitatively and are not treated as macroscopic predictions. The scale bars in panels (A), (B), and (C) represent 0.5 mm.

Glycerol also suppresses a second chaotic instability: droplet fracturing. Pure water droplets that freeze from the outside‐in typically fracture (Figure 3B,C and Movies S3 and S4), because the ∼9% volumetric expansion of water upon freezing is confined by an ice shell, generating large internal stress [8, 9]. In contrast, glycerol‐water droplets remain intact at all concentrations tested (Figure 3B,C and Movies S2 and S3). This stability arises primarily from a freeze‐concentration process: advancing ice dendrites reject solute into the remaining liquid during the gradual solidification [24, 25, 26, 27]. Instead of forming a solid confining shell, the droplet solidifies into a composite structure of an ice “skeleton” permeated by interconnected, unfrozen liquid channels. The persistent peripheral liquid layer and internal liquid channels allow local deformation and stress relaxation while the reduced frozen fraction limits the net volumetric expansion. Experimentally, this composite freezing structure is evidenced by a persistent peripheral liquid layer (displaying smooth specular reflection, distinct from the opaque peripheral ice of pure water, Figure 3A), liquid pinned at the contact line upon detachment (Figure 3B), and internal morphology imaging (Figure 3C and Movie S3). Furthermore, freezing proceeds much more slowly in the glycerol‑containing droplets (∼10–20 times slower than pure water, estimated from Movie S2), facilitating the stress relaxation.

Molecular dynamics (MD) simulations (Figure 3D and Section S5) provide the microscopic mechanism underlying the freeze‐concentration process: glycerol is dynamically rejected by the advancing ice, creating a solute‐rich liquid buffer ahead of the crystallization interface. This segregation physically prevents the formation of a dense, continuous ice lattice. Instead, it ensures that a significant fraction of the local volume remains in an unfrozen liquid state, which fundamentally limits the source of mechanical strain. Quantitative results of the simulations show that this partial freezing reduces the net volumetric expansion to approximately 4% (Figure 3D), a significant reduction compared to the ∼9% expansion for pure water. Ultimately, the intrinsic reduction in total volumetric expansion, coupled with the stress relaxation facilitated by the liquid network, fundamentally prevents droplet fracturing.

### 2.2 Emergence of Cyclic Levitation

With glycerol suppressing the disruptive self‑propulsion and fracturing events, a striking transition emerges. Glycerol‐water droplets with *x*_g,0_ = 2.40 mol% undergo spontaneous cyclic levitation above the substrate (Figure 1C–E and Movie S1). This behavior differs fundamentally from oscillating Leidenfrost liquid droplets [22] and Leidenfrost‐like trampolining at low pressures [10], as the partially frozen droplet exhibits rigid‐body motion. This is evidenced by its coherent shape during levitation (Figure 1C) and the synchronous change in droplet center height *z* and visible vapor cushion thickness *h* (Figure 1D). Furthermore, the droplet motion is discontinuous and cyclic, comprising four distinct events: dwelling—liftoff—flight—impact (Figure 1D and Movie S1). This discontinuous motion suggests that each launch imparts a discrete impulse, with minimal momentum carried over from the previous flight. Cycle‐to‐cycle variations in the maximum droplet center height reflect fluctuations in initial liftoff velocity, which we attribute to small local changes in composition and contact area in the structurally complex composite droplet (Figure 3) that modulate the instantaneous driving force during liftoff. Notably, the dwelling time (when *h* = 0) shows a trend of increasing with each cycle (Figure 1E), which we explore further later. Compared to the rapid self‐propulsion during recalescence, cyclic levitation involves gentler motions: launching velocities are approximately one order of magnitude smaller (on the order of 0.01 m s^−1^ versus 0.1 m s^−1^ for self‐propulsion, see Movie S2).

The post‐recalescence cyclic levitation reveals itself as a self‐sustaining thermal oscillation, distinct from the self‐propulsion during recalescence. As discussed earlier, the rapid release of latent heat during recalescence creates a macroscopic temperature asymmetry that drives multidirectional self‐propulsion, with direction dictated by the random location of nucleation [14]. After the freezing front traverses the droplet, this global solid/liquid temperature asymmetry largely vanishes, and the motion shifts to nearly vertical kinematics (Figure 1C and Movie S1). This change indicates that the driving force for cyclic levitation is no longer a global latent‐heat‐driven asymmetry, but instead arises from localized dynamics at the droplet base.

Flow‑regime analysis and overpressure modeling clarify the mechanism. Gas dynamics in the confined gap beneath the droplet indicate that our phenomenon occurs in the free molecular flow regime: Kn is at least on the order of 10 over the evaluated vapor temperature and local composition ranges (Section S7). This regime is distinct from the slip‐flow conditions assumed in models of low‐pressure droplet trampolining [10], which attribute overpressure to the viscous resistance of vapor flow through the substrate texture. Extrapolating the slip‐flow model to our conditions substantially overestimates the gap pressure (Section S7), necessitating our development of a free molecular model. Assuming ideal gas behavior in the gap, diffuse reflection from the substrate, and a Hertz‐Knudsen‐Schrage vaporization flux, we balance mass flux into the gap via basal vaporization (${\dot{m}}_{\mathrm{in}}$) against effusive loss out of the gap (${\dot{m}}_{\mathrm{out}}$). This yields a first‐order differential equation for the gap pressure *P*_gap_: $\frac{dP_{\mathrm{gap}}}{dt}\propto \left({\dot{m}}_{\mathrm{in}}-{\dot{m}}_{\mathrm{out}}\right)$. This equation predicts a rapid convergence to an equilibrium gap pressure *P*_gap, equ_ on sub‐microsecond timescales (Figure S5), justifying a quasi‐static approximation for the instantaneous driving pressure.

**FIGURE 4:**
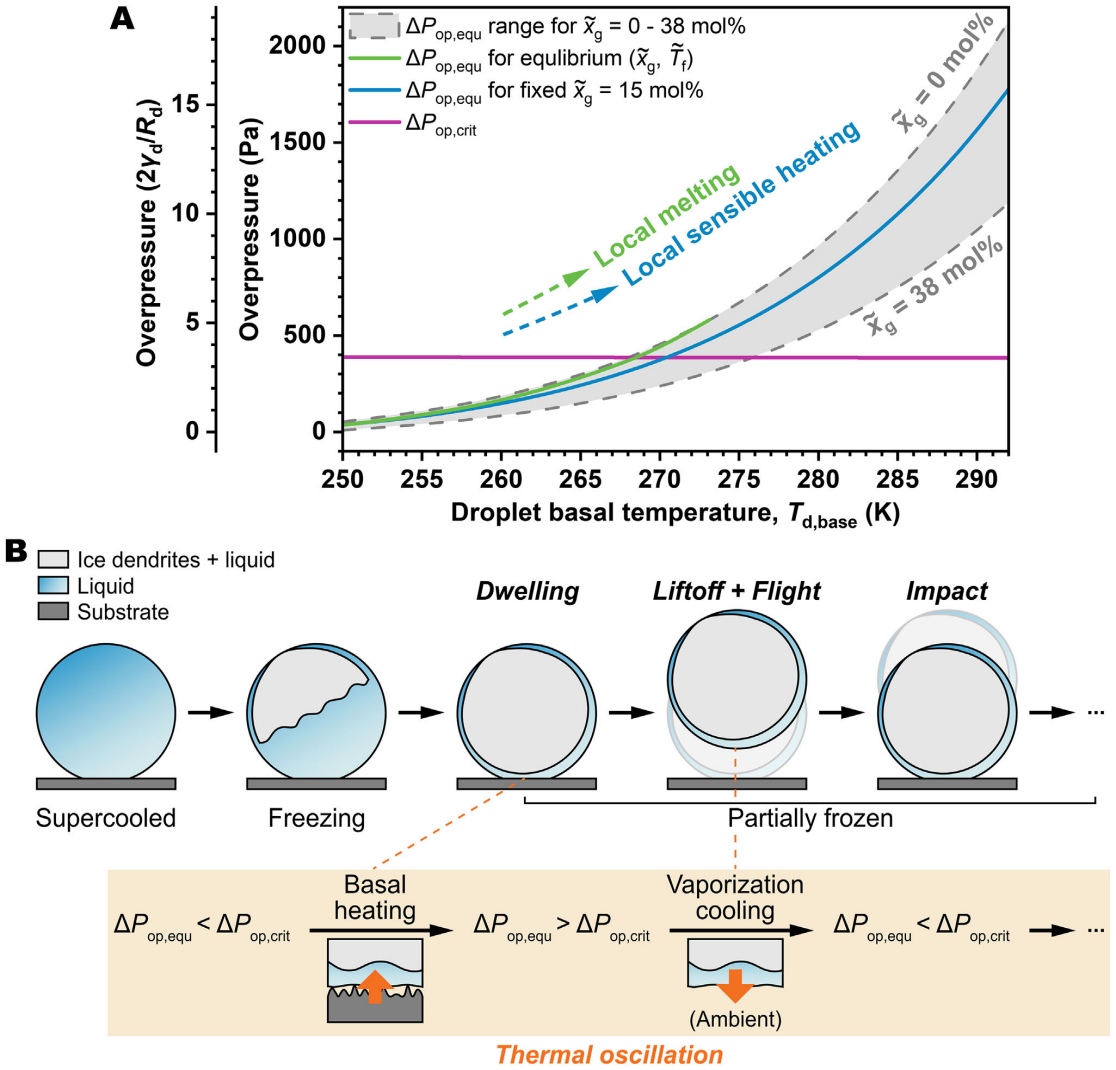
Modeling cyclic levitation. (A) Modeled equilibrium overpressure Δ*P*_op, equ_ and critical overpressure Δ*P*_op, crit_ as functions of droplet basal temperature *T*_d, base_, for varying local glycerol mole fraction ${\tilde{x}}_{g}$ in the droplet's basal liquid layer. Overpressures are plotted in Pascals (Pa) and non‐dimensionalized by 2*γ*_d_/*R*_d_ (evaluated at 265 K and glycerol mole fraction of 15 mol%, for magnitude comparison only). Fixed parameters highlight the effects of variables *T*_d, base_ and ${\tilde{x}}_{g}$, representing the partially frozen droplet in Figure 1C with equivalent droplet radius *R*_d_ = 1.26 mm, contact radius *R*_c_ = 0.293 mm, droplet‐average density *ρ*_d_ = 1030 kg m^−3^, bulk droplet temperature *T*_d, bulk_ = 265 K, bulk substrate temperature *T*_s_ = 292 K, receding contact angle *θ*_r_ = 150°, and chamber pressure *P*_∞_ = 50 Pa. The gray‐shaded region indicates the estimated Δ*P*_op, equ_ range, bounded by ${\tilde{x}}_{g}$ = 0 mol% (pure water) and 38 mol% (based on freeze‐concentration limits [25, 28, 29]). The green line shows the local melting path in increasing Δ*P*_op, equ_, where ${\tilde{x}}_{g}$ is dependent on freezing equilibrium when *T*_d, base_ varies ($T_{d,\mathrm{base}}\approx T_{f}\left({\tilde{x}}_{g}\right)$), with the endpoint at ${\tilde{x}}_{g}$ = 0 mol%, *T*_d, base_ = 273.15 K. The blue line shows the local sensible heating path in increasing Δ*P*_op, equ_, where ${\tilde{x}}_{g}$ remains constant as *T*_d, base_ varies (using ${\tilde{x}}_{g}=15\;\mathrm{mol}\%$ as an example). The magenta line shows Δ*P*_op, crit_ at ${\tilde{x}}_{g}=15\;\mathrm{mol}\%$, which varies negligibly across ${\tilde{x}}_{g}$. Dashed arrows schematically illustrate the Δ*P*_op, equ_ increase for each mechanism. (B) Summary schematic (not to scale) of the cyclic levitation driven by thermal oscillation. The composite structure of the partially frozen droplet results from freeze‐concentration (see Figure 3).

Consequently, the liftoff is characterized by an equilibrium overpressure Δ*P*_op, equ_ in the gap, defined as the difference between *P*_gap, equ_ and the chamber pressure *P_∞_*: $\Delta \;P_{\mathrm{op},\mathrm{equ}}=P_{\mathrm{gap},\mathrm{equ}}\;-P_{\infty }\approx \sqrt{T_{s}/T_{d,\mathrm{base}}}\cdot P_{v}\left({\tilde{x}}_{g},T_{d,\mathrm{base}}\right)-P_{\infty }$, where *T*_s_ is the substrate temperature, and $P_{v}\left({\tilde{x}}_{g},T_{d,\mathrm{base}}\right)$ is the vapor pressure at the droplet base, which depends on the local glycerol mole fraction ${\tilde{x}}_{g}$ and *T*_d, base_. Non‐dimensionalizing Δ*P*_op, equ_ by 2*γ*_d_/*R*_d_ (where *γ*_d_ is the surface tension of glycerol‐water and *R*_d_ is the equivalent droplet radius, see Section S7 and S2 for *γ*_d_ and *R*_d_, respectively) shows that Δ*P*_op, equ_ is of the same order as 2*γ*_d_/*R*_d_ (Figure 4A), comparable to the order reported for droplet trampolining [10].

Liftoff occurs when the upward overpressure force acting on the contact area, *F*_op_ =  π*R*_c_^2^Δ*P*_op, equ_, exceeds the combined resistances of gravity *F*_g_, substrate adhesion *F*_a_, and a net downward vaporization recoil pressure *P*_r_, where *R*_c_ is the contact radius (see Section S7 for details). This defines a critical overpressure for liftoff, Δ *P*_op, crit_ = (*F*_g_ + *F*_a_)/π*R*_c_^2^  + *P*_r_, which Δ*P*_op, equ_ must surpass to trigger launch. As illustrated in Figure 4A, for a quasi‐steady bulk substrate temperature *T*_s_ and chamber pressure *P*_∞_, the model shows that Δ*P*_op, equ_ increases as the local glycerol mole fraction ${\tilde{x}}_{g}$ decreases and as the droplet basal temperature *T*_d, base_ increases, highlighting the crucial role of local dynamics at the droplet base.

Our thermal model (Section S8) identifies the heat pathways controlling basal temperature during dwelling: solid–liquid conduction at the contact points and free‐molecular gas conduction across the intervening gap dominate the basal heat flux, while vaporization cooling at the base is negligible once *P*_gap_ has equilibrated. Given the composite structure of the partially frozen droplet, two mechanisms could, in principle, produce sufficient Δ*P*_op, equ_ for liftoff: (i) local sensible heating of the basal liquid layer (i.e., a temperature rise without phase change), which raises *T*_d, base_ at effectively constant composition (represented by the blue line in Figure 4A); and (ii) local melting of ice near the base, which dilutes the local glycerol concentration and raises the equilibrium freezing temperature (depicted by the green line in Figure 4A).

We compared the characteristic timescales required for each mechanism to produce a target liftoff overpressure (e.g., Δ*P*_op, equ_ = 400 Pa). By treating the substrate and the droplet basal liquid as semi‐infinite bodies over the short dwelling intervals and assuming ideal solution behavior, we find that sensible heating raises *T*_d, base_ on timescales ranging from milliseconds to tens of milliseconds (Figure S6B), consistent with observed dwelling times (Figure 1E). By contrast, local melting requires supplying the latent heat of fusion in addition to conducting heat into the bulk ice, yielding timescales orders of magnitude longer (Figure S6C). Thus, basal sensible heating is the dominant trigger for liftoff and occurs well before significant melting can contribute. The assumed pre‐dwelling basal temperature changes the calculated heating time, but it does not change the order‐of‐magnitude comparison and the modeled sequence in which basal heat input raises *T*_d, base_ and Δ*P*_op, equ_ until the liftoff criterion is reached.

Subsequently, during the droplet's flight, the spatial confinement is lost as the gap widens, causing the vapor cushion to dissipate. Concurrently, basal heating is cut off, while the vaporization cooling proceeds at a flux comparable to the prior dwelling heat influx (Section S8). This indicates that vaporization cooling is equally effective in restoring the droplet basal properties (e.g., via sensible heat removal or local re‐freezing) over the 10‐millisecond‐scale flight time, resetting the conditions for the next cycle. Notably, continuous vaporization from the droplet's free surface gradually lowers the bulk droplet temperature *T*_d, bulk_ following recalescence (Figure S1B). A declining *T*_d, bulk_ reduces the pre‐dwelling basal temperature upon each successive landing, while each liftoff requires the droplet base to reach a specific target temperature to generate the necessary critical overpressure Δ*P*_op, crit_ (Figure S6). Consequently, the required basal temperature rise and thus the required dwelling duration increase with cycle number. While the fluctuations in the local composition and contact area introduce cycle‐to‐cycle noise, the progressive cooling of the droplet explains the observed increase in dwelling time over successive cycles (Figure 1E). This cooling trend also explains the short‐dwelling transient shortly after recalescence (the first ∼700 ms in Figure 1D,E): while *T*_d, bulk_ remains elevated (Figure S1B), only a small basal temperature rise is required, yielding short but resolvable millisecond‐scale dwelling intervals.

Based on our analysis, we describe the observed cyclic levitation as a self‐sustaining thermal oscillation (Figure 4B):

1. Dwelling: Upon contact, rapid basal sensible heating raises the equilibrium overpressure Δ*P*_op, equ_ until it exceeds the critical overpressure Δ*P*_op, crit_.
2. Liftoff and flight: The droplet launches ballistically while its base cools by vaporization, restoring its pre‐dwelling state.
3. Impact: The droplet lands with an insufficient Δ*P*_op, equ_ to relaunch immediately. The cycle repeats.

This framework captures the essential mechanics and time scales of the observed cyclic levitation and highlights the primacy of localized basal heating and free‑molecular dynamics in driving the phenomenon.

### 2.3 Regime Transitions in Vaporization‐Driven Dynamics

**FIGURE 5:**
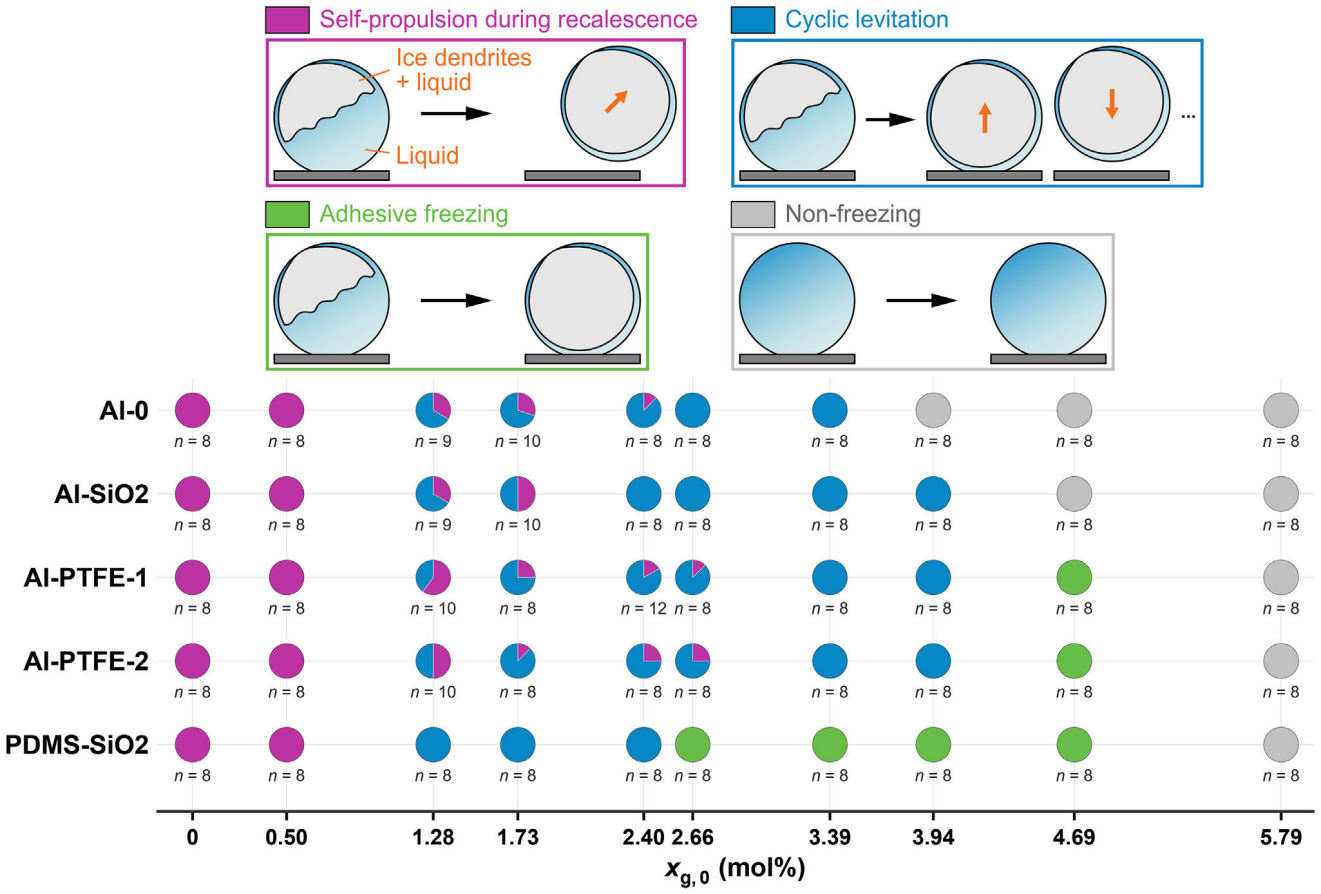
Regime map of vaporization‐driven droplet dynamics. Statistical outcomes are reported for different initial glycerol mole fractions *x*_g,0_ (*x*_g,0_ = 0 for pure water), on different superhydrophobic substrates under a standardized depressurization process (see Section S1 and ‘Experimental Process’ in Materials and Methods). Each colored pie segment gives the observed relative frequency of one of four mutually exclusive outcomes: self‐propulsion during recalescence, cyclic levitation, adhesive freezing without liftoff, or non‐freezing. The number of independent experiments *n* is displayed beneath each pie chart. Schematics in the legend illustrate the distinct dynamics for each outcome (not to scale), simplifying the depiction to the composite freezing structures of the glycerol‐water droplets. Orange arrows indicate directions of motion. For pure water droplets, self‐propulsion is consistently followed by droplet breakup within seconds, whereas no fracturing was observed for any glycerol‐water droplets.

To elucidate how glycerol concentration affects vaporization‐driven dynamics, we mapped the dynamical outcomes of droplet freezing across varying *x*_g,0_ on different superhydrophobic substrates (Figure 5, see also Movie S2). Cyclic levitation proves to be a robust and reproducible outcome on all tested substrates once the disruptive instabilities are suppressed. Consistent with the mechanistic analysis in Section S6, the frequency of self‐propulsion decreases with increasing *x*_g,0_, reflecting the reduction in the vaporization recoil force as the equilibrium freezing temperature is reduced. When initial self‐propulsion is avoided, cyclic levitation reliably occurs at low *x*_g,0_. In rare cases where a pure water droplet returns to the substrate after a self‐propulsion event, it subsequently enters the cyclic levitation state temporarily, until eventual breakup (Movie S4). This further indicates that suppression of disruptive events is a necessary condition for cyclic levitation.

The regime map (Figure 5) identifies two additional outcomes, observed only at higher *x*_g,0_: adhesive freezing (no liftoff) and non‐freezing. Adhesive freezing occurs when the overpressure generated at the droplet base remains insufficient to overcome gravity and substrate adhesion; the droplet solidifies in place without launching. Non‐freezing occurs when the freezing point depression at high *x*_g,0_ prevents ice nucleation under our experimental conditions. The relative occurrences of these two regimes vary across substrates, suggesting sensitivity to substrate‐specific properties at higher *x*_g,0_, such as surface wettability and effective thermal effusivity. While our current experiments were conducted exclusively on superhydrophobic surfaces, we expect the regime boundaries to shift on more wetting surfaces. Increased adhesion on higher‑wettability substrates will raise the critical overpressure required for liftoff, favoring adhesive freezing over cyclic levitation. Likewise, for a given droplet, the substrate's effective thermal effusivity at discrete micro/nanoscale contact points governs the transient heat transfer at the droplet base (Section S8), dictating both the initial net cooling rate required to trigger ice nucleation and the subsequent net heating rate required to generate sufficient overpressure.

## 3 Conclusion

We report the discovery and mechanistic analysis of the cyclic levitation of partially frozen glycerol‐water droplets under low‐pressure conditions, illuminating how thermal and compositional factors govern a new class of vaporization‐driven dynamics. This phenomenon emerges from tightly coupled, transient interfacial physics: rapid vaporization cools the droplet, solute rejection sculpts the composite freezing structure, and localized basal heating during dwelling drives cyclic overpressure buildup and ballistic launches. Because these processes evolve together on millisecond timescales and across length scales ranging from nanoscale interfaces to the macroscopic droplet size, fully predictive, quantitative models remain challenging. Two specific sources of uncertainty highlight the need for further probing: (i) the effective thermal effusivity of superhydrophobic surfaces; and (ii) the dynamic wetting states and resulting adhesion forces at low pressures. Nevertheless, our qualitative insights offer a foundational understanding that will guide future efforts toward more refined models and targeted interfacial experiments.

Although our experiments focus on the glycerol‐water binary system, the controlling mechanisms point to broader applicability across other aqueous solutions, provided key thermophysical requirements are met. Effective compositional tuning should satisfy three criteria: (i) sufficient depression of equilibrium freezing temperature to suppress self‐propulsion; (ii) a freezing morphology that preserves sufficient peripheral liquid to facilitate efficient sensible heating while maintaining an adequate substrate contact area to lower the critical overpressure for liftoff; and (iii) adequate surface tension to avoid premature trampolining of unfrozen droplets. Reductions in surface tension or increases in contact radius raise the dimensionless force ratio governing liquid droplet trampolining [10], potentially causing trampolining before nucleation and thereby disrupting cyclic levitation. Notably, preliminary tests with other aqueous solutions that meet these criteria (for example, glucose‐water) exhibit similar cyclic levitation behavior (Movie S6). This suggests that compositional tuning is an extensible approach for controlling low‐pressure multiphase dynamics.

Beyond basic fluid mechanics, the coupled control of ice morphology, solute redistribution, and vapor‐driven motion has implications for controlled freezing and low‐pressure systems. In cryopreservation, glycerol and other cryoprotective solutes are used to suppress damaging ice formation [1]. Although our findings are based on partial freezing, the solute redistribution and freezing morphology reported in this work may inform cryopreservation‐related design considerations. Related control of solute segregation and ice structure is central to freeze‐drying operations in material processing [2, 3, 4], where these factors affect concentration distributions and the pore structure produced. On the engineering side, adjusting working‐fluid composition to moderate expansion‐induced failure and preserve controlled detachment may improve the reliability of low‐pressure thermal‐management devices, including spacecraft flash evaporators in which ice accumulation and nozzle blockage are important failure modes [30]. On the planetary science side, these experiments offer a valuable laboratory analog for vaporization‑driven levitation phenomena observed on airless or low‑pressure bodies. For example, in the Martian south polar cap, seasonal CO_2_ ice deposited during winter forms translucent slabs; during warmer seasons, solar energy transmitted through the ice drives basal sublimation and overpressure accumulation to lift and eventually rupture the slabs [31]. Although the materials and timescales differ, the present system provides a tunable platform for examining the common coupling among basal heat input, vapor generation, and pressure‐driven rigid‐body motion.

## 4 Materials and Methods

### 4.1 Materials

Water solutions of glycerol (99.5%, CAS#56‐81‐5, Sigma‐Aldrich) with initial glycerol mole fractions *x*_g,0_ ranging from 0.50 to 4.69 mol% were prepared as experimental fluids. Droplets with *x*_g,0_ = 2.40 mol% were primarily reported and discussed in this work, while the other glycerol mole fractions (0.50, 1.28, 1.73, 2.66, 3.39, 3.94, and 4.69 mol%) were used for comparative experiments. Additionally, water solution of D‐(+)‐glucose (99.5%, CAS#50‐99‐7, Sigma‐Aldrich) with initial glucose mole fraction of 1.44 mol% was prepared for comparative experiment. Deionized (DI) water was used both as the solvent and as an experimental fluid. All the experimental fluids were degassed by sonication for 10 min before experiments.

Various superhydrophobic structured substrates were used in this work. The use of superhydrophobic substrates allows us to focus on droplet dynamics while minimizing the effects of droplet morphology and substrate wettability. Droplets on aluminum hierarchical structured substrate (Al‐0) were primarily reported and discussed in this work. The other substrates include aluminum substrate coated with SiO_2_ nanoparticles (Al‐SiO_2_), aluminum substrate coated with polytetrafluoroethylene nanoparticles (Al‐PTFE‐1 and Al‐PTFE‐2), and polydimethylsiloxane micropillar substrate coated with SiO_2_ nanoparticles (PDMS‐SiO_2_). See Section S1 and Table S1 for detailed fabrication and characterization.

### 4.2 Experimental Process

All experiments commenced at room temperature (295 ± 1 K) and standard atmospheric pressure (101.3 kPa). A superhydrophobic substrate was horizontally positioned (± 0.2°) on an aluminum alloy xy‐axis stage inside a stainless‐steel vacuum chamber (Figure S1A). A droplet (∼10 µL) of the experimental fluid, equilibrated to room temperature, was dispensed onto the substrate using a precision pipette (10 µL, Eppendorf).

The chamber pressure was reduced from atmospheric pressure to below 100 Pa within ∼20 s (Figure S1C) using a vacuum pump (YL90‐4, Value, China). This rapid depressurization rate effectively minimized non‐condensable gases before the onset of nucleation, reducing their potential influence on the vaporization process [14]. Chamber pressure was monitored with a vacuum gauge (Type 0531 TC, Agilent), while relative humidity was measured using a humidity sensor (HM1500LF). Bulk droplet temperature was monitored for the Al‐0 substrate, which serves as the representative case for all aluminum‐based substrates in this study (Al‐0, Al‐SiO_2_, Al‐PTFE‐1, Al‐PTFE‐2). A T‐type thermocouple (0.5 mm probe diameter) was inserted through a horizontally drilled hole to the horizontal center position of the substrate, with the tip positioned approximately 0.75 mm below the upper surface. To validate this measurement, the Biot number was estimated as $\mathrm{Bi}\approx \left({\bar{h}}_{\mathrm{in}}\times 0.75\times 10^{-3}\;m\right)/\left(237\;W\;m^{-1}K^{-1}\right)\ll 1$, where ${\bar{h}}_{\mathrm{in}}$ represents the time‐averaged effective heat influx coefficient (*h*_in_(*t*)) for droplet basal heating over the dwelling time (derived from Section S8), and the thermal conductivity of the aluminum bulk is used. The low Biot number indicates a nearly uniform temperature distribution between the measurement point and the substrate surface. Thus, the measured temperature accurately represents the bulk substrate temperature *T*_s_ used in the thermal model (Section S8) for all aluminum‐based substrates.

The visual dynamics were captured from side‐view using a high‐speed camera (FASTCAM Nova S12, Photron) equipped with microlenses (Navitar 1–62922 and 1–50486, offering magnification up to 12×). Frame rates ranged from 500 to 2000 fps. Illumination was provided by an LED light source facing the droplet, with a diffuse reflector placed behind the droplet. Data processing was performed using PFV, Image‐Pro Plus, and ImageJ software.

Infrared (IR) imaging was conducted from side‐view through a sapphire window using an infrared camera (FLIR SC7700BB, Teledyne) working in mid‐IR (1.5–5 µm) at frame rates ranging from 115 to 412 fps. Data processing was performed using FLIR Altair software.

### 4.3 Quasi‐Two‐Dimensional (Quasi‐2D) Droplet

We modified the Hele‐Shaw apparatus [32] to generate a quasi‐2D droplet for qualitative analysis of the droplet internal structure under reduced pressure. In this configuration, a ∼10 µL droplet was placed at the intersection of the horizontal Al‐0 substrate and a vertically oriented silica glass slide. The droplet's interface contacting the glass slide faced the high‐speed camera for imaging. The conventional Hele‐Shaw apparatus, typically comprising two parallel glass slides, proved unsuitable under reduced pressure due to the substantial evaporation of water vapor, which disrupted droplet integrity. This configuration introduced additional heat conduction from the glass slide and reduced the droplet's free surface area, necessitating the use of lower *x*_g,0_ in the glycerol‐water solution to achieve droplet freezing under the same depressurization process. All other experimental protocols remained consistent with those described in the ‘Experimental Process’ section.

## Author Contributions

C.X., X.Y., and S.Y. conceived the research. S.Y. supervised this work. C.X., W.M., Q.Z., X.M., and X.Y. fabricated the substrates. C.X. and W.M. characterized the substrates. C.X., D.L., and X.L. performed the experiments. R.H. and Y.D. performed the MD simulations. C.X., R.H., W.M., F.C., X.Y., and S.Y. analyzed the results. C.X., W.M., X.Y., and S.Y. developed the theoretical models. C.X., R.H., W.M., F.C., X.Y., and S.Y. led the manuscript preparation with input from all the authors.

## Funding

S.Y. and C.X. gratefully acknowledge support from Research Grants Council of Hong Kong (General Research Fund, No. 16213721). X.Y. acknowledges support from Young Scientists Fund of the National Natural Science Foundation of China (No. 52406180) and Chongqing Excellent Young Scientists Fund (No. CSTB2024YCJHKYXM0081).

## Conflicts of Interest

The authors declare no conflicts of interest.

## Supporting information




**Supporting File 1**: advs77772‐sup‐0001‐SuppMat.pdf.

**Supporting File 2**: advs77772‐sup‐0002‐MovieS1.mp4.

**Supporting File 3**: advs77772‐sup‐0003‐MovieS2.mp4.

**Supporting File 4**: advs77772‐sup‐0004‐MovieS3.mp4.

**Supporting File 5**: advs77772‐sup‐0005‐MovieS4.mp4.

**Supporting File 6**: advs77772‐sup‐0006‐MovieS5.mp4.

**Supporting File 7**: advs77772‐sup‐0007‐MovieS6.mp4.

## Data Availability

All the data that support the findings of this study are available in the main text or the supplementary materials. The MATLAB codes used in analysis have been deposited in Zenodo [33] (https://doi.org/10.5281/zenodo.18342636).
