# Supplementary material for: Spontaneous Cyclic Levitation of Glycerol‐Water Droplets at Low Pressures
Source: Adv Sci (Weinh). 2026 Sep 13:e77772. Online ahead of print. doi: 10.1002/advs.77772 (PMC13572865; doi:10.1002/advs.77772)
Supplement: Supplementary file 1 [file ADVS-9999-e77772-s003.pdf]

# Supporting Information

## **Spontaneous Cyclic Levitation of Glycerol-Water Droplets at Low Pressures**

*Chen Xu, Renjie Hua, Wei Ma, Fuqiang Chu, Dong Liang, Xueshi Li, Qirui Zhang, Yuan Dong, Xuehu Ma, Xiao Yan*, Shuhuai Yao**

*Corresponding authors:

Xiao Yan (Email: yanx23@cqu.edu.cn)

Shuhuai Yao (Email: meshyao@ust.hk)

## TABLE OF CONTENTS

TABLE OF CONTENTS .......... 2
Supplementary Section 1. Substrate Fabrication and Characterization .......... 4
Supplementary Section 2. Droplet Characterization .......... 7
Supplementary Section 3. Convective Flow in the Unfrozen Droplet .......... 10
Supplementary Section 4. Modeling the Convective Flow in the Unfrozen Droplet .......... 13
Supplementary Section 5. Molecular Dynamics (MD) Simulation .......... 17
Supplementary Section 6. Vaporization Force and Self-Propulsion .......... 22
Supplementary Section 7. Modeling Overpressure Dominated by Free Molecular Flow .......... 28
Supplementary Section 8. Modeling Heat Transfer at Droplet Base During Dwelling .......... 34
Supplementary Section 9. Exclusion of Liquid-Liquid or Fragile-Strong Transition .......... 42
Supplementary Section 10. Nomenclature .......... 45
SUPPLEMENTARY REFERENCES .......... 48

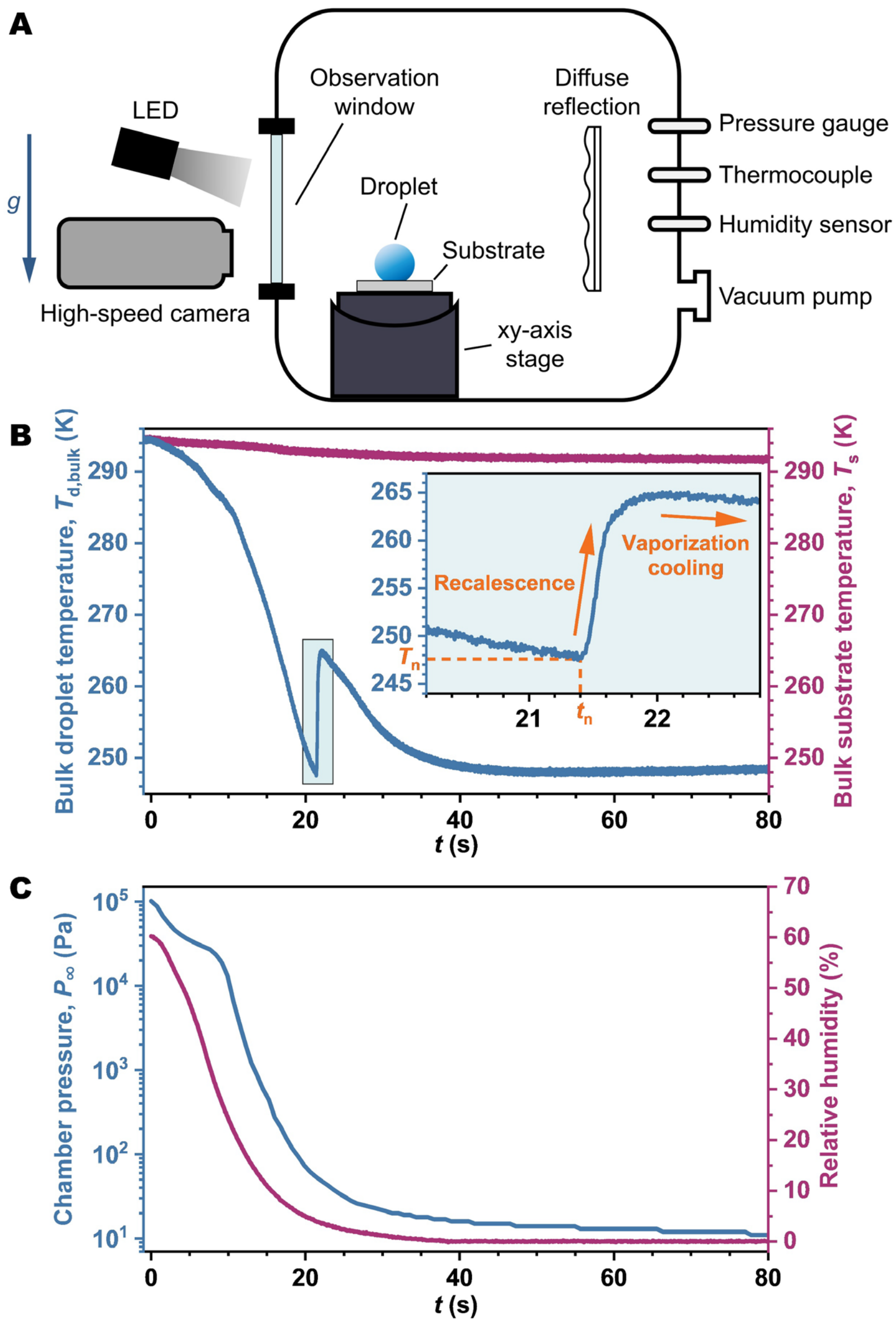


Figure S1. Experimental setup and process. **(A)** Schematic of the primary experimental setup (not to scale). The high-speed camera captures the droplet dynamics from side-view. **(B)** Time-dependent bulk droplet temperature $T_{d,bulk}$ and bulk substrate temperature $T_s$ as measured by thermocouple for a glycerol-water droplet with $x_{g,0}$ = 2.40 mol% on Al-0 substrate. Time $t = 0$ denotes the onset of depressurization, and $t = t_n$ denotes the moment of nucleation. Following the initiation of nucleation at temperature $T_n$, a sharp rise in $T_{d,bulk}$ (i.e., recalescence) was observed, followed by a subsequent temperature drop due to continuous vaporization cooling. Concurrently, $T_s$ remained at approximately 292 K or higher, maintaining a significant temperature difference from the droplet. **(C)** Time-dependent chamber pressure and relative

humidity during a standardized depressurization process (see ‘Experimental Process’ in Materials and Methods).

## Supplementary Section 1. Substrate Fabrication and Characterization

Aluminum hierarchical structured substrate (Al-0) was fabricated from plain aluminum plates (99.99%) via chemical etching. The aluminum plates were ultrasonically cleaned in acetone, ethanol, and deionized (DI) water for 5 min each. The hierarchical structure was formed by immersing the cleaned plates in a 0.05 mol/L NaOH (CAS#1310-73-2, Thermo Fisher) solution at 333 K for 5 minutes, followed by immersion in DI water at 363 K for 30 min. The substrate was dried in an atmospheric pressure oven at 363 K for 30 min and cooled to ambient temperature. Subsequently, the substrate was immersed in a 0.5 wt% solution of 1H,1H,2H,2H-perfluorodecyltriethoxysilane (97%, CAS#101947-16-4, Sigma-Aldrich) in n-hexane (95%, CAS#110-54-3, Tedia) for 2h. After drying at 393 K for 30 min, the superhydrophobic surface was obtained.

Aluminum substrate coated with $SiO_2$ nanoparticles (Al-SiO2) was fabricated via spray coating. $SiO_2$ nanospheres (diameters of 500 nm and 80 nm, Chaowei Nami, Dongguan, China) were dispersed in a 0.5 wt% solution of 1H,1H,2H,2H-perfluorodecyltriethoxysilane in n-hexane for 1h, followed by drying the mixture at 393 K for 2 hours. The dried powder was then mixed with ethanol (96%, CAS#64-17-5, VWR Chemicals) to form a 1 wt% $SiO_2$ nanosphere solution, stirred at 1500 rpm for 2 hours. Cleaned plain aluminum plates, pretreated with a 2-minute plasma treatment at 38 W (PDC-001, Harrick Plasma), were positioned perpendicular to the spray nozzle on a vacuum stage. The $SiO_2$ nanoparticles were deposited using an ultrasonic spray system (UC 330, Siansonic Technology) equipped with a nozzle operating at a frequency of 45 kHz (Z402, Siansonic Technology). The distance between the nozzle tip and the substrate was maintained at 50 mm, with nozzle speed of 4 mm/s, bulk substrate temperature of 295 K, shaping air pressure of 0.015 MPa, and solution flow rate of 0.1 mL/min. The superhydrophobic surface was obtained after eight cycles of spray coating.

Aluminum substrates coated with polytetrafluoroethylene (PTFE) nanoparticles (Al-PTFE-1 and Al-PTFE-2) were fabricated via spray coating. To improve the solubility of PTFE nanoparticles,

a fluorinated acrylic copolymer (ST-110, Chemours) was used as a dispersing agent. The PTFE coatings for Al-PTFE-1 and Al-PTFE-2 were prepared by mixing 50 nm PTFE nanoparticles (Qihong Plastic, Dongguan, China) with fluorinated acrylic copolymer in mass ratios of 1:1 and 4:1, respectively. The mixtures were mixed with ethanol to form a 2 wt% solution. The solution was stirred with a magnetic stirrer for 4 hours to ensure uniform dispersion. Before spray coating, the air pressure was set to 0.5 MPa. The solution was sprayed onto cleaned and plasma-treated plain aluminum plates using a spray gun (V3/0.8, Meiji) from a distance of 15 cm. The coated substrates were dried at 373 K for 30 minutes, yielding superhydrophobicity.

Polydimethylsiloxane (PDMS) micropillar substrate coated with $SiO_2$ nanoparticles (PDMS-SiO2) was fabricated via molding and spray coating. The 304 stainless-steel micro-latticed mold was processed using a nanosecond fiber laser system (HM20, Han's Laser, China) with wavelength of 1064 nm, output power of 20 W, pulse duration of 200 ns, and repetition rate of 40 kHz. A focused laser beam was delivered through a scanning head equipped with a focusing lens. The laser scanned a polished stainless-steel plate at 300 mm/s along the x-axis (0°) and y-axis (90°). Subsequently, PDMS prepolymer (SYLGARD 184 Silicone Elastomer Kit, Dow Corning) was mixed with the curing agent at a mass ratio of 10:1. The mixture was cast onto the mold, degassed, and cured at 353K for 1h. After demolding, the PDMS substrate was spray-coated with $SiO_2$ nanospheres (500 nm and 80 nm) following the same protocol as the Al-SiO2 substrate, resulting in a superhydrophobic surface.

The surface structures of the substrates were characterized by scanning electron microscopy (JSM-6390, JEOL), atomic force microscope (Dimension Icon AFM, Bruker), and 3D optical profilometer (NPFLEX-1000, Bruker). The wettability (contact angle and receding contact angle) was characterized by a station contact angle meter (Theta Topography, Biolin Scientific) at room temperature (295 K) and standard atmospheric pressure (101.3 kPa). In wettability tests, deionized (DI) water droplets of 5 μL were dripped at different locations on the substrate. The average values and the standard deviations were obtained from three independent measurements. Table S1 provides a summary of substrate parameters. Table S2 provides the intrinsic thermal properties of substrate components. The intrinsic thermal effusivity is defined as $e = \sqrt{\Lambda \rho c_\mathrm{p}}$, where $\Lambda$ is the thermal conductivity, $\rho$ is the density, and $c_\mathrm{p}$ is the isobaric heat capacity.

Table S1. Primary materials, processing methods, functional components, surface structural height ($H_{struc}$), contact angle, receding contact angle ($\theta_r$), and scanning electron microscopy (SEM) images of the substrates used in this work. The surface structural height is defined as the bearing depth difference between the 1% to 99% bearing area to facilitate gap height estimation in overpressure modeling. Surface structural heights were measured using an atomic force microscope (Dimension Icon AFM, Bruker) for the Al-0 and Al-SiO2 substrate, and a 3D optical profilometer (NPFLEX-1000, Bruker) for the rest of the substrates.

| **Substrate** | **Primary material** | **Processing** | **Functional component** | **Surface structural height, $H_{struc}$** | **Contact angle [deg]** | **Receding contact angle, $\theta_r$ [deg]** | **SEM images** |
|---|---|---|---|---|---|---|---|
| Al-0 | Aluminum | Etching | N/A | 973 nm | 165.0 ± 1.9 | 150.3 ± 6.5 | 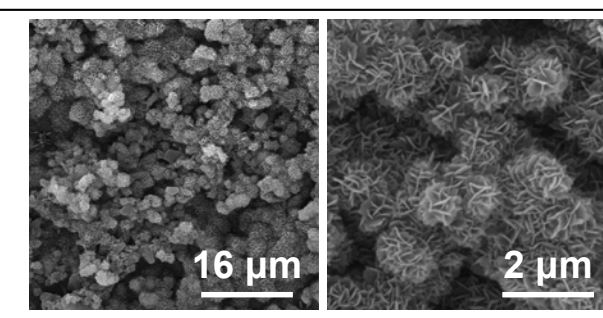 |
| Al-SiO2 | Aluminum | Spray coating | $SiO_2$ nanoparticles | 463 nm | 166.9 ± 2.0 | 161.6 ± 1.5 | 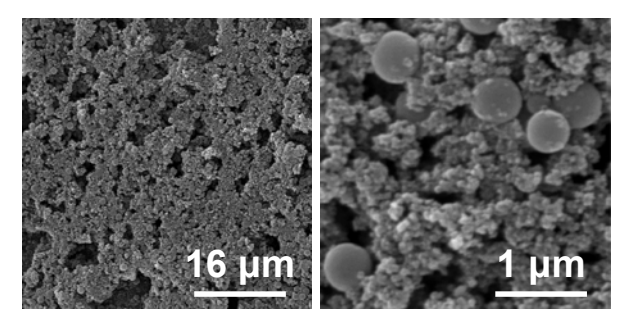 |
| Al-PTFE-1 | Aluminum | Spray coating | PTFE nanoparticles | 7.34 µm | 169.4 ± 1.0 | 158.6 ± 1.3 | 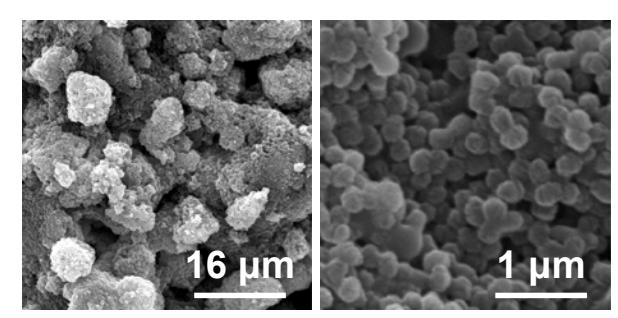 |
| Al-PTFE-2 | Aluminum | Spray coating | PTFE nanoparticles | 17.7 µm | 170.0 ± 1.7 | 159.7 ± 3.7 | 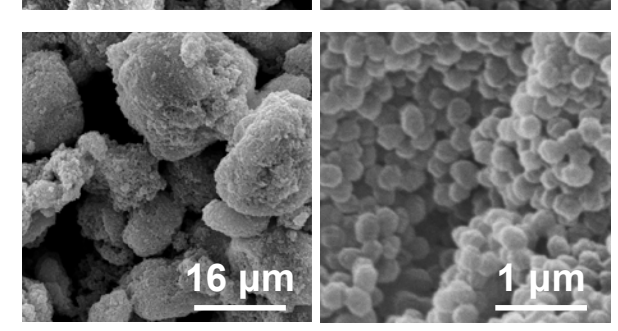 |
| PDMS-SiO2 | PDMS | Molding and spray coating | $SiO_2$ nanoparticles | 28.8 µm | 170.5 ± 1.3 | 145.0 ± 8.4 | 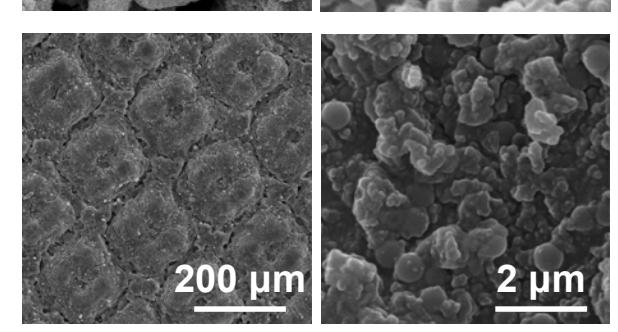 |

Table S2. Intrinsic thermal properties of substrate components, including thermal conductivities ($\Lambda$), densities ($\rho$), isobaric heat capacities ($c_p$), thermal effusivities ($e$), and thermal diffusivities ($\alpha$) at room temperature.

| Material | Thermal conductivity, $\Lambda$ [W m$^{-1}$ K$^{-1}$] | Density, $\rho$ [kg m$^{-3}$] | Isobaric heat capacity, $c_p$ [J kg$^{-1}$ K$^{-1}$] | Thermal effusivity, $e$ [W s$^{0.5}$ m$^{-2}$ K$^{-1}$] | Thermal diffusivity, $\alpha$ [m$^2$ s$^{-1}$] |
|---|---|---|---|---|---|
| Aluminum[1] | 236 | 2710 | 898 | $2.40 \times 10^4$ | $9.70 \times 10^{-5}$ |
| PDMS[2] | 0.15 | 970 | 1460 | $4.61 \times 10^2$ | $1.06 \times 10^{-7}$ |
| $SiO_2$[3] | 1.34 | 2210 | 725 | $1.47 \times 10^3$ | $8.36 \times 10^{-7}$ |
| PTFE[2] | 0.4 | 2300 | 1030 | $9.73 \times 10^2$ | $1.69 \times 10^{-7}$ |

## Supplementary Section 2. Droplet Characterization

We estimated droplet-average properties before nucleation by analyzing temporal changes in side-view images. To calculate the droplet volume $V_d$ and droplet surface area $A$, the morphology of an unfrozen droplet was approximated as a truncated ellipsoid with equal major axes, truncated along a plane perpendicular to the minor axis. Major and minor axis lengths were measured from side-view high-speed images. Unless otherwise specified, subscripts denote: g for glycerol, w for liquid water, i for ice, d for droplet-average properties, and 0 for initial conditions.

For a droplet with initial glycerol mole fraction $x_{g,0}$, we determined the initial droplet volume $V_{d,0}$, the droplet volume $V_d$ at a given time, and the bulk droplet temperature $T_{d,bulk}$ measured via thermocouple. The following simplifying assumptions were made when estimating droplet-average or local physical properties: negligible glycerol evaporation under experimental conditions[4], the liquid phase consists of ideal solutions of incompressible fluids, and negligible effects of local temperature differences.

Then, $V_{d,0}$ is expressed as:

$$V_{d,0} = \frac{n_{g,0}M_g}{\rho_{g,0}} + \frac{n_{w,0}M_w}{\rho_{w,0}} = \left[\frac{M_g}{\rho_{g,0}} + \frac{1 - x_{g,0}}{x_{g,0}} \cdot \frac{M_w}{\rho_{w,0}}\right] n_{g,0} \tag{S1}$$

where *n*, *M*, *ρ* are the molar quantity, molar mass, and density of the respective pure species, respectively. Densities are estimated using empirical relations[5-7] at atmospheric pressure, with densities in kg m$^{-3}$ and temperature *T* in K:

$$\rho_g = 1.271[1000 - 0.601(T - 273.15)] \ , 220\ \mathrm{K} < T < 310\ \mathrm{K} \tag{S2a}$$

$$\rho_w = \begin{cases} 999.86 + 6.690 \times 10^{-5}(T - 273.15) - 8.486 \times 10^{-6}(T - 273.15)^2 \\ +1.518 \times 10^{-7}(T - 273.15)^3 - 6.9484 \times 10^{-9}(T - 273.15)^4 & , 239\ \mathrm{K} < T \le 273\ \mathrm{K} \\ -3.6449 \times 10^{-10}(T - 273.15)^5 - 7.497 \times 10^{-12}(T - 273.15)^6 \\ 1000\left(1 - \left|\frac{T - 277.13}{615}\right|^{1.71}\right) & , 273\ \mathrm{K} < T < 373\ \mathrm{K} \end{cases}$$

$$(S2b)$$

The initial glycerol molar quantity $n_{g,0}$ and glycerol molar quantity $n_g$ are expressed accordingly: $n_g = n_{g,0} = V_{d,0}/\left(\frac{M_g}{\rho_g} + \frac{1-x_{g,0}}{x_{g,0}} \cdot \frac{M_w}{\rho_w}\right)$. The initial glycerol mass $m_{g,0} = M_g n_{g,0}$, initial water mass $m_{w,0} = M_w n_{g,0}\left(1 - x_{g,0}\right)/x_{g,0}$, glycerol mass $m_g = m_{g,0}$, and water mass $m_w = m_{w,0} - \left(V_{d,0} - V_d\right)\rho_w$ were obtained respectively. Consequently, the following droplet-average properties at a certain time were obtained: glycerol mole fraction $x_g = n_g/\left(n_g + m_w/M_w\right)$, water mass concentration $c_w = m_w/V_d$. The following properties were derived from $x_g$: glycerol mass fraction $w_g = M_g x_g/\left[M_g x_g + M_w(1 - x_g)\right]$, water mass fraction $w_w = 1 - w_g$, and glycerol volume fraction $\varphi_g = x_g M_g \rho_w/\left[x_g M_g \rho_w + \left(1 - x_g\right) M_w \rho_g\right]$.

Specifically, the pre-nucleation properties of the droplet were determined from the frame immediately preceding visible nucleation in high-speed imaging. For simplification of derivations, an equivalent droplet radius $R_d$ was used as the characteristic length of the droplet:

$$R_d = (3V_d/4\pi)^{1/3} \tag{S3}$$

Furthermore, the droplet-substrate contact radius $R_c$ was measured in-situ from the same side-view high-speed image sequences. The contact radius of the partially frozen droplet decreases compared to its unfrozen liquid state (Figure 1), while the droplet exhibits negligible shape

changes once the composite freezing structure is established. For modeling the self-propulsion during recalescence (Supplementary Section 6), we utilized the pre-freezing $R_c$ as a constant geometric approximation during this transient process. Conversely, for modeling the cyclic levitation after recalescence (Supplementary Section 7 - 8), we utilized the post-freezing, stabilized $R_c$ as a constant geometric approximation.

In addition, we estimated the experimental value of vaporization mass rate $\dot{m}_{\mathrm{vap}}$ at time $t$ as the average rate of water mass decrease over the interval $t \pm 0.2$ s: $\dot{m}_{\mathrm{vap}} \approx \Delta m_{\mathrm{w}}/\Delta t$. Subsequently, the experimental value of vaporization mass flux $J_{\mathrm{vap}} \approx \dot{m}_{\mathrm{vap}}/A$ was derived from the mass rate by approximating a surface-average value and neglecting the surface area change during this time interval. Specifically, the experimental values of $\dot{m}_{\mathrm{vap}}$ and $J_{\mathrm{vap}}$ prior to nucleation were estimated based on the average rate of water mass decrease from $t - 0.4$ s to $t$.

Thermal properties of glycerol-water are frequently used in this work, and we summarize them here. The thermal diffusivity $\alpha_{\mathrm{d}}$ and thermal effusivity $e_{\mathrm{d}}$ of glycerol-water are defined as:

$$\alpha_{\mathrm{d}} = \Lambda_{\mathrm{d}}/\rho_{\mathrm{d}} c_{\mathrm{p,d}} \tag{S4a}$$

$$e_{\mathrm{d}} = \sqrt{\Lambda_{\mathrm{d}} \rho_{\mathrm{d}} c_{\mathrm{p,d}}} \tag{S4b}$$

with the following properties of glycerol-water estimated using the properties of the pure components: thermal conductivity $\Lambda_{\mathrm{d}}$, density $\rho_{\mathrm{d}}$, and isobaric heat capacity $c_{\mathrm{p,d}}$. $\Lambda_{\mathrm{d}}$ is estimated by a linear interpolation based on volume fractions:

$$\Lambda_{\mathrm{d}} = \varphi_{\mathrm{g}} \Lambda_{\mathrm{g}} + (1 - \varphi_{\mathrm{g}}) \Lambda_{\mathrm{w}} \tag{S4c}$$

with the thermal conductivity of pure glycerol[8] and water[9] respectively as

$$\Lambda_{\mathrm{g}} \approx 0.2868\ \mathrm{W\,m^{-1}\,K^{-1}} \tag{S4d}$$

$$\Lambda_{\mathrm{w}} \approx 0.5606\ \mathrm{W\,m^{-1}\,K^{-1}} \tag{S4e}$$

$\rho_{\mathrm{d}}$ is estimated from ideal solution assumption, defined from glycerol mole fraction $x_{\mathrm{g}}$:

$$\rho_{\mathrm{d}} = \frac{x_{\mathrm{g}}M_{\mathrm{g}} + (1 - x_{\mathrm{g}})M_{\mathrm{w}}}{\frac{x_{\mathrm{g}}M_{\mathrm{g}}}{\rho_{\mathrm{g}}} + \frac{(1 - x_{\mathrm{g}})M_{\mathrm{w}}}{\rho_{\mathrm{w}}}} \tag{S4f}$$

where $M_{\mathrm{g}}$ and $M_{\mathrm{w}}$ are the molar masses of glycerol and water, respectively. $\rho_{\mathrm{g}}$ and $\rho_{\mathrm{w}}$ are defined in equation (S2). $c_{\mathrm{p,d}}$ is estimated by a linear interpolation based on mass fractions:

$$c_{\mathrm{p,d}} = w_{\mathrm{g}}c_{\mathrm{p,g}} + (1 - w_{\mathrm{g}})c_{\mathrm{p,w}} \tag{S4g}$$

with the isobaric heat capacity of pure glycerol[10] and water[11] as:

$$c_{\mathrm{p,g}} \approx 2377\ \mathrm{J\ kg^{-1}\ K^{-1}} \tag{S4h}$$

$$c_{\mathrm{p,w}} \approx 4490\ \mathrm{J\ kg^{-1}\ K^{-1}} \tag{S4i}$$

## Supplementary Section 3. Convective Flow in the Unfrozen Droplet

Vaporization cooling at the droplet surface leads to an increased local glycerol concentration and a decreased local temperature. This induces an internal circulating flow within the droplet, which was visualized using micro-particle image velocimetry (MicroPIV), as shown in Figure S2A-B. The internal circulating flow gradually stops as crystallization proceeds due to increased solidification (see Movie S5).

The experimental method of MicroPIV is as follows. Microparticles based on polystyrene (10 µm in size, Product #72986, Sigma-Aldrich) were embedded into a glycerol-water droplet ($x_{\mathrm{g,0}}$ = 2.40 mol%). Illumination was optimized to enable direct observation of the microparticles via the high-speed camera. All other experimental protocols remained consistent with those described in the ‘Experimental Process’ section in Materials and Methods. PIV analysis was performed in PIVlab[12] within the MATLAB environment.

The geometry of the observed asymmetric circulating flow resembles buoyancy-driven (Rayleigh-Bénard) convection reported in quasi-spherical evaporating droplets on heated nonwetting substrates under atmospheric pressure[13-15]. However, in our experiments, the Péclet numbers for heat $\mathrm{Pe}_{\mathrm{thermal}} \approx 289$ and mass diffusion $\mathrm{Pe}_{\mathrm{mass}} \approx 4.13 \times 10^5$ significantly exceed

unity, indicating that advection dominates over diffusion, in contrast to the diffusion-dominated regime in atmospheric droplets[13].

The Reynolds number $\mathrm{Re} = 3.69$ suggests that both viscous and inertial forces are significant. A detailed force balance for this buoyancy-driven flow is derived in Supplementary Section 4. Using an average peripheral flow velocity of $u \approx 27.3\ \mathrm{mm\ s^{-1}}$ from MicroPIV measurements (Figure S2B), the force balance estimates a density difference of $\Delta\rho_\mathrm{d} \approx 76.3\ \mathrm{kg\ m^{-3}}$ and a diffusion mass flux $J_\mathrm{dif} \sim 0.0148\ \mathrm{kg\ m^{-2}s^{-1}}$ (see Supplementary Section 4 for details). Notably, the magnitude of $J_\mathrm{dif}$ matches the experimental vaporization mass flux of $J_\mathrm{vap} = 0.0128\ \mathrm{kg\ m^{-2}s^{-1}}$ (see Supplementary Section 2), confirming the model's validity.

To assess the potential contribution of surface tension-driven (Marangoni) flow, we analyzed the temperature contrast on the droplet surface using infrared imaging (see Figure S2C-D). The environmental thermal radiation and reflections in the chamber limit the precision of absolute temperature measurements[16]. Therefore, infrared imaging was used solely to evaluate surface temperature homogeneity. As shown in Figure S2D, initially, rapid vaporization cooling reduces surface temperature homogeneity (before $t \approx t_\mathrm{n} - 7$ s). As convective flow strengthens with time (see Movie S5), enhanced mixing improves the homogeneity (from $t \approx t_\mathrm{n} - 6$ s). In the final ~5 s prior to nucleation, surface temperature within the confidence region remains nearly uniform, suggesting a marginal role for surface tension gradients during this period.

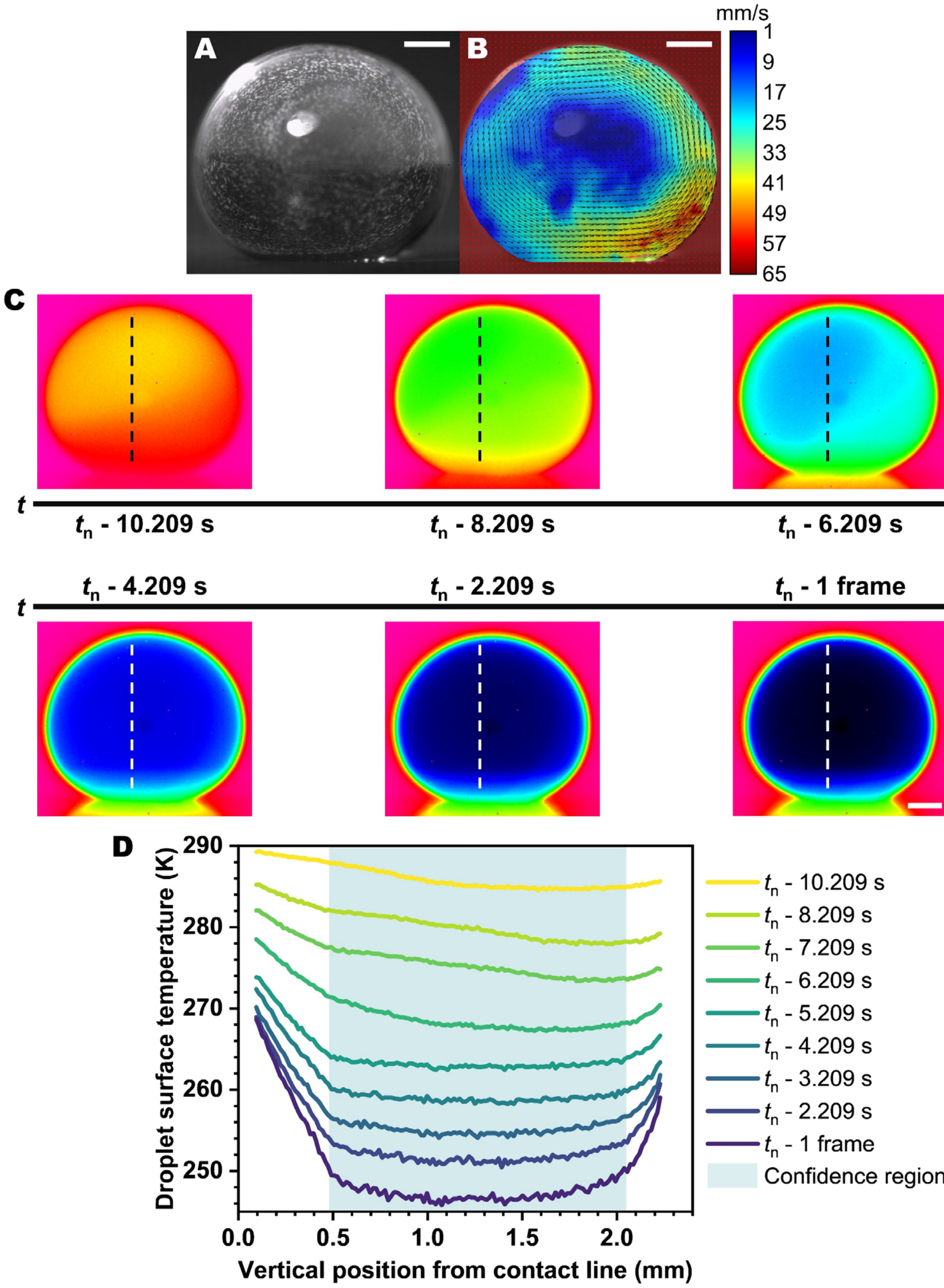


Figure S2. Characterization of the convective flow in the unfrozen droplet under reduced pressure, for a glycerol-water droplet with 10 µL initial volume and $x_{g,0}$ = 2.40 mol%. **(A)** Side-view high-speed image of the asymmetric circulating flow prior to nucleation, visualized by polystyrene microparticles (10 µm) embedded in the droplet (see Movie S5). **(B)** Velocity contours and vector plots for the same circulating flow in panel **(A)**. **(C)** Infrared images of the surface of a glycerol-water droplet with 10 µL initial volume and $x_{g,0}$ = 2.40 mol% at various times $t$ relative to the moment of nucleation $t_n$, with the last frame in the sequence corresponding

to the frame immediately preceding visible nucleation in infrared imaging. The infrared images were captured at 115 frames per second (see 'Experimental Process' in Materials and Methods). The dashed line indicates the positions of the temperature profile analyzed in panel **(D)**, offset slightly from the center to avoid measurement errors (in a central circular region visible in the infrared images, such as the third frame). **(D)** Droplet surface temperature profiles along vertical positions from contact line at various $t$ (relative to $t_n$). From Lambert's cosine law[17], the intensity of emitted radiation decreases at larger angles of incidence (near the outer boundary of the side-view droplet), leading to larger measurement errors. Thus, an empirically determined confidence region is denoted by the light blue shaded area. The scale bars represent 0.5 mm in panels **(A)**, **(B)**, and **(C)**.

## Supplementary Section 4. Modeling the Convective Flow in the Unfrozen Droplet

Micro-particle image velocimetry (MicroPIV) in Supplementary Section 3 reveals that fluid particles within the evaporating droplet move in radially parallel directions with minimal disruption, indicative of laminar flow. The Reynolds number (Re) for the most developed convective cell (prior to nucleation) is approximated: Re $\approx \rho_{\mathrm{d}} u R_d / \mu_{\mathrm{d}} = 3.69$, where $\rho_{\mathrm{d}}$ is the density of glycerol-water (defined in Supplementary Section 2), $u$ is the characteristic flow velocity estimated by the average peripheral flow velocity and $\mu_{\mathrm{d}}$ is the dynamic viscosity corresponding to the average droplet concentration. Assuming fluid incompressibility, $\mu_{\mathrm{d}}$ (in Pa·s) as a function of glycerol mass fraction $w_{\mathrm{g}}$ (dimensionless) and bulk droplet temperature $T_{\mathrm{d,bulk}}$ (in K) is expressed using an empirical relation[18]:

$$\mu_{\mathrm{d}} = \mu_{\mathrm{d}}^{0} \exp\left\{\left[28.75 - \ln\left(1000\mu_{\mathrm{d}}^{0}\right)\right]\left(\frac{T_{\mathrm{gt}}}{T_{\mathrm{d}}}\right)^{\beta}\right\} \qquad (S5a)$$

where $\mu_{\mathrm{d}}^{0}$ and $\beta$ are fitting parameters, $T_{\mathrm{gt}}$ is the glass transition temperature for a glycerol-water solution with glycerol mass fraction $w_{\mathrm{g}}$. These parameters are specified as[18]:

$$\mu_{\mathrm{d}}^{0} = \exp(-2.1686 + 2.7096 w_{\mathrm{g}} - 1.4449 {w_{\mathrm{g}}}^{2})/1000 \qquad (S5b)$$

$$\beta = 3.6344 + 1.2198 w_{\mathrm{g}} - 1.6142 {w_{\mathrm{g}}}^{2} \qquad (S5c)$$

$$T_{gt} = \frac{195.5w_g + 276.51(1 - w_g)}{w_g + 1.95(1 - w_g)} \tag{S5d}$$

To assess the relative contributions of advective and diffusive transport, Péclet numbers for heat ($\mathrm{Pe_{thermal}} = uR_d/\alpha_d \approx 289$) and mass diffusion ($\mathrm{Pe_{mass}} = uR_d/D_{mass} \approx 4.13 \times 10^5$) are evaluated. The thermal diffusivity of glycerol-water $\alpha_d$ is defined in Supplementary Section 2. The mass diffusion coefficient $D_{mass}$ (in $m^2\ s^{-1}$) at bulk droplet temperature $T_{d,bulk}$ is determined using an empirical relation with glycerol mole fraction $x_g$ for glycerol-water solution at 298.15 K[19] and the temperature dependence from the Stokes–Einstein equation:

$$D_{mass} = \frac{T_{d,bulk}}{298.15} \frac{\mu_d(x_g, 298.15\ \mathrm{K})}{\mu_d(x_g, T_{d,bulk})} \frac{1.024 - 0.91x_g}{1 + 7.5x_g} \times 10^{-9} \tag{S6}$$

As a result, the calculated $\mathrm{Pe_{thermal}}$ and $\mathrm{Pe_{mass}}$ both significantly exceed unity, confirming that advective transport dominates heat and mass transfer within the droplet.

Reynolds number of $\mathrm{Re} \approx 3.69$ suggests that both viscous and inertial forces are significant. Therefore, the momentum balance in the convective cell is derived from the Navier-Stokes equation for an incompressible fluid with buoyancy, under steady-state and Boussinesq approximations:

$$\rho(\mathbf{u} \cdot \nabla)\mathbf{u} = -\nabla p + \mu \nabla^2 \mathbf{u} + \Delta\rho \mathbf{g} \tag{S7}$$

where $\rho$ is the fluid density, $p$ is the pressure, $\mathbf{u}$ is the velocity field, and $\mathbf{g}$ is the gravitational acceleration. Scaling each term using characteristic values of droplet-average density $\rho_d$, equivalent droplet radius $R_d$, average peripheral flow velocity $u$, and standard gravitational acceleration $g$, and noting that the pressure term $\nabla p$ integrates to zero over the droplet volume, the force balance over the entire droplet volume is approximated as:

$$\Delta\rho_d g \cdot \frac{4}{3}\pi {R_d}^3 \approx \mu_d \frac{u}{{R_d}^2} \cdot \frac{4}{3}\pi {R_d}^3 + \rho_d \frac{u^2}{R_d} \cdot \frac{4}{3}\pi {R_d}^3 \tag{S8}$$

Thus, the global force balance simplifies to:

$$\Delta\rho_d g {R_d}^3 \approx \mu_d u R_d + \rho_d u^2 {R_d}^2 = \mu_d u R_d (1 + \mathrm{Re}) \tag{S9}$$

This yields a density difference $\Delta\rho_\mathrm{d} \approx 76.3$ kg m$^{-3}$ for the convective flow of $u \approx 27.3$ mm s$^{-1}$ discussed in the main text. The temperature effect on density is negligible, given the volume thermal expansion coefficient of $\alpha_\mathrm{expan} \approx 2.90 \times 10^{-4}$ K$^{-1}$ for 2.84 mol% glycerol-water at 298.15 K[20]. Therefore, the density difference is attributed primarily to water loss.

Given the large $\mathrm{Pe}_\mathrm{mass}$, consider a thin boundary layer near the droplet surface. Within this thin layer, mass transport perpendicular to the surface is primarily by diffusion, while transport parallel to the surface is primarily by convection. The following simplifying approximations are made:

1. Glycerol evaporation is negligible due to its low vapor pressure[4].
2. The unfrozen droplet constitutes an ideal solution of Newtonian, incompressible fluid.
3. Droplet volume is quasi-stationary. Droplet temperature is quasi-stationary and spatially uniform.
4. The driving difference of mass concentration is quasi-stationary. All mass concentration differences are concentrated in the boundary layer.
5. Bulk droplet properties (i.e., outside the boundary layer) are uniform and equal to the droplet-average properties including density $\rho_\mathrm{d}$, water mass fraction $w_\mathrm{w}$, water mass concentration $c_\mathrm{w}$, while the corresponding uniform properties on the droplet surface are $\rho_\mathrm{d} + \Delta\rho_\mathrm{d}$, $w_\mathrm{w} + \Delta w_\mathrm{w}$, $c_\mathrm{w} + \Delta c_\mathrm{w}$, respectively.

Start with the definition of water mass concentration difference $\Delta c_\mathrm{w}$:

$$\Delta c_\mathrm{w} = w_\mathrm{w}\rho_\mathrm{d} - (w_\mathrm{w} + \Delta w_\mathrm{w})(\rho_\mathrm{d} + \Delta\rho_\mathrm{d}) = -w_\mathrm{w}\Delta\rho_\mathrm{d} - \Delta w_\mathrm{w}\rho_\mathrm{d} - \Delta w_\mathrm{w}\Delta\rho_\mathrm{d} \tag{S10}$$

where the term $\Delta w_\mathrm{w}\Delta\rho_\mathrm{d}$ is second-order small and can be neglected. $\rho_\mathrm{d}$ is approximated as a linear function of $w_\mathrm{w}$:

$$\Delta c_\mathrm{w} \approx -w_\mathrm{w}\Delta\rho_\mathrm{d} - \frac{\rho_\mathrm{d}\Delta\rho_\mathrm{d}}{d\rho_\mathrm{d}/dw_\mathrm{w}} \tag{S11}$$

For an ideal glycerol-water solution, $\rho_\mathrm{d}$ is given by the volume additivity rule:

$$\frac{1}{\rho_\mathrm{d}} = \frac{w_\mathrm{w}}{\rho_\mathrm{w}} + \frac{1 - w_\mathrm{w}}{\rho_\mathrm{g}} \tag{S12}$$

where $\rho_{\mathrm{w}}$ and $\rho_{\mathrm{g}}$ are the densities of pure water and glycerol, respectively (see Supplementary Section 2). Differentiating $\rho_{\mathrm{d}}$ with respect to $w_{\mathrm{w}}$:

$$\frac{d}{dw_{\mathrm{w}}}\left(\frac{1}{\rho_{\mathrm{d}}}\right) = \frac{1}{\rho_{\mathrm{w}}} - \frac{1}{\rho_{\mathrm{g}}} \tag{S13}$$

Thus:

$$\frac{d\rho_{\mathrm{d}}}{dw_{\mathrm{w}}} = -{\rho_{\mathrm{d}}}^2\left(\frac{1}{\rho_{\mathrm{w}}} - \frac{1}{\rho_{\mathrm{g}}}\right) \tag{S14}$$

Substitute into the expression for $\Delta c_{\mathrm{w}}$:

$$\Delta c_{\mathrm{w}} \approx \Delta\rho_{\mathrm{d}}\left[\frac{\rho_{\mathrm{w}}\rho_{\mathrm{g}}}{\rho_{\mathrm{d}}(\rho_{\mathrm{g}} - \rho_{\mathrm{w}})} - w_{\mathrm{w}}\right] \tag{S15}$$

The convective flow brings fluid elements from the bulk droplet (higher water mass concentration) to the surface (lower water mass concentration due to vaporization). These elements are exposed to the surface for a characteristic time $\tau_{\mathrm{dif}}$, during which water diffuses out of the element. After this time, the element is mixed back into the bulk droplet. Therefore, $\tau_{\mathrm{dif}}$ can be scaled as $\tau_{\mathrm{dif}} \sim R_{\mathrm{d}}/u$. Considering the short $\tau_{\mathrm{dif}} \sim 47.7$ ms, water only diffuses a small distance out of the fluid element. For this short penetration depth, the fluid element effectively behaves like a semi-infinite medium in the direction normal to the diffusion interface. Therefore, Higbie's penetration theory[21] is applied, where the thickness of the boundary layer $\delta_{\mathrm{dif}}$ scales as:

$$\delta_{\mathrm{dif}} \sim \sqrt{D_{\mathrm{mass}} \cdot \tau_{\mathrm{dif}}} \sim \sqrt{D_{\mathrm{mass}} \cdot \frac{R_{\mathrm{d}}}{u}} = R_{\mathrm{d}}\sqrt{\frac{D_{\mathrm{mass}}}{uR_{\mathrm{d}}}} = \frac{R_{\mathrm{d}}}{\sqrt{\mathrm{Pe}_{\mathrm{mass}}}} \tag{S16}$$

Consequently, the diffusion mass flux $J_{\mathrm{dif}}$ is derived:

$$J_{\mathrm{dif}} \approx D_{\mathrm{mass}}\frac{\Delta c_{\mathrm{w}}}{\delta_{\mathrm{dif}}} \sim D_{\mathrm{mass}}\frac{\Delta\rho_{\mathrm{d}}\sqrt{\mathrm{Pe}_{\mathrm{mass}}}}{R_{\mathrm{d}}}\left[\frac{\rho_{\mathrm{w}}\rho_{\mathrm{g}}}{\rho_{\mathrm{d}}(\rho_{\mathrm{g}} - \rho_{\mathrm{w}})} - w_{\mathrm{w}}\right] \tag{S17}$$

## Supplementary Section 5. Molecular Dynamics (MD) Simulation

All molecular dynamics simulations were conducted using the Large-scale Atomic/Molecular Massively Parallel Simulator (LAMMPS) software[22]. The all-atom TIP4P/Ice water model[23] was employed, as it offers highly accurate predictions of water's melting point and captures hydrogen bond behavior with greater precision compared to coarse-grained models such as the monatomic water (mW) model[24]. A time step of 1 fs was used to integrate the equations of motion. The temperature was regulated by the stochastic velocity rescaling thermostat with a 0.1 ps relaxation time[25], and pressure was maintained using the Parrinello-Rahman barostat with a 1 ps relaxation time[26]. The SHAKE algorithm was implemented to constrain bond lengths and angles. A cutoff distance of 0.85 nm was applied for Lennard-Jones and short-range Coulomb interactions. Long-range Coulomb forces were computed using the particle-particle particle-mesh (PPPM) solver with an accuracy of $1.0 \times 10^{-4}$. Tail corrections were applied to the pressure and energy to account for long-range Lennard-Jones interactions beyond the cutoff.

A modified glycerol force field[27-30] was used to describe the atomic interactions within the glycerol molecule. The total potential energy function, $E_{\mathrm{total}}$, is defined as the sum of the following five terms:

$$
\begin{cases}
E_{\mathrm{total}} = E_{\mathrm{bonds}} + E_{\mathrm{angles}} + E_{\mathrm{torsions}} + E_{\mathrm{Lennard-Jones}} + E_{\mathrm{Coulomb}} & (\mathrm{S18}a) \\
E_{\mathrm{bonds}} = K_{\mathrm{b}}(r - r_0)^2 & (\mathrm{S18}b) \\
E_{\mathrm{angles}} = K_{\alpha}(\alpha - \alpha_0)^2 & (\mathrm{S18}c) \\
E_{\mathrm{torsions}} = K_{\Phi}(1 + \cos(n\Phi))^2 & (\mathrm{S18}d) \\
E_{\mathrm{Lennard-Jones}} = 4\sqrt{\varepsilon_{\mathrm{i}}\varepsilon_{\mathrm{j}}}\left(\left(\dfrac{\sigma_{\mathrm{i}} + \sigma_{\mathrm{j}}}{2r_{\mathrm{ij}}}\right)^{12} - \left(\dfrac{\sigma_{\mathrm{i}} + \sigma_{\mathrm{j}}}{2r_{\mathrm{ij}}}\right)^{6}\right) & (\mathrm{S18}e) \\
E_{\mathrm{Coulomb}} = \dfrac{q_{\mathrm{i}}q_{\mathrm{j}}e^2}{r_{\mathrm{ij}}} & (\mathrm{S18}f)
\end{cases}
$$

where $E_{\mathrm{bonds}}$ is the bond energy, $K_{\mathrm{b}}$ is the force constant of the bond, $r$ is the actual bond length, and $r_0$ is the equilibrium bond length; $E_{\mathrm{angles}}$ is angle energy, $K_{\alpha}$ is the force constant of the angle, $\alpha$ is the actual angle, and $\alpha_0$ is the equilibrium angle; $E_{\mathrm{torsions}}$ is the torsional rotation barriers energy, $K_{\Phi}$ is the force constant of the torsion, $n$ is the multiplicity, and $\Phi$ is the torsional angle; $E_{\mathrm{Lennard-Jones}}$ is the van der Waals interactions, $\varepsilon_{\mathrm{ij}}$ and $\sigma_{\mathrm{ij}}$ are Lennard-Jones parameters for atoms i and j, and $r_{\mathrm{ij}}$ is the interatomic distance; and $E_{\mathrm{Coulomb}}$ is the electrostatic energy, $q_{\mathrm{i}}$ and $q_{\mathrm{j}}$ are the

charges of the atoms, and $e$ is the elementary charge. The detailed parameters are listed in Table S3.

Initial configurations of the glycerol-water solution and hexagonal ice (Ice Ih, which served as the seed to initiate freezing) were generated in MATLAB according to the Bernard-Fowler ice rules[31]. The initial coexistence system contained 1728 water molecules and 60 glycerol molecules, yielding a representative bulk concentration of ~3.3 mol%. This concentration was selected to approximate the pre-nucleation glycerol mole fraction (~2.9 mol%, as derived in Supplementary Section 2) of the primary reported droplet (initial glycerol fraction $x_{g,0}$ = 2.40 mol%), allowing us to qualitatively model the fundamental molecular mechanisms of freeze-concentration at low concentrations. The entire simulation box was equilibrated under anisotropic barostat conditions with periodic boundary conditions at 250 K and 0 atm for 2 ns. Next, a 2-ns simulation was performed. During this simulation, the motion of Ice Ih molecules on one side of the box was frozen, while the glycerol-water molecules on the other side were melted at 400 K using an NPzT ensemble (with a fluctuating box dimension only in the z-direction and constant area in the xy-directions). Finally, the entire system was relaxed at 250 K and 0 atm for 1 ns. The equilibrated box dimensions are 17.72 Å × 47.93 Å × 72.30 Å (X × Y × Z). Data for the 1 μs ice growth simulation were sampled every 50 ns.

To analyze the freezing process, the CHILL+ algorithm[32] was used to distinguish ice-like and liquid-like water molecules based on oxygen atom positions. In this study, only the positions of the oxygen atoms in water molecules were considered for identifying different water crystal structures. Molecules identified as hexagonal ice (numbers of which molecules denoted as $n_{i,h}$), cubic ice ($n_{i,c}$), and interfacial ice ($n_{i,i}$) were classified as solid (Figure S3A), while all others were classified as liquid water ($n_w$). We calculated the system ice mole fraction ($x_{i,total}$) and liquid-phase glycerol mole fraction ($x_g$) by

$$x_{\mathrm{i,total}} = \frac{n_{\mathrm{i,h}} + n_{\mathrm{i,c}} + n_{\mathrm{i,i}}}{n_{\mathrm{total}}} \tag{S19}$$

$$x_{\mathrm{g}} = \frac{n_{\mathrm{g}}}{n_{\mathrm{g}} + n_{\mathrm{w}}} \tag{S20}$$

where $n_{total}$ is the total number of molecules in the system and $n_g$ is the number of glycerol molecules. The simulated crystallization process results in a stacking-disordered structure of hexagonal ice and cubic ice, consistent with existing experimental and simulation results of ice crystallized from supercooled water[33-39] (Figure S3B-C). Specifically, to investigate the mole fraction of cubic ice in stacking-disordered ice, we define the cubicity ($C$*)[40] as:

$$C^* = \frac{n_{\mathrm{i,c}}}{n_{\mathrm{i,h}} + n_{\mathrm{i,c}}} \tag{S21}$$

It should be noted that $C$* is applicable for characterizing ice polymorphs but is meaningless in the liquid phase. During the first 200 ns, a large amount of disordered cubic ice forms, and $C$* rapidly increases to ~48% (Figure S3D) as the system's potential energy decreases (Figure S3E), indicating kinetically dominated ice growth. Due to the constant-temperature condition in the simulation, the system cannot overcome the nucleation barrier of hexagonal ice, and $C$* remains at a high level.

As crystallization progressed, the dispersed glycerol molecules are rejected by the growing ice and gradually aggregate in the remaining liquid, forming a freeze-concentrated solution and a distinct solid-liquid interface. This aggregation is consistent with reported nanosegregation in dilute glycerol-water solutions at low temperatures, which is driven by the higher diffusion coefficient of water compared to glycerol as temperature decreases[41].

The MD conditions map to the experiments only at the level of a local ice-solution interfacial region. The simulated initial glycerol mole fraction of ~3.3 mol% is close to the estimated pre-nucleation droplet-average value of ~2.9 mol% for the primary droplet. The fixed simulation temperature of 250 K is comparable to the measured mean nucleation temperature of ~250 K. However, it was selected to obtain ice growth within the accessible MD time and does not reproduce the full non-isothermal trajectory through recalescence. The difference between the imposed condensed-phase barostat pressure (0 atm) and the experimental chamber pressure (~50 Pa during droplet freezing) is negligible relative to condensed-phase intermolecular stress scales. This pressure comparison does not imply that the experimental vapor environment is reproduced: the periodic simulation contains no free surface and therefore does not model vaporization, gap flow, or droplet motion.

Additional limitations arise from finite system size and force-field selection. The 1788-molecule periodic cell represents a localized solid-liquid interfacial region, so it cannot capture macroscopic solute redistribution, open-boundary heat and mass transfer, or mechanical stress accumulation. TIP4P/Ice and the modified glycerol force field reproduce the intended molecular interactions but can shift diffusion rates, phase-equilibrium conditions, and the quantitative partitioning of glycerol. The thermostat removes the released latent heat, and the barostat controls the volume response, in ways that differ from the experimental open, non-isothermal process. Accordingly, the simulated growth rate, final ice fraction, and ~4% density change (Figure 3D) are not used as quantitative experimental predictions. The robust result used in this work is the qualitative observation that the advancing ice rejects glycerol and retains a freeze-concentrated liquid region.

Table S3. Bond, angle, torsional, and L-J parameters, and atomic charges of the glycerol force field.

| **Bond** | **$K_b$ [kcal mol$^{-1}$ Å$^{-2}$]** | **$r_0$ [Å]** |
|---|---|---|
| CC | 310.0 | 1.526 |
| CO | 320.0 | 1.410 |
| CH | 309.0 | 1.090 |
| OH | 545.0 | 0.960 |
| **Angle** | **$K_\alpha$ [kcal mol$^{-1}$ rad$^{-2}$]** | **$\alpha_0$ [deg]** |
| CCC | 44.0 | 109.5 |
| CCO | 50.0 | 109.5 |
| CCH | 50.0 | 109.5 |
| COH | 55.0 | 109.5 |
| HCH | 35.0 | 109.5 |
| OCH | 50.0 | 109.5 |
| **Torsion** | **$K_\Phi$ [kcal mol$^{-1}$]** | ***n*** |
| CCCH | 0.1556 | 3 |
| CCCO | 0.1556 | 3 |
| OCCO | 0.1440 | 3 |
| OCCO | 1.0000 | 2 |
| OCCH | 0.1556 | 3 |
| HOCC | 0.1667 | 3 |
| HOCH | 0.1667 | 3 |
| HCCH | 0.1556 | 3 |
| **Lennard-Jones** | **$\sigma$ [Å]** | **$\varepsilon$ [kcal mol$^{-1}$]** |
| C | 3.816 | 0.1094 |
| O | 2.8508 | 0.1591 |
| Aliphatic H | 2.774 | 0.0157 |
| Hydroxyl H | 1.4254 | 0.0498 |
| **Atomic charges** | **$q$ [electronic units]** | |
| Terminal C | 0.182 | |
| Central C | 0.055 | |
| Terminal aliphatic H | 0.026 | |
| Central aliphatic H | 0.044 | |
| Hydroxyl H | 0.4158 | |
| Terminal O | -0.6048 | |
| Central O | -0.6048 | |

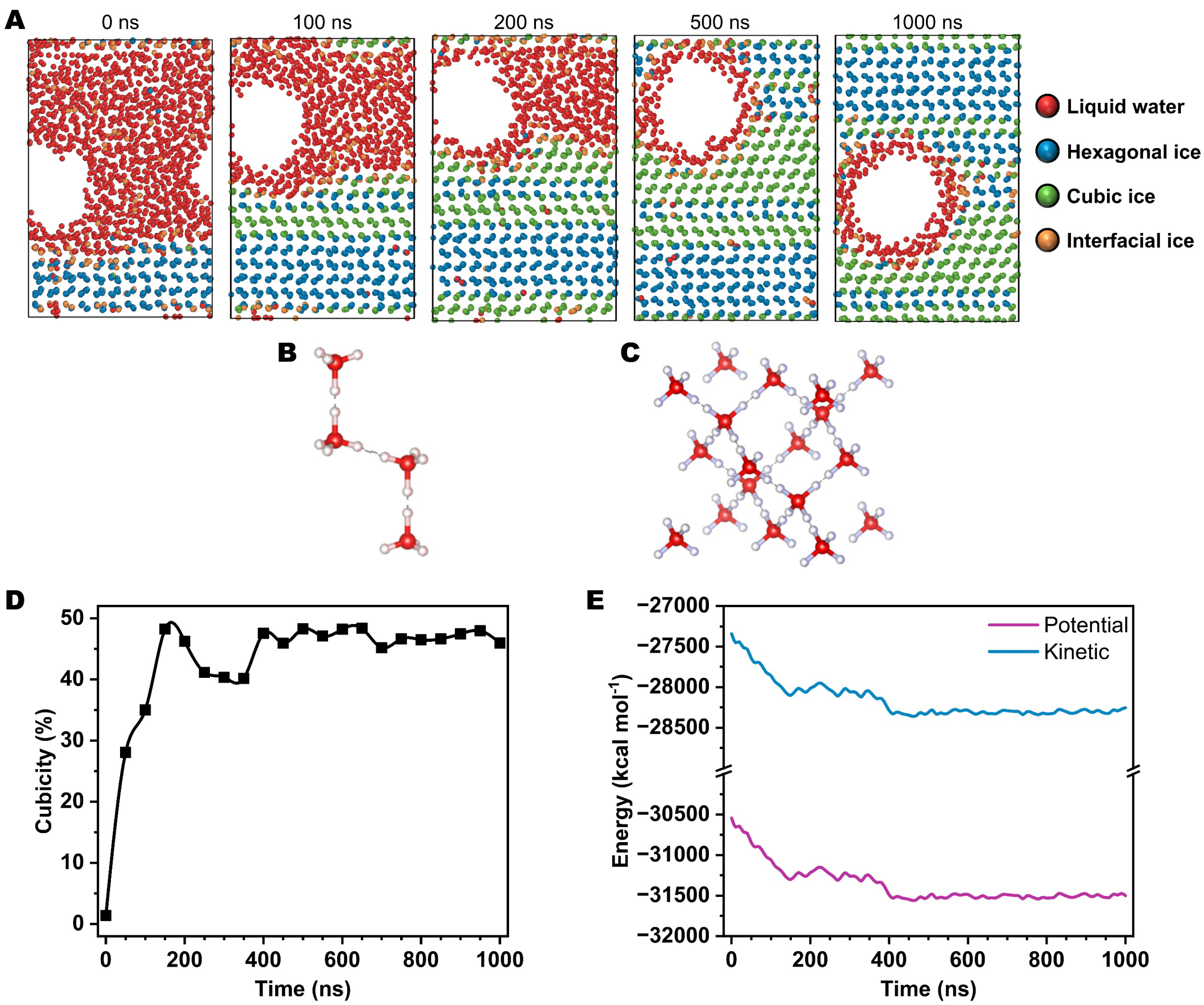


Figure S3. Analysis of ice structure and energetics from MD simulation. **(A)** Structural evolution of water molecules during the freezing process, classified based on the positions of the $H_2O$ oxygen atoms. **(B)** Lattice structure of hexagonal ice (Ih). **(C)** Lattice structure of cubic ice (Ic). **(D)** Temporal evolution of cubicity *C**. **(E)** Temporal evolution of the system's potential and kinetic energy.

## Supplementary Section 6. Vaporization Force and Self-Propulsion

In this section, we discuss the driving force for the droplet self-propulsion during recalescence and the role of glycerol addition in suppressing it. During recalescence, the rapid formation of ice creates distinct regions within the droplet: a "freezing" region containing growing ice

dendrites and a "supercooled" liquid region. These localized differences manifest as asymmetries in vapor pressure $P_v$ and vaporization mass flux $J_{vap}$ on the droplet surface, driving self-propulsion of the droplet[16, 42-45]. Assuming negligible evaporation of glycerol (due to its low vapor pressure[4]) and the minimal effects from non-condensable gases, the $J_{vap}$ of water from the droplet surface at temperature $T$ is calculated using the Hertz-Knudsen-Schrage equation:

$$J_{\text{vap}} = \frac{2\sigma}{2-\sigma}\sqrt{\frac{M_{\text{w}}}{2\pi R}}\left(\frac{P_{\text{v}}}{\sqrt{T}} - \frac{P_\infty}{\sqrt{T_\infty}}\right) \tag{S22}$$

where the accommodation coefficient $\sigma = 0.457$ is empirically derived from the experimental vaporization mass flux of the $x_{g,0} = 2.40$ mol% droplet (see Supplementary Section 2), $M_w$ is the molar mass of water, $R$ is the universal gas constant, $P_\infty$ is the chamber pressure, and $T_\infty \approx 295$ K is the vapor temperature, approximated as room temperature. See equation (S33) for definitions of $P_v$ for glycerol-water. It is important to note that $\sigma$ inherently carries uncertainty and is sensitive to dynamic variations in local interfacial conditions under simultaneous freeze-concentration and vaporization. However, applying a constant $\sigma$ across the tested parameters serves our objective of establishing a qualitative framework to elucidate self-propulsion suppression, rather than functioning as a precise quantitative predictor.

To quantify the asymmetric vaporization, we first estimate the local ice mass fraction $\widetilde{w}_{\text{i}}$ in the freezing region. By assuming the rapid recalescence process (tens to hundreds of milliseconds) as adiabatic, we can balance the latent heat released by solidification with the change in internal energy of the mixture, the local ice mass fraction in the freezing region is estimated by balancing the latent heat release with the internal energy change of the mixture[16, 46, 47]:

$$\widetilde{w}_{\text{i}} L_{\text{solid}} = \begin{cases} \widetilde{w}_{\text{i}} c_{\text{p,i}}\,(T_{\text{f}} - T_{\text{n}}) + (1 - \widetilde{w}_{\text{i}}\,) c_{\text{p,w}} (T_{\text{f}} - T_{\text{n}}) & \text{, for pure water} \\ \widetilde{w}_{\text{i}} c_{\text{p,i}} (T_{\text{f}} - T_{\text{n}}) + (1 - \widetilde{w}_{\text{i}}) c_{\text{p,d}} (T_{\text{f}} - T_{\text{n}}) & \text{, for glycerol-water} \end{cases} \tag{S23}$$

Here, $T_f$ is the equilibrium freezing temperature, $T_n$ is the nucleation temperature, $L_{\text{solid}} \approx 3.34 \times 10^5$ J kg$^{-1}$ is the latent heat of solidification of water, $c_{\text{p,i}} \approx 2093$ J kg$^{-1}$ K$^{-1}$ is the isobaric heat capacity of ice, and the isobaric heat capacities of liquid water $c_{\text{p,w}}$ and glycerol-water solution $c_{\text{p,d}}$ are detailed in Supplementary Section 2. $T_{\text{f}}$ (in K) is related to glycerol mass fraction $w_g$ and is estimated based on pre-nucleation droplet-average concentrations (see

Supplementary Section 2 for characterizations of pre-nucleation properties). For glycerol concentrations before the eutectic point, (i.e., $w_g \leq 0.65$), an empirical relation for $T_f$ is derived from reported experimental data[48]:

$$T_{\mathrm{f}} = -5.0794\exp\left(w_{\mathrm{g}}/0.28972\right) + 278.37 \tag{S24}$$

The results of $T_f$ are shown in Figure S4A. Consequently, the local ice mass fraction $\widetilde{w}_{\mathrm{i}}$ in the freezing region during recalescence is:

$$\widetilde{w}_{\mathrm{i}} = \begin{cases} \dfrac{c_{\mathrm{p,w}}}{L_{\mathrm{solid}}/(T_{\mathrm{f}} - T_{\mathrm{n}}) - c_{\mathrm{p,i}} + c_{\mathrm{p,w}}} & \text{, for pure water} \\ \dfrac{c_{\mathrm{p,d}}}{L_{\mathrm{solid}}/(T_{\mathrm{f}} - T_{\mathrm{n}}) - c_{\mathrm{p,i}} + c_{\mathrm{p,d}}} & \text{, for glycerol-water} \end{cases} \tag{S25}$$

The local glycerol mole fraction $\tilde{x}_{\mathrm{g}}$ in the liquid phase of the freezing region during recalescence is then:

$$\tilde{x}_{\mathrm{g}} = \frac{w_{\mathrm{g}}/M_{\mathrm{g}}}{w_{\mathrm{g}}/M_{\mathrm{g}} + \left(1 - w_{\mathrm{g}} - \widetilde{w}_{\mathrm{i}}\right)/M_{\mathrm{w}}} \text{, for glycerol-water} \tag{S26}$$

To estimate the maximum possible driving force for self-propulsion, the droplet surface during recalescence is assumed as two quasi-hemispheres: a "supercooled" region at its pre-nucleation state and a "freezing" region influenced by the advancing ice front (Figure 2A). The following assumptions are made:

1. Initial temperature and composition gradients within the droplet prior to nucleation are negligible.
2. Droplet deformation is negligible due to the small local ice mass fraction during recalescence ($\widetilde{w}_{\mathrm{i}} \approx 18.9\% - 26.8\%$).
3. Heat and mass exchange with the surroundings are negligible over the short recalescence timescale.
4. Internal convective flow in the droplet, which gradually stops with solidification, is not considered (see Supplementary Section 3).

The recalescence timescale is significantly smaller than the characteristic thermal diffusion time $\tau_{\mathrm{thermal}} = {R_{\mathrm{d}}}^2/\alpha_{\mathrm{thermal}} \sim 10^1$ s and mass diffusion time $\tau_{\mathrm{mass}} = {R_{\mathrm{d}}}^2/D_{\mathrm{mass}} \sim 10^3$ s, with

$\alpha_{\mathrm{thermal}}$ and $D_{\mathrm{mass}}$ detailed in Supplementary Section 2 and 4, respectively. Consequently, heat transfer within the droplet is dominated by latent heat release during recalescence, rather than thermal diffusion. Similarly, local concentration gradients are dominated by the rapid growth of ice dendrites, rather than mass diffusion.

Based on these considerations, we approximate the supercooled region with temperature $T_{\mathrm{n}}$ with pre-nucleation composition. The freezing region is approximated with $T_{\mathrm{f}}$. For a pure water droplet, this surface is approximated as pure ice; for a glycerol-water droplet, considering its freeze-concentrated structure (see main text), it is approximated as a solution with local glycerol mole fraction $\tilde{x}_{\mathrm{g}}$. Notably, the approximated compositions have minimal impact on the recoil pressure $P_{\mathrm{r}}$ (defined below) compared to temperature (Figure S4B).

The recoil pressure $P_{\mathrm{r}}$, resulting from the momentum transfer of escaping water molecules, is defined as the momentum flux normal to the surface and is given by:

$$P_{\mathrm{r}} = J_{\mathrm{vap}} \cdot v_{\mathrm{eff}} \tag{S27}$$

where $v_{\mathrm{eff}}$ is the effective normal velocity of the escaping vapor, expressed as:

$$v_{\mathrm{eff}} = J_{\mathrm{vap}}/\rho_{\mathrm{v}} - J_{\mathrm{vap}}/\rho_{\mathrm{d}} \tag{S28}$$

where the water vapor density $\rho_{\mathrm{v}}$ near the surface (at temperature $T$), estimated using the ideal gas law $\rho_{\mathrm{v}} = M_{\mathrm{w}} P_{\mathrm{v}}/RT$. Given $\rho_{\mathrm{d}} \gg \rho_{\mathrm{v}}$, $P_{\mathrm{r}}$ is derived as:

$$P_{\mathrm{r}} \approx {J_{\mathrm{vap}}}^2/\rho_{\mathrm{v}} = \left(\frac{2\sigma}{2-\sigma}\right)^2 \frac{M_{\mathrm{w}}}{2\pi R}\left(\frac{P_{\mathrm{v}}}{\sqrt{T}} - \frac{P_{\infty}}{\sqrt{T_{\infty}}}\right)^2 \cdot \frac{RT}{M_{\mathrm{w}} P_{\mathrm{v}}} \tag{S29}$$

The vaporization force $F_{\mathrm{vap}}$ driving self-propulsion arises from the $P_{\mathrm{r}}$ difference between the freezing and supercooled regions, acting over the droplet's projected area $\pi {R_{\mathrm{d}}}^2$:

$$F_{\mathrm{vap}} = \begin{cases} \pi {R_{\mathrm{d}}}^2 [P_{\mathrm{r}}(\mathrm{ice}, T_{\mathrm{f}}) - P_{\mathrm{r}}(\text{liquid water}, T_{\mathrm{n}})] & \text{, for pure water} \\ \pi {R_{\mathrm{d}}}^2 \left[P_{\mathrm{r}}\left(\tilde{x}_{\mathrm{g}}, T_{\mathrm{f}}\right) - P_{\mathrm{r}}\left(x_{\mathrm{g}}, T_{\mathrm{n}}\right)\right] & \text{, for glycerol-water} \end{cases} \tag{S30}$$

where the arguments of $P_{\mathrm{r}}$ specify the surface composition and temperature.

$F_{vap}$ is opposed by gravity $F_g = \frac{4}{3}\pi R_d{}^3 \rho_d g$ (with the glycerol-water density $\rho_d$ estimated from its pre-nucleation droplet-average value), and substrate adhesion $F_a$. As nucleation typically initiates at the droplet's free surface (see main text), $F_a$ is attributed primarily to the supercooled region and is estimated using the Young-Dupré equation:

$$F_a = 2\pi R_c \gamma_d (1 + \cos\theta_r) \tag{S31}$$

where $R_c$ is the contact radius (see Supplementary Section 2), $\gamma_d$ is the surface tension of glycerol-water (see equation (S41)), and $\theta_r$ is the receding contact angle (approximated by that of water tested at ambient conditions, see Table S1).

Increasing glycerol concentration has two coupled effects. First, it lowers the equilibrium freezing temperature $T_f$ (Figure S4A). Second, it reduces water activity and hence $P_v$ at a given temperature. We calculated $P_r$ using equation (S29), with $P_v$ for the freezing and supercooled liquid regions evaluated from equation (S33) at their respective states ($(\tilde{x}_g, T_f)$ and $(x_g, T_n)$). These calculations give the glycerol-water results shown by the green lines in Figure S4B. In addition, we evaluated alternative surface composition assumptions (pure ice as magenta lines and pure water as blue lines) to test the sensitivity of $P_r$ to this modeling choice. At a fixed temperature, variations among the composition assumptions change $P_r$ by less than 2 Pa (within either the solid or dashed set of lines). By contrast, the temperature difference between the freezing and supercooled regions produces an order-of-magnitude larger pressure difference (between the corresponding solid and dashed lines). Thus, reduced water activity provides a secondary contribution, while freezing-point depression is the dominant modeled effect over the tested concentration range.

Because $P_r \propto P_v$ (equation (S29)) and $P_v$ increases exponentially with temperature (equation (S33)), $P_r$ is significantly higher in the warmer "freezing" region than in the "supercooled" region (Figure S4B). The temperature difference therefore dominates the $P_r$ difference that determines $F_{vap}$. Consequently, lowering $T_f$ through glycerol addition effectively reduces $F_{vap}$, while the resisting forces $F_g$ and $F_a$ remain relatively constant (Figure 2B), thereby suppressing self-propulsion.

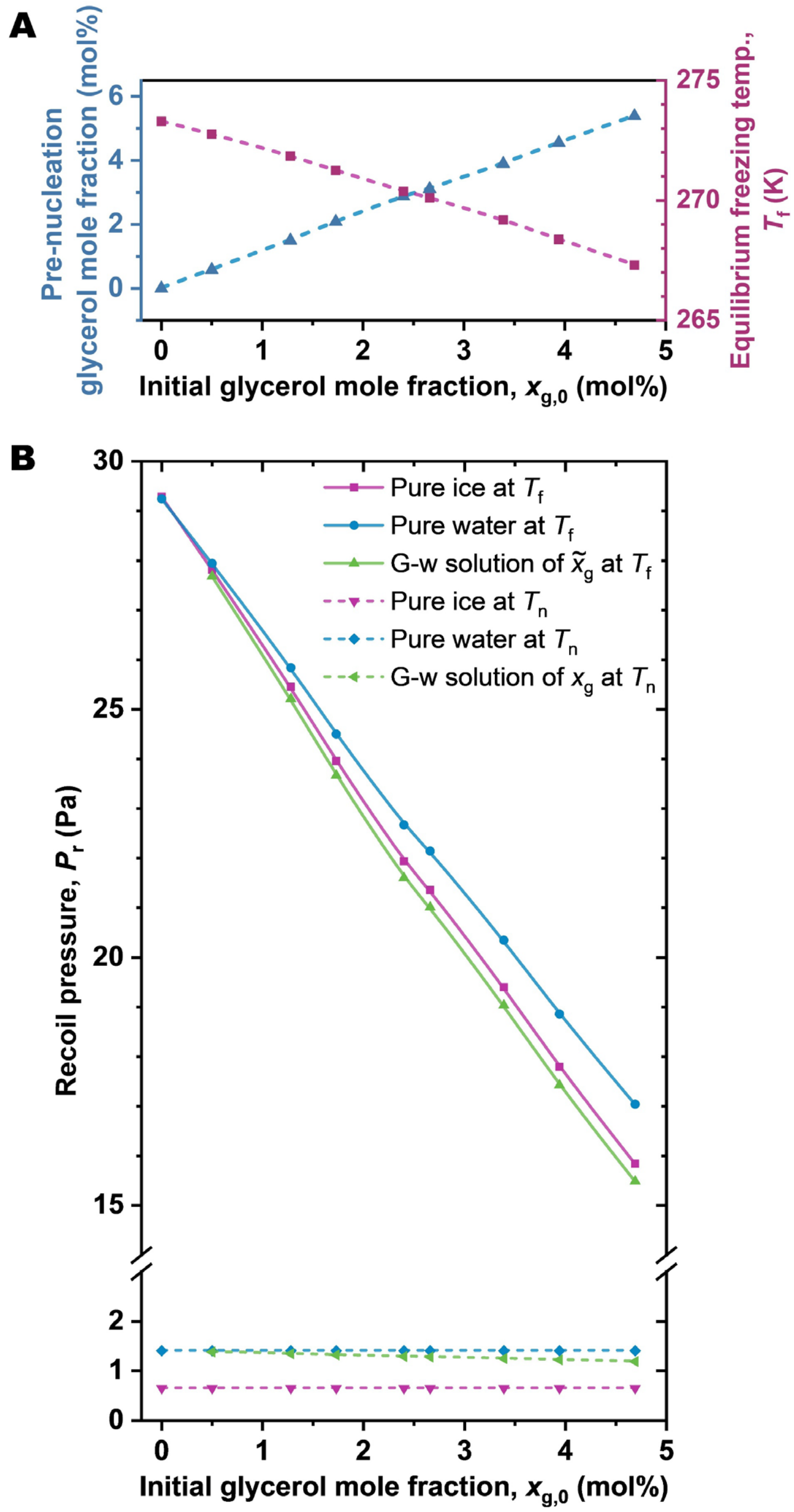


Figure S4. Supplementary analysis of vaporization-driven self-propulsion during recalescence. **(A)** Pre-nucleation droplet-average glycerol mole fraction (blue) and the corresponding equilibrium freezing temperature $T_f$ (magenta), as functions of the initial glycerol mole fraction $x_{g,0}$. The pre-nucleation glycerol mole fraction exhibits a slight increase relative to $x_{g,0}$, whereas $T_f$ decreases monotonically with increasing $x_{g,0}$. **(B)** Recoil pressure $P_r$ as a function of $x_{g,0}$ for different modeled surface states. Green lines use the glycerol-water regional compositions (not applicable at $x_{g,0}$ = 0, which corresponds to pure water), whereas magenta and blue lines use

pure-ice and pure-water assumptions, respectively. Solid lines are evaluated at $T_f$, and dashed lines at $T_n$ using the experimentally measured average nucleation temperature of 250 K. The data in both panels are restricted to $x_{g,0} \leq 4.69$ mol% to exclude non-freezing regime observed experimentally (see Figure 5).

## Supplementary Section 7. Modeling Overpressure Dominated by Free Molecular Flow

The flow regime in the gap beneath the partially frozen droplet is characterized by the Knudsen number $\mathrm{Kn} = \lambda / H_{\mathrm{gap}}$, where $\lambda$ is the molecular mean free path of water vapor and $H_{\mathrm{gap}}$ is the characteristic gap height. The mean free path is defined as $\lambda = k_B T_v / \left(\sqrt{2}\pi d_w{}^2 P_v\right)$, where $k_B$ is the Boltzmann constant, $T_v$ is the vapor temperature, $d_w \approx 0.264$ nm is the effective diameter of a water vapor molecule[49], and $P_v$ is the vapor pressure (see equation (S33)). Because $T_v$ and the local glycerol mole fraction $\tilde{x}_g$ were not measured directly, we bounded them using experimentally and physically motivated ranges. Specifically, $T_v$ was evaluated at post-recalescence bulk droplet temperature range of 247.5 - 265.0 K (Figure S1B), and $\tilde{x}_g$ was varied from 0 - 38 mol%, consistent with the range considered in the main text. These ranges yield $\lambda$ = 35.6 - 252.7 μm. During dwelling, the rigid droplet is supported primarily by the substrate surface structures; therefore, $H_{\mathrm{gap}}$ is approximated by the surface structural height $H_{\mathrm{struc}}$ of the substrate (see Table S1). For the Al-0 substrate, $H_{\mathrm{struc}}$ = 0.973 μm gives Kn = 36.5 - 259.7, supporting treatment of the gap flow as free molecular.

We first evaluated the feasibility of applying the existing overpressure model for slip flow[42], $\Delta P_{\mathrm{op,slip}} \approx 3\mu_v R_c{}^2 J_{\mathrm{vap}} / (H_{\mathrm{gap}}{}^3 F \rho_v)$, with $\rho_v = P_\infty (M_w/R)(1/T)$ and $F = 1 + 12\mathrm{Kn}(2/\Psi - 1)$, to our case. By substituting $\mu_v = 1 \times 10^{-5}$ Pa s, $R_c$ = 0.293 mm, $J_{\mathrm{vap}}$ = 0.156 kg m$^{-2}$ s$^{-1}$, $H_{\mathrm{gap}}$ = 973 nm, Kn = 43.6, $\Psi$ = 0.84, $P_\infty$ = 50 Pa, $M_w = 18.015 \times 10^{-3}$ kg mol$^{-1}$, $R$ = 8.314 J mol$^{-1}$ K$^{-1}$, and $T$ = 265 K, we obtained a significantly overestimated $\Delta P_{\mathrm{op,slip}} \approx 1.47 \times 10^6$ Pa, indicating that this slip flow model is unsuitable for our system.

Our model for the overpressure dominated by free molecular flow is based on the following assumptions:

1. The gap has a thin cylindrical morphology with characteristic radius of contact radius $R_c$ (see Supplementary Section 2), characteristic height of $H_{gap}$, volume of $\pi {R_c}^2 H_{gap}(1 - \varphi_s)$, and lateral surface area of $2\pi R_c H_{gap}(1 - \varphi_s)$, where $\varphi_s$ is the volumetric solid fraction of the substrate surface structures. Notably, both $\varphi_s$ and $H_{gap}$ are eliminated in the final results of this section, see equation (S38).
2. The droplet base is a freeze-concentrated liquid with a local glycerol mole fraction $\tilde{x}_g$.
3. The temperature of substrate surface that is not in direct contact with the droplet is bulk substrate temperature $T_s$ (see also Supplementary Section 8). $T_s$ and the droplet basal temperature $T_{d,base}$ are quasi-steady.
4. Water vapor is the dominant gas in the gap and behaves ideally.
5. Vapor molecules undergo diffuse reflection from the substrate (complete thermal accommodation). This implies that all molecules striking the substrate surface are re-emitted with a Maxwell-Boltzmann velocity distribution corresponding to $T_s$. The vapor temperature in the gap is thus approximated as $T_s$.

The vaporization mass flux from the droplet base $J_{vap,base}$ is given by the Hertz-Knudsen-Schrage equation:

$$J_{\text{vap,base}} = \frac{2\sigma}{2-\sigma}\sqrt{\frac{M_\text{w}}{2\pi R}}\left[\frac{P_\text{v}(\tilde{x}_\text{g}, T_\text{d,base})}{\sqrt{T_\text{d,base}}} - \frac{P_\text{gap}}{\sqrt{T_\text{s}}}\right] \quad \text{(S32)}$$

where the accommodation coefficient $\sigma = 0.457$ is empirically derived from experimental vaporization mass flux (see Supplementary Section 2), $M_w$ is the molar mass of water, $R$ is the universal gas constant, $P_v(\tilde{x}_g, T_{d,base})$ denotes the vapor pressure at the droplet base corresponding to local glycerol mole fraction $\tilde{x}_g$ and droplet basal temperature $T_{d,base}$, and $P_{gap}$ is the gap pressure. Based on the negligible glycerol evaporation due to its low vapor pressure[4], the vapor pressure $P_v$ is defined as:

$$P_\text{v} = \begin{cases} P_\text{w,s} & \text{, for pure ice} \\ P_\text{w,l} & \text{, for pure liquid water} \\ a_\text{w} P_\text{w,l} & \text{, for liquid water in glycerol-water solution} \end{cases} \quad \text{(S33}a\text{)}$$

where the water activity $a_w$ for a glycerol-water solution above its glass transition temperature, $P_{w,s}$, and $P_{w,l}$ are given by[50, 51] (with pressures in Pa and temperature $T$ in K):

$$\ln(a_{\mathrm{w}}) = \ln(1-\varphi_{\mathrm{g}}) + \left(1-\frac{M_{\mathrm{w}}\rho_{\mathrm{g}}}{M_{\mathrm{g}}\rho_{\mathrm{w}}}\right)\varphi_{\mathrm{g}} + \chi{\varphi_{\mathrm{g}}}^2 \tag{S33b}$$

$$\ln(P_{\mathrm{w,s}}) = 9.550426 - \frac{5723.265}{T} + 3.53068\ln(T) - 0.00728332T \tag{S33c}$$

$$\begin{aligned}\ln(P_{\mathrm{w,l}}) &= 54.842763 - \frac{6763.22}{T} - 4.210\ln(T) + 0.000367T \\ &+ \tanh[0.0415(T-218.8)]\cdot\left[53.878 - \frac{1331.22}{T} - 9.44523\ln(T) + 0.014025T\right]\end{aligned} \tag{S33d}$$

where $\varphi_g$ is the glycerol volume fraction, $\chi = 0.29$ is the Flory-Huggins interaction parameter for glycerol-water system. The net rate of water vapor mass change in the gap governs the rate of gap pressure change:

$$\frac{dm_{\mathrm{w,v}}}{dt} = \dot{m}_{\mathrm{in}} - \dot{m}_{\mathrm{out}} \tag{S34a}$$

With $J_{vap,base}$ defined in equation (S32), the mass influx rate into the gap across the contact area $\pi {R_c}^2$ is:

$$\dot{m}_{\mathrm{in}} = \pi {R_{\mathrm{c}}}^2 J_{\mathrm{vap,base}} \tag{S34b}$$

Water vapor escapes from the gap across the lateral surface area at a net mass efflux rate of $\dot{m}_{\mathrm{out}}$. Applying the kinetic theory of gases[52] and approximating the chamber vapor temperature $T_\infty \approx T_s$:

$$\dot{m}_{\mathrm{out}} = 2\pi R_{\mathrm{c}} H_{\mathrm{gap}}(1-\varphi_{\mathrm{s}})\sqrt{\frac{M_{\mathrm{w}}}{2\pi R T_{\mathrm{s}}}}\left(P_{\mathrm{gap}} - P_\infty\right) \tag{S34c}$$

where $P_\infty$ is the chamber pressure. Using the ideal gas law, $P_{\mathrm{gap}}V_{\mathrm{gap}} = (m_{\mathrm{w,v}}/M_{\mathrm{w}})RT_{\mathrm{s}}$, we can relate the mass change to the pressure change:

$$\frac{dP_{\mathrm{gap}}}{dt} = \frac{RT_{\mathrm{s}}}{M_{\mathrm{w}}V_{\mathrm{gap}}}\frac{dm_{\mathrm{w,v}}}{dt} \tag{S35}$$

By combining equation (S32), (S34), and (S35), and substituting gap volume expression $V_{\text{gap}} = \pi R_{\text{c}}^2 H_{\text{gap}}(1-\varphi_{\text{s}})$, we obtain a first-order linear ordinary differential equation for $P_{\text{gap}}$ as a function of $t$:

$$\frac{dP_{\text{gap}}}{dt} = \frac{RT_{\text{s}}}{M_{\text{w}}\pi R_{\text{c}}^2 H_{\text{gap}}(1-\varphi_{\text{s}})}\sqrt{\frac{M_{\text{w}}}{2\pi RT_{\text{s}}}} \cdot \left\{\pi R_{\text{c}}^2 \frac{2\sigma}{2-\sigma}\left[\sqrt{\frac{T_{\text{s}}}{T_{\text{d,base}}}} \cdot P_{\text{v}}\left(\tilde{x}_{\text{g}}, T_{\text{d,base}}\right) - P_{\text{gap}}\right] - 2\pi R_{\text{c}} H_{\text{gap}}(1-\varphi_{\text{s}})\left(P_{\text{gap}} - P_{\infty}\right)\right\} \quad (S36)$$

which solves to:

$$P_{\text{gap}} = C_1 + \left(P_{\text{gap},0} - C_1\right)\exp(-C_2 t) \quad (S37a)$$

$$C_1 = \frac{\frac{2\sigma}{2-\sigma}\sqrt{T_{\text{s}}/T_{\text{d,base}}} \cdot P_{\text{v}}\left(\tilde{x}_{\text{g}}, T_{\text{d,base}}\right) + \frac{2H_{\text{gap}}}{R_{\text{c}}}(1-\varphi_{\text{s}})P_{\infty}}{\frac{2\sigma}{2-\sigma} + \frac{2H_{\text{gap}}}{R_{\text{c}}}(1-\varphi_{\text{s}})} \quad (S37b)$$

$$C_2 = \frac{1}{H_{\text{gap}}(1-\varphi_{\text{s}})}\sqrt{\frac{RT_{\text{s}}}{2\pi M_{\text{w}}}}\left[\frac{2\sigma}{2-\sigma} + \frac{2H_{\text{gap}}}{R_{\text{c}}}(1-\varphi_{\text{s}})\right] \quad (S37c)$$

where $C_1$ and $C_2$ are constants determined by specific experimental conditions, and $P_{\text{gap},0}$ denotes the initial condition $P_{\text{gap},0} \equiv P_{\text{gap}}(t=0)$. For our experimental parameters of micrometer-scale $H_{\text{gap}}$ and millimeter-scale $R_{\text{c}}$, the rate constant $C_2$ is on the order of $10^8$ s$^{-1}$, causing the term $\exp(-C_2 t)$ to decay almost instantaneously. Thus, $P_{\text{gap}}$ rapidly converges to equilibrium gap pressure $P_{\text{gap,equ}} = C_1$. We numerically verified this convergence by solving $P_{\text{gap}}$ in MATLAB. Across initial conditions of $P_{\text{gap},0}$ ranging from 0 to 101 kPa, $P_{\text{gap}}$ converges to $C_1 \pm 0.1\%$ in under 0.1 μs (Figure S5). This timescale is significantly shorter than the observed dwelling times (Figure 1E), justifying the use of an equilibrium model. Since $H_{\text{gap}} \ll R_{\text{c}}$ and $0 < \varphi_{\text{s}} < 1$, the term $\frac{2H_{\text{gap}}}{R_{\text{c}}}(1-\varphi_{\text{s}})$ is much smaller than $\frac{2\sigma}{2-\sigma}$, allowing equation (S37) to be simplified to:

$$P_{\text{gap,equ}} \approx \sqrt{T_{\text{s}}/T_{\text{d,base}}} \cdot P_{\text{v}}\left(\tilde{x}_{\text{g}}, T_{\text{d,base}}\right) \quad (S38)$$

Consequently, the equilibrium overpressure is defined as the pressure difference between $P_{\mathrm{gap,equ}}$ and the chamber pressure $P_\infty$:

$$\Delta P_{\mathrm{op,equ}} = P_{\mathrm{gap,equ}} - P_\infty \approx \sqrt{T_{\mathrm{s}}/T_{\mathrm{d,base}}} \cdot P_{\mathrm{v}}\left(\tilde{x}_{\mathrm{g}}, T_{\mathrm{d,base}}\right) - P_\infty \tag{S39}$$

At the equilibrium gap pressure $P_{\mathrm{gap,equ}}$, vaporization at the droplet base $J_{\mathrm{vap,base}}$ is negligible (see equation (S32) and (S38)). Consequently, there is a net downward vapor recoil pressure $P_{\mathrm{r}}$ acting over the projected contact area $\pi {R_{\mathrm{c}}}^2$ (see equation (S27) – (S29) for $P_{\mathrm{r}}$). Liftoff occurs when the overpressure force $F_{\mathrm{op}} = \pi {R_{\mathrm{c}}}^2 \Delta P_{\mathrm{op,equ}}$ exceeds the combined resisting forces from gravity $F_{\mathrm{g}}$, substrate adhesion $F_{\mathrm{a}}$, and vaporization recoil pressure $\pi {R_{\mathrm{c}}}^2 P_{\mathrm{r}}$. This defines a critical overpressure $\Delta P_{\mathrm{op,crit}}$:

$$\Delta P_{\mathrm{op,crit}} = \left(F_{\mathrm{g}} + F_{\mathrm{a}}\right)/\pi {R_{\mathrm{c}}}^2 + P_{\mathrm{r}} = 4{R_{\mathrm{d}}}^3 \rho_{\mathrm{d}} g / 3{R_{\mathrm{c}}}^2 + 2\gamma_{\mathrm{d}}(1 + \cos\theta_{\mathrm{r}})/R_{\mathrm{c}} + P_{\mathrm{r}} \tag{S40}$$

Here, by neglecting the droplet mass loss after nucleation, gravity is defined as $F_{\mathrm{g}} = \frac{4}{3}\pi {R_{\mathrm{d}}}^3 \rho_{\mathrm{d}} g$, with the equivalent droplet radius $R_{\mathrm{d}}$ estimated by its pre-nucleation value and the glycerol-water density $\rho_{\mathrm{d}}$ estimated by its pre-nucleation droplet-average value (see Supplementary Section 2 for definitions of $R_{\mathrm{d}}$ and $\rho_{\mathrm{d}}$). Considering the droplet's liquid basal layer, the substrate adhesion is estimated using the Young-Dupré equation $F_{\mathrm{a}} = 2\pi R_{\mathrm{c}} \gamma_{\mathrm{d}}(1 + \cos\theta_{\mathrm{r}})$, where $\gamma_{\mathrm{d}}$ is the surface tension for glycerol-water, and $\theta_{\mathrm{r}}$ is the receding contact angle approximated by that of water at ambient conditions (see Table S1 for $\theta_{\mathrm{r}}$). The receding contact angle was not measured in situ during the coupled processes of rapid depressurization, vaporization, freeze-concentration, and partial freezing. These processes may alter the transient contact-line configuration and wetting state on the structured superhydrophobic surface, such that the actual adhesion during liftoff may differ from the present estimate. Consequently, uncertainty in $\theta_{\mathrm{r}}$ propagates into $F_{\mathrm{a}}$ and therefore into $\Delta P_{\mathrm{op,crit}}$. This uncertainty can shift the predicted liftoff threshold quantitatively but does not alter the underlying threshold-crossing criterion: liftoff occurs once the evolving $\Delta P_{\mathrm{op,equ}}$ becomes sufficient to overcome the instantaneous mechanical resistance represented by gravity, adhesion, and vaporization recoil.

The $\gamma_{\mathrm{d}}$ exhibits a nearly linear dependence on glycerol mass fraction $w_{\mathrm{g}}$ between 293 to 353 K[53] and this linear dependence is extended to the experimental temperatures in this study. The

surface tension of pure supercooled water $\gamma_w$ is estimated using an empirical relation[54]. The surface tension of pure glycerol $\gamma_g$ is approximated by a linear extrapolation to lower temperatures based on reported experimental data from 288.15 K to 393.15 K[53, 55-58]. The resulting empirical relations (with surface tension in N m$^{-1}$ and temperature $T$ in K) are:

$$\gamma_{\mathrm{w}} = [75.684 - 0.1376(T - 273.15)] \times 10^{-3} \quad (S41a)$$

$$\gamma_{\mathrm{g}} = (80.556 - 0.06027T) \times 10^{-3} \quad (S41b)$$

$$\gamma_{\mathrm{d}} = w_{\mathrm{g}}\gamma_{\mathrm{g}} + (1 - w_{\mathrm{g}})\gamma_{\mathrm{w}} \quad (S41c)$$

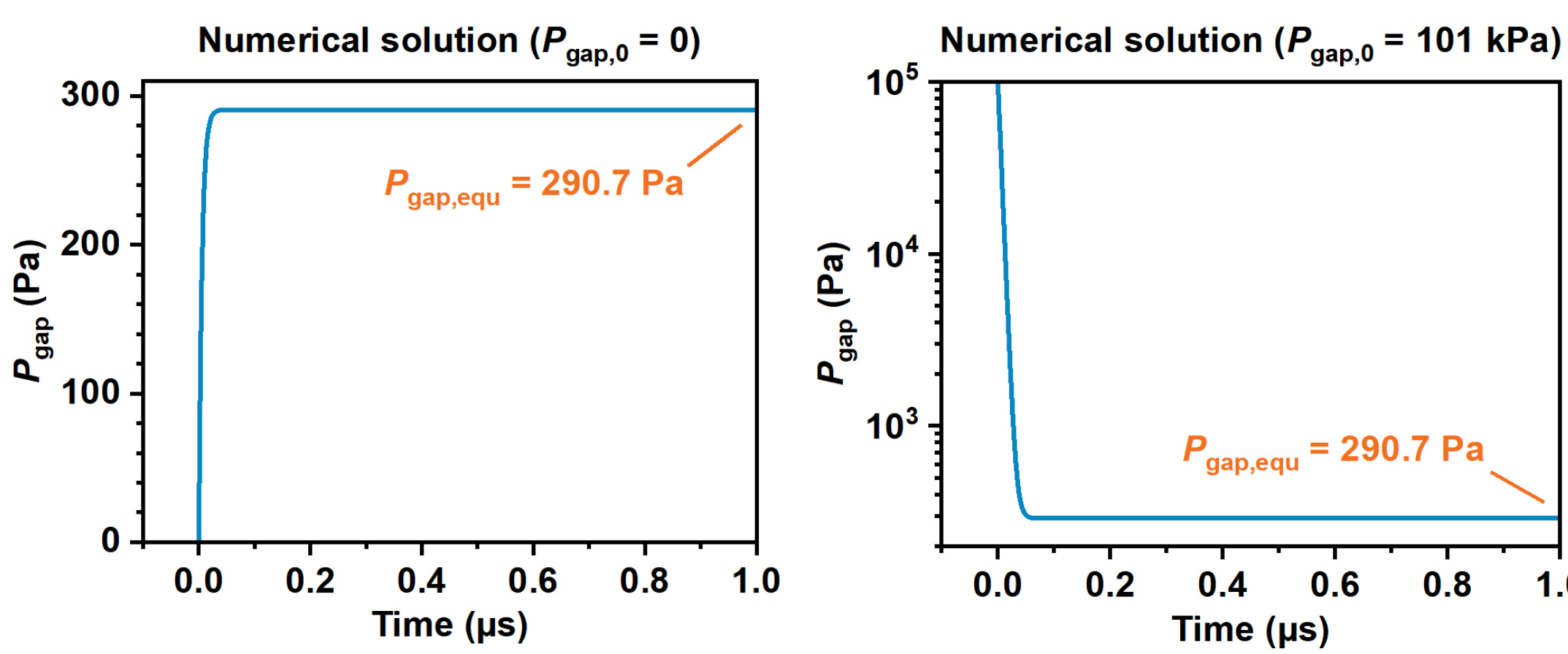


Figure S5. Numerical solutions for the gap pressure $P_{\mathrm{gap}}$ as a function of time. Results are presented for two example initial conditions: $P_{\mathrm{gap},0} = 0$ Pa and $P_{\mathrm{gap},0} = 101$ kPa. Solutions were obtained using the ode15s solver in MATLAB. The solutions demonstrate that $P_{\mathrm{gap}}$ converges rapidly to a steady-state value, $P_{\mathrm{gap,equ}}$, within a timescale significantly shorter than the millisecond-scale dwelling times observed experimentally (Figure 1E). This rapid convergence confirms the validity of using a quasi-static approximation for gap pressure and overpressure. Fixed parameters represent the partially frozen droplet in Figure 1C: $T_{\mathrm{d,base}} = T_{\mathrm{d,bulk}} = 265$ K, $T_{\mathrm{s}} = 292$ K, $H_{\mathrm{gap}} = 973$ nm, $\varphi_{\mathrm{s}} = 0.5$, $R_{\mathrm{c}} = 0.293$ mm, $\tilde{x}_{\mathrm{g}} = 15$ mol%, and $\sigma = 0.457$ (values of $\varphi_{\mathrm{s}}$ and $\tilde{x}_{\mathrm{g}}$ are assumed for qualitative analysis; see other parameter details in Figure S1, Table S1, Figure 1, and Supplementary Section 7).

## Supplementary Section 8. Modeling Heat Transfer at Droplet Base During Dwelling

At the equilibrium gap pressure $P_{\text{gap,equ}}$, vaporization cooling at the droplet base $J_{\text{vap,base}}$ is negligible (see equation (S32) and (S38)). Furthermore, internal convection within the partially frozen droplet is assumed to be suppressed by solidification (see Supplementary Section 3). Therefore, heat influx to the droplet base during the dwelling phase is governed by two parallel mechanisms: solid-liquid heat conduction at discrete contact points and free-molecular heat conduction through the vapor in the confined gap. Notably, in the following derivation, many parameters are functions of the instantaneous state, such as droplet basal temperature $T_{\text{d,base}}(t)$ and local glycerol mole fraction $\tilde{x}_{\text{g}}(t)$. To maintain clarity and avoid overly complex notation, we will not explicitly write these dependencies in every equation, but the functional dependence is implied.

Given that the thermal diffusion length $\sqrt{\alpha t}$ during the 10-millisecond-scale dwelling time is significantly smaller than the characteristic length of both the droplet and substrate, we model both domains as semi-infinite bodies (where $t$ is the time and $\alpha$ is the thermal diffusivity, see Supplementary Section 1 and 2 for $\alpha$).

Solid conduction is driven by the temperature difference between the substrate and the droplet base. Upon contact ($t = 0$), a constant interfacial temperature $T_{\text{int}}$ is established instantaneously, governed by the continuity of heat flux between the two semi-infinite bodies[59]:

$$T_{\text{int}} = T_{\text{s}} - \frac{T_{\text{s}} - T_{\text{d,base,1}}}{1 + e_{\text{s,eff}}/e_{\text{d,base}}} \tag{S42}$$

where $T_{\text{s}}$ is the bulk substrate temperature, $T_{\text{d,base,1}} \equiv T_{\text{d}}(x = 0, t = 0^{-})$ is the pre-dwelling droplet basal temperature ($t = 0^{-}$ indicating before droplet-substrate contact), $e_{\text{s,eff}}$ and $e_{\text{d,base}}$ are the effective thermal effusivity of the substrate and the thermal effusivity of the droplet base, respectively. See Supplementary Section 1 and 2 for $e$. The transient temperature profile $T_{\text{d}}(x, t)$ within the droplet is governed by transient conduction:

$$T_{\text{d}}(x, t) = T_{\text{int}} + \left(T_{\text{d,base,1}} - T_{\text{int}}\right) \cdot \text{erf}\left[\frac{x}{2\sqrt{\alpha_{\text{d,base}} t}}\right] \tag{S43}$$

where $\alpha_{\mathrm{d,base}}$ is the thermal diffusivity of the droplet base evaluated at pre-dwelling droplet basal temperature $T_{\mathrm{d,base,1}}$ and pre-dwelling local glycerol mole fraction $\tilde{x}_{\mathrm{g,1}}$. Differentiating equation (S43) at the interface ($x$ = 0) yields the local heat flux:

$$q_{\mathrm{solid,local}}(t) = -\Lambda_{\mathrm{d,base}}\left(\frac{\partial T_{\mathrm{d}}}{\partial x}\right)\bigg|_{x=0} = \frac{\Lambda_{\mathrm{d,base}}\left(T_{\mathrm{int}} - T_{\mathrm{d,base,1}}\right)}{\sqrt{\pi \alpha_{\mathrm{d,base}} t}} \tag{S44}$$

where $\Lambda_{\mathrm{d,base}}$ is the thermal conductivity of the droplet base (evaluated at pre-dwelling droplet basal temperature $T_{\mathrm{d,base,1}}$ and pre-dwelling local glycerol mole fraction $\tilde{x}_{\mathrm{g,1}}$, see Supplementary Section 2). Averaging this flux over the solid contact fraction $\varphi_{\mathrm{s,contact}}$, we define the effective solid heat transfer coefficient $h_{\mathrm{solid,eff}}$ and the effective solid heat flux $q_{\mathrm{solid,eff}}$:

$$h_{\mathrm{solid,eff}}(t) = \varphi_{\mathrm{s,contact}} \frac{e_{\mathrm{d,base}}}{\sqrt{\pi t}} \tag{S45$a$}$$

$$q_{\mathrm{solid,eff}}(t) = h_{\mathrm{solid,eff}}(t) \cdot \left(T_{\mathrm{int}} - T_{\mathrm{d,base,1}}\right) \tag{S45$b$}$$

In the non-contact regions (fraction 1-$\varphi_{\mathrm{s,contact}}$), heat transfer is governed by free-molecular conduction. The surfaces of the substrate in this region are not in direct contact with the droplet and are not subject to the instantaneous cooling to $T_{\mathrm{int}}$. We therefore approximate the temperature of these surfaces as the bulk substrate temperature $T_{\mathrm{s}}$. The effective heat flux from this mechanism, $q_{\mathrm{gas,eff}}$, is driven by the primary temperature difference $\left(T_{\mathrm{s}} - T_{\mathrm{d,base}}\right)$, where the droplet basal temperature is defined as $T_{\mathrm{d,base}}(t) \equiv T_{\mathrm{d}}(x = 0, t)$ (notably, the dependence of $T_{\mathrm{d,base}}$ on $t$ is not explicitly written in following equations for simplicity):

$$q_{\mathrm{gas,eff}}(t) = h_{\mathrm{gas,eff}}\left(T_{\mathrm{s}} - T_{\mathrm{d,base}}\right) \tag{S46$a$}$$

Assuming complete thermal accommodation and ideal gas behavior (as established in the previous section), the effective gas heat transfer coefficient $h_{\mathrm{gas,eff}}$ is derived from the kinetic theory of gases[52]:

$$h_{\text{gas,eff}} = (1-\varphi_{\text{s,contact}})\frac{\Gamma+1}{\Gamma-1}\sqrt{\frac{R}{8\pi M_{\text{w}}T_{\text{s}}}}P_{\text{gap,equ}}$$
$$= (1-\varphi_{\text{s,contact}})\frac{\Gamma+1}{\Gamma-1}\sqrt{\frac{R}{8\pi M_{\text{w}}T_{\text{d,base}}}}P_{\text{v}}(\tilde{x}_{\text{g}},T_{\text{d,base}}) \quad \text{(S46}b\text{)}$$

where $\Gamma \approx 1.33$ is the specific heat ratio for water vapor, $R$ is the universal gas constant, $M_w$ is the molar mass of water, $P_{gap,equ}$ is the equilibrium gap pressure from equation (S38), and $P_v$ is the vapor pressure (see equation (S33)). Notably, by substituting $P_{\text{gap,equ}}$ from equation (S38), the $\sqrt{T_{\text{s}}}$ term in the denominator of (S46b) is cancelled, resulting in the final dependence on $\sqrt{T_{\text{d,base}}}$ and $P_{\text{v}}(\tilde{x}_{\text{g}},T_{\text{d,base}})$.

The total heat influx to the droplet base, $q_{in}$, is the sum of the solid and gas fluxes:

$$q_{\text{in}}(t) = q_{\text{solid,eff}}(t) + q_{\text{gas,eff}}(t) \quad \text{(S47)}$$

Substituting the expression for $T_{int}$ from (S42) into (S47) yields:

$$q_{\text{in}}(t) = \frac{e_{\text{s,eff}}}{e_{\text{d,base}}+e_{\text{s,eff}}}h_{\text{solid,eff}}(t)\cdot(T_{\text{s}}-T_{\text{d,base,1}}) + h_{\text{gas,eff}}(T_{\text{s}}-T_{\text{d,base}}) \quad \text{(S48)}$$

To construct a unified driving force model, we approximate the constant potential $(T_{\text{s}}-T_{\text{d,base,1}})$ in equation (S48) with the time-dependent potential $(T_{\text{s}}-T_{\text{d,base}})$. Since the droplet heats up over time ($T_{\text{d,base}} \geq T_{\text{d,base,1}}$), this approximation yields a conservative estimate of the heat flux (i.e., underestimating the solid conduction component). This facilitates reformulating the two-driver system as an equivalent single-path model for heat influx $q_{\text{in}}$. By combining equation (S45), (S46), and (S48), we obtain the working expression for $q_{in}$:

$$q_{\text{in}}(t) = h_{\text{in}}(t)\cdot(T_{\text{s}}-T_{\text{d,base}}) \quad \text{(S49}a\text{)}$$

Here, by defining a coefficient $C_{solid}$ for the transient solid conduction term, the effective heat influx coefficient $h_{in}$ is:

$$h_{\text{in}}(t) \approx C_{\text{solid}}t^{-0.5} + h_{\text{gas,eff}} \quad \text{(S49}b\text{)}$$

$$C_{\mathrm{solid}} = \pi^{-0.5}\varphi_{\mathrm{s,contact}}\left(e_{\mathrm{d,base}}{}^{-1} + e_{\mathrm{s,eff}}{}^{-1}\right)^{-1} \tag{S49c}$$

$$h_{\mathrm{gas,eff}}(T_{\mathrm{d,base}}, \tilde{x}_{\mathrm{g}}) = \left(1 - \varphi_{\mathrm{s,contact}}\right)\frac{\Gamma + 1}{\Gamma - 1}\sqrt{\frac{R}{8\pi M_{\mathrm{w}} T_{\mathrm{d,base}}}}P_{\mathrm{v}}\left(\tilde{x}_{\mathrm{g}}, T_{\mathrm{d,base}}\right) \tag{S49d}$$

To elucidate the role of droplet basal heating in generating liftoff overpressure, we analyze the thermal response of the droplet base under two limiting regimes:

**1. Local sensible heating.** In this regime, we assume heat influx does not penetrate to the ice–liquid interface significantly, treating the base as a semi-infinite liquid. The temperature profile $T_{\mathrm{d}}(x,t)$ within the droplet is governed by the 1D transient heat conduction equation:

$$\frac{\partial T_{\mathrm{d}}}{\partial t} = \alpha_{\mathrm{d,base}}\frac{\partial^2 T_{\mathrm{d}}}{\partial x^2} \tag{S50a}$$

subject to the time-dependent Robin boundary condition determined by $q_{\mathrm{in}}$ derived in equation (S49):

$$-\Lambda_{\mathrm{d,base}}\left.\frac{\partial T_{\mathrm{d}}}{\partial x}\right|_{x=0} = q_{\mathrm{in}}(t) = h_{\mathrm{in}}(t)\cdot\left(T_{\mathrm{s}} - T_{\mathrm{d,base}}\right) \tag{S50b}$$

The pre-dwelling droplet is approximated as uniform temperature $T_{\mathrm{d}}(x,0) = T_{\mathrm{d,bulk}}$. We numerically solve this system using the pdepe solver in MATLAB to determine the time $t_{\mathrm{sens}}$ required for $T_{\mathrm{d,base}}$ to rise from its pre-dwelling value $T_{\mathrm{d,base}}(t = 0^-) = T_{\mathrm{d,base,1}}$ to a threshold temperature $T_{\mathrm{d,base}}(t_{\mathrm{sens}}) = T_{\mathrm{d,base,2}}$ corresponding to a target overpressure (e.g., $\Delta P_{\mathrm{op,equ}} = 400$ Pa) for liftoff, assuming the composition remains fixed at the pre-dwelling local glycerol mole fraction $\tilde{x}_{\mathrm{g,1}}$. To accommodate the $t^{-0.5}$ singularity in the $h_{\mathrm{in}}(t)$ term from equation (S49b), the numerical integration is initiated at a computationally small positive time, $t_{\mathrm{ini}} = 10^{-9}$ s, assuming $T_{\mathrm{d,base}}(t_{\mathrm{ini}}) \approx T_{\mathrm{d,base,1}}$.

**2. Local melting.** This regime assumes rapid heat penetration to the ice-liquid interface, maintaining thermodynamic equilibrium, i.e., $T_{\mathrm{d,base}}(t)$ is always equal to the equilibrium freezing temperature $T_{\mathrm{f}}(\tilde{x}_{\mathrm{g}}(t))$, which is a function of the instantaneous mole fraction $\tilde{x}_{\mathrm{g}}(t)$ (notably, the dependence of $\tilde{x}_{\mathrm{g}}$ on $t$ is not explicitly written in following equations for simplicity;

see equation (S24) for $T_f$). As ice melts, the solution dilutes, which in turn raises $T_f(\tilde{x}_g)$. The heat influx $q_{in}$ is balanced by the latent heat of fusion and the heat efflux $q_{out}$ conducting into the ice bulk (with $q_{out}$ derived from a semi-infinite solid approximation):

$$L_{fus}\frac{dm''_w}{dt} = q_{in}(t) - q_{out}(t) \tag{S51a}$$

$$q_{out}(t) = \frac{e_{ice}}{\sqrt{\pi t}} \cdot \left(T_f(\tilde{x}_g) - T_{d,bulk}\right) \tag{S51b}$$

where $L_{fus} \approx 3.34 \times 10^5$ J kg$^{-1}$ is the latent heat of fusion of water, $dm''_w$ is the differential mass of ice melted (per unit area), and $e_{ice} \approx 2.84 \times 10^3$ W s$^{0.5}$ m$^{-2}$ K$^{-1}$ is the thermal effusivity of the ice bulk. The mass melting rate is related to the change in glycerol mole fraction via the conservation of the glycerol mass per unit area $m''_g$:

$$m''_w = m''_g \cdot \frac{M_w}{M_g} \cdot \frac{1 - \tilde{x}_g}{\tilde{x}_g} \tag{S51c}$$

$$m''_g = \tilde{\rho}_d \cdot H_{d,base,1} \cdot \frac{M_g \tilde{x}_{g,1}}{M_g \tilde{x}_{g,1} + M_w(1 - \tilde{x}_{g,1})} \tag{S51d}$$

where $M_w$ and $M_g$ are the molar masses of water and glycerol, respectively, $\tilde{\rho}_d$ is the glycerol-water density based on $\tilde{x}_g$ (see Supplementary Section 2), and $H_{d,base,1}$ is the pre-dwelling thickness of the basal liquid layer. Notably, we tested multiple assumed $H_{d,base,1}$ (0.1, 10, 100 μm) in Figure S6A and C to evaluate the qualitative heat transfer mechanics. Differentiating equation (S51c) with respect to time $t$, combining it with equation (S51a), and substituting equation (S51b) yields the governing ordinary differential equation:

$$\frac{d\tilde{x}_g}{dt} = -\frac{{\tilde{x}_g}^2}{m''_g(M_w/M_g)L_{fus}}\left[h_{in}(t) \cdot \left(T_s - T_f(\tilde{x}_g)\right) - \frac{e_{ice}}{\sqrt{\pi t}}\left(T_f(\tilde{x}_g) - T_{d,bulk}\right)\right] \tag{S52}$$

We numerically integrate equation (S52) using the ode15s solver in MATLAB to determine the time $t_{melt}$ required for the droplet base to dilute from the pre-dwelling state $\tilde{x}_g(t = 0^-) = \tilde{x}_{g,1}$ until a threshold state $\tilde{x}_g(t = t_{melt}) = \tilde{x}_{g,2}$ corresponding to a target overpressure (e.g., $\Delta P_{op,equ}$ = 400 Pa) for liftoff. Similar to the sensible heating model, the solver is initiated at $t_{ini} = 10^{-9}$ s,

assuming $\tilde{x}_{\mathrm{g}}(t_{\mathrm{ini}}) \approx \tilde{x}_{\mathrm{g},1}$, to accommodate the $t^{-0.5}$ singularity in both the $h_{\mathrm{in}}(t)$ and $q_{\mathrm{out}}(t)$ terms.

The local basal temperature $T_{\mathrm{d,base}}$ was not measured directly. We therefore approximate the pre-dwelling value $T_{\mathrm{d,base,1}}$ by the measured bulk temperature $T_{\mathrm{d,bulk}}$, with $\tilde{x}_{\mathrm{g},1}$ for local melting constrained by the equilibrium relation $T_{\mathrm{d,base,1}} = T_{\mathrm{f}}(\tilde{x}_{\mathrm{g},1})$. This approximation is motivated by the heat-flux comparison that is provided later. However, uncertainty in this approximation does not affect the mechanism. For any initial state below the liftoff threshold, basal heat input raises $T_{\mathrm{d,base}}$ and hence equilibrium overpressure $\Delta P_{\mathrm{op,equ}}$ until the critical overpressure $\Delta P_{\mathrm{op,crit}}$ is reached. Changing $T_{\mathrm{d,base,1}}$ changes the predicted heating duration quantitatively but not this modeled pathway. Figure S6A shows an example of the simulated temperature evolution $T_{\mathrm{d,base}}(t)$ during dwelling for both regimes, starting from a reference droplet basal temperature of 260 K. Figure S6B-C varies $T_{\mathrm{d,base,1}}$ to show how its uncertainty affects the calculated sensible-heating time.

As shown in Figure S6B-C, both $t_{\mathrm{sens}}$ and $t_{\mathrm{melt}}$ decrease with increasing $T_{\mathrm{d,base,1}}$, as higher basal temperatures reduce the energy required to reach the target liftoff state. Notably, the result reveals a clear separation in the timescales of the two mechanisms. While both local sensible heating and local melting can theoretically achieve the liftoff state, local melting ($t_{\mathrm{melt}}$) is orders of magnitude slower than local sensible heating ($t_{\mathrm{sens}}$). Importantly, evaluating a range of assumed values for $H_{\mathrm{d,base,1}}$ confirms that the result reveals a robust separation in the timescales of the two mechanisms. In addition, the calculated $t_{\mathrm{sens}}$ aligns quantitatively with the order of magnitude of the experimentally observed dwelling times (ranging from milliseconds to tens of milliseconds, as shown in Figure 1E), suggesting that sensible heating is the dominant mechanism triggering liftoff. The droplet achieves the required overpressure via this rapid heating process well before the slower melting process can contribute substantially.

Furthermore, we assess the magnitude of the vaporization cooling flux $q_{\mathrm{vap}} = J_{\mathrm{vap,base}} L_{\mathrm{vap}}$ during droplet flight by averaging the vaporization cooling fluxes at the pre-dwelling state and the target liftoff state (where $J_{\mathrm{vap,base}}$ is the vaporization mass flux from the droplet base and $L_{\mathrm{vap}}$ is the latent heat of vaporization for supercooled water[60]). During flight, the confined gap is lost and the vapor pressure beneath the droplet is approximated by the chamber pressure, $P_{\mathrm{gap}} \approx P_{\infty}$.

Accordingly, $J_{vap,base}$ during flight is evaluated with equation (S32) using $P_{gap} = P_{\infty}$. By comparing this averaged vaporization cooling flux $\overline{q}_{\mathrm{vap}}$ with the integral-averaged heat influx $\overline{q}_{\mathrm{in}}$ during dwelling, Figure S6D-E confirms that the vaporization cooling flux during flight is at least on the same order of magnitude as the basal heat influx during dwelling ($\overline{q}_{\mathrm{vap}} \gtrsim \overline{q}_{\mathrm{in}}$). This indicates that vaporization cooling is comparably effective in restoring the droplet's basal properties over the 10-millisecond-scale flight time, thereby justifying the use of the bulk droplet temperature to approximate the pre-dwelling state.

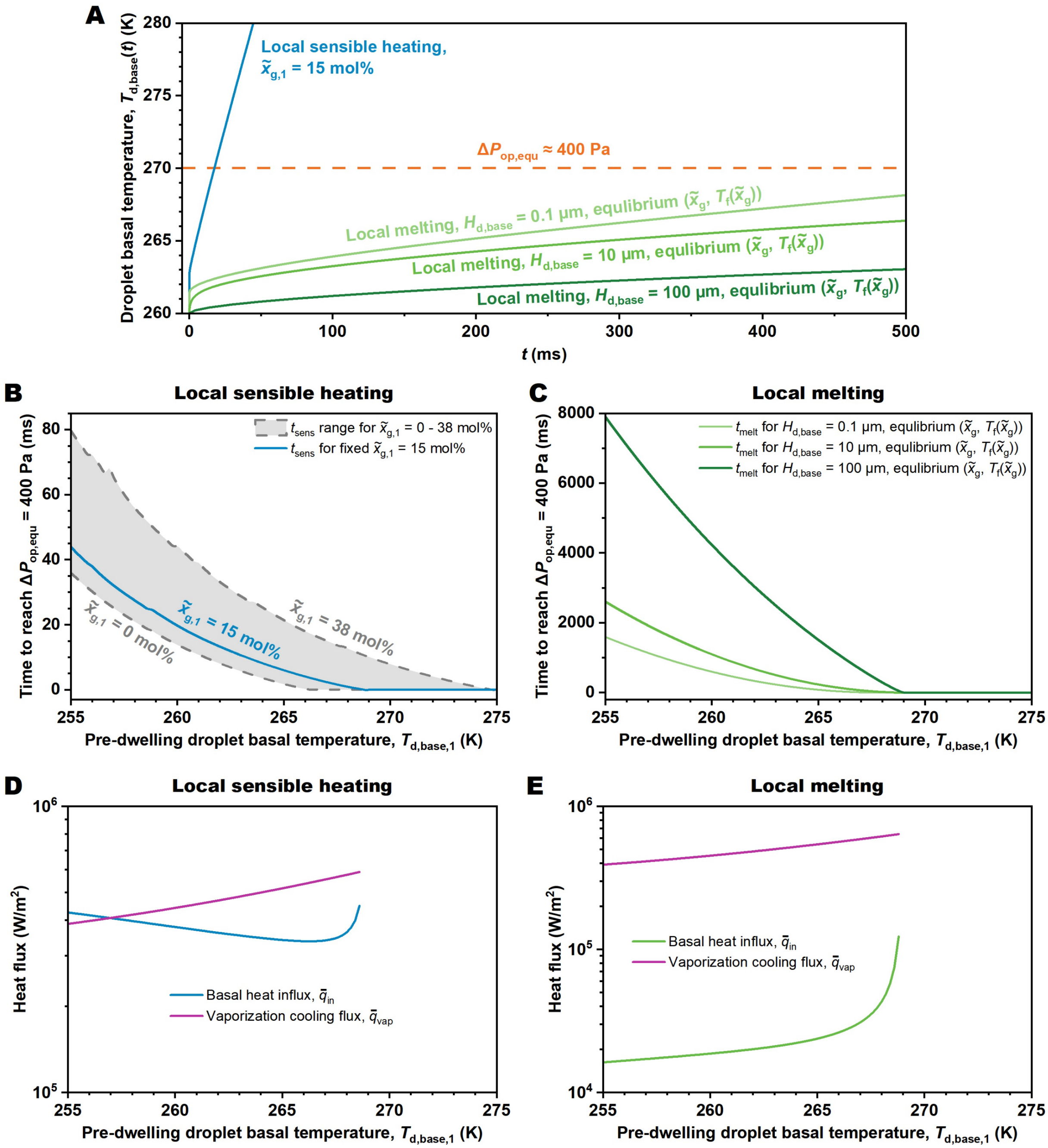


Figure S6. Comparison of local sensible heating and local melting regimes. Fixed parameters for a qualitative evaluation: $T_s$ = 292 K, $P_\infty$ = 50 Pa, $e_{s,eff}$ = 2.40 × $10^4$ W $s^{0.5}$ $m^{-2}$ $K^{-1}$, $\varphi_{s,contact}$ = 0.1. **(A)** Illustrative time-evolution of droplet basal temperature $T_{d,base}(t)$ during dwelling, based on local sensible heating (solid blue line) and local melting (solid green lines), starting from $T_{d,base}$ = 260 K as an example. Results for local sensible heating are shown with pre-dwelling local glycerol mole fraction $\tilde{x}_{g,1}$ = 15 mol%, while results for local melting are shown with initial

basal liquid layer thicknesses $H_{\mathrm{d,base,1}}$ = 0.1, 10, and 100 μm, respectively. The dashed orange line indicates the temperature required to achieve $\Delta P_{\mathrm{op,equ}} \approx 400$ Pa. The two regimes show significant difference in timescales; sensible heating occurs rapidly enough to explain the observed ten-millisecond-scale dwelling, while melting is too slow to contribute significantly. **(B)** Estimated time $t_{\mathrm{sens}}$ required to reach a target $\Delta P_{\mathrm{op,equ}}$ = 400 Pa via sensible heating, as a function of pre-dwelling basal temperature $T_{\mathrm{d,base,1}}$. The gray-shaded region shows the estimated range of $t_{\mathrm{sens}}$, bounded by $\tilde{x}_{\mathrm{g,1}}$ = 0 mol% (pure water) and $\tilde{x}_{\mathrm{g,1}}$ = 38 mol%. The solid blue line shows an example of local sensible heating mechanism for $\tilde{x}_{\mathrm{g,1}} = 15$ mol%. For each $\tilde{x}_{\mathrm{g,1}}$, the corresponding target temperature $T_{\mathrm{d,base,2}}$ for $\Delta P_{\mathrm{op,equ}}$ = 400 Pa is: $T_{\mathrm{d,base,2}}$ = 268.5 K for $\tilde{x}_{\mathrm{g,1}}$ = 0 mol% (pure water); $T_{\mathrm{d,base,2}}$ = 270.9 K for $\tilde{x}_{\mathrm{g,1}}$ = 15 mol%; and $T_{\mathrm{d,base,2}}$ = 276.4 K for $\tilde{x}_{\mathrm{g,1}}$ = 38 mol%. **(C)** Estimated time $t_{\mathrm{melt}}$ required to reach a target $\Delta P_{\mathrm{op,equ}}$ = 400 Pa via local melting, as a function of $T_{\mathrm{d,base,1}} = T_{\mathrm{f}}(\tilde{x}_{\mathrm{g,1}})$. $\Delta P_{\mathrm{op,equ}}$ = 400 Pa corresponds to a single target equilibrium state of $T_{\mathrm{d,base,2}} = T_{\mathrm{f}}(\tilde{x}_{\mathrm{g,2}}) = 269.0$ K (where $\tilde{x}_{\mathrm{g,2}}$ = 4.03 mol%). Results are shown with $H_{\mathrm{d,base,1}}$ = 0.1, 10, and 100 μm, respectively. Each point on the curve represents a state of thermodynamic equilibrium $(\tilde{x}_{\mathrm{g}}, T_{\mathrm{f}}(\tilde{x}_{\mathrm{g}}))$. **(D)** Magnitude comparison of averaged heat influx rate $\overline{q}_{\mathrm{in}}$ during local sensible heating (blue, shown for $\tilde{x}_{\mathrm{g,1}} = 15$ mol%) versus averaged vaporization cooling flux $\overline{q}_{\mathrm{vap}}$ during flight (magenta), as a function of $T_{\mathrm{d,base,1}}$. **(E)** Magnitude comparison of heat influx rate $\overline{q}_{\mathrm{in}}$ during local melting (green, shown for $H_{\mathrm{d,base,1}}$ = 10 μm) versus vaporization cooling flux $\overline{q}_{\mathrm{vap}}$ during flight (magenta), as a function of $T_{\mathrm{d,base,1}}$.

## Supplementary Section 9. Exclusion of Liquid-Liquid or Fragile-Strong Transition

The liquid-liquid transition (LLT) in water, as a first-order-like transition between its low-density liquid (LDL) and high-density liquid (HDL), is considered experimentally inaccessible in bulk pure water. This is attributed to its theoretical occurrence in a “no man’s land” below the homogeneous nucleation temperature of supercooled water and above the crystallization temperature of amorphous ice[61]. However, experimental evidence for water’s LLT has been reported in aqueous solutions where crystallization is suppressed by antifreezes such as

glycerol[62-64]. These findings remain contentious, with some studies suggesting that the observed first-order-like transition may arise from crystallization rather than LLT[41, 64-66].

Because a non-crystallization first-order transition in the glycerol-water system has been (at least theoretically) proposed, here we demonstrate that our experimental conditions, employing positive pressures below 1 atm, are significantly distant from the hypothetical liquid-liquid coexistence line of water, which terminates at the liquid-liquid critical point (LLCP) at ~ 2 kbar[67]. This discrepancy precludes the possibility of LLT as the first-order transition reported in this work.

Furthermore, the possibility of a rapid but continuous fragile-to-strong transition across the Widom line[68-70], which extends from the coexistence line into the single-phase region beyond the LLCP, is also excluded due to significant temperature differences, as illustrated in Figure S7.

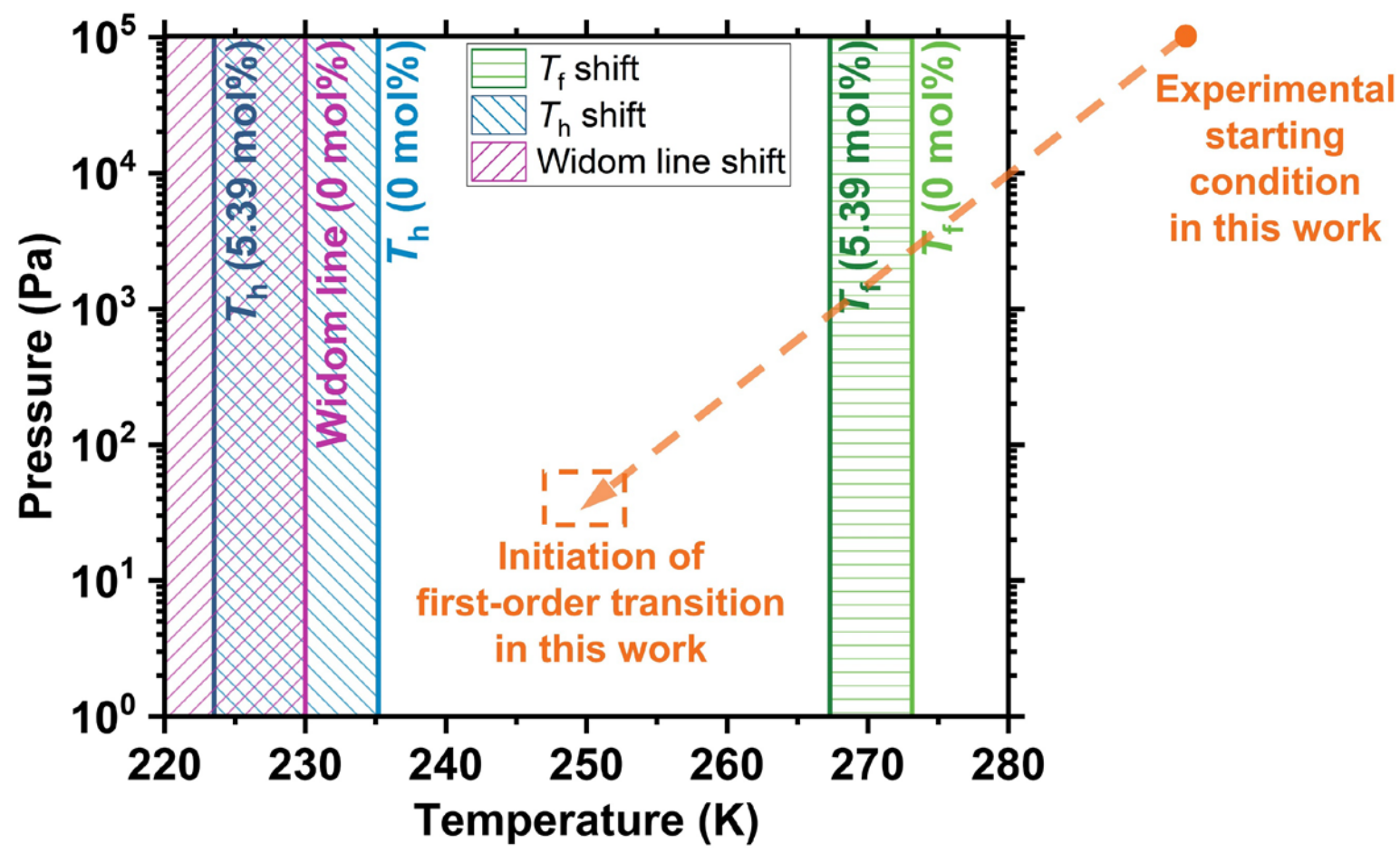


Figure S7. Comparison of the equilibrium freezing temperature $T_f$, homogeneous nucleation temperature $T_h$, and Widom line under experimental conditions, with pressure dependence neglected due to the limited pressure range of 0 to 1 atm. The $T_f$ and $T_h$ are compared between pure water (0 mol%) and glycerol-water solution at the maximum glycerol mole fraction $x_g$ observed to nucleate in our experimental process (a pre-nucleation glycerol mole fraction of 5.39 mol%, which corresponds to a droplet with initial glycerol mole fraction $x_{g,0}$ = 4.69 mol%). The $T_f$ values are detailed in Supplementary Section 6, and $T_h$ values are sourced from Miyata et al. [71]. The Widom line for pure water is approximated by that of the TIP4P/2005 water model[72], while for dilute glycerol-water solutions, only a qualitative temperature-decreasing trend is provided[64]. The color-shaded region illustrates shifts in these parameters due to glycerol addition. The orange dashed arrow with starting point and ending dashed box indicates the starting experimental condition (ambient conditions) and the initiation of first-order transition in this work, respectively, indicating significant temperature differences between the experimental conditions and the estimated Widom line positions. The orange dashed arrow schematically represents our experimental condition, rather than illustrating a literal path of temperature and pressure changes.

## Supplementary Section 10. Nomenclature

Table S4. Nomenclature for symbols.

| Symbol | Definition | Unit |
|---|---|---|
| $A$ | droplet free surface area | $m^2$ |
| Bi | Biot number | dimensionless |
| $C_1$, $C_2$ | constants in the analytical gap pressure solution | Pa; $s^{-1}$ |
| $C_{solid}$ | coefficient of the transient solid conduction term | $W\ s^{0.5}\ m^{-2}\ K^{-1}$ |
| $C^*$ | cubicity of stacking disordered ice | dimensionless |
| $c_p$ | isobaric specific heat capacity | $J\ kg^{-1}\ K^{-1}$ |
| $c_w$ | water mass concentration | $kg\ m^{-3}$ |
| $D_{mass}$ | glycerol-water mutual mass diffusion coefficient | $m^2\ s^{-1}$ |
| $d_w$ | effective molecular diameter of water vapor | m |
| $e$ | thermal effusivity; in the MD force field description, elementary charge | $W\ s^{0.5}\ m^{-2}\ K^{-1}$; C |
| $E$ | molecular potential energy contribution | $J\ mol^{-1}$ |
| $F$ | rarefaction correction factor in the slip-flow overpressure model | dimensionless |
| $F_a$, $F_g$, $F_{op}$, $F_{vap}$ | substrate adhesion, gravitational, basal-overpressure, and net vaporization forces | N |
| $g$ | gravitational acceleration | $m\ s^{-2}$ |
| $H_{gap}$ | characteristic basal gap height | m |
| $H_{struc}$ | substrate structural height | m |
| $H_{d,base,1}$ | initial basal liquid layer thickness | m |
| $h$ | visible vapor cushion thickness | m |
| $h_{gas,eff}$, $h_{solid,eff}$, $h_{in}$ | effective gas conduction, solid conduction, and inward heat transfer coefficients | $W\ m^{-2}\ K^{-1}$ |
| $J_{dif}$ | diffusive water mass flux | $kg\ m^{-2}\ s^{-1}$ |
| $J_{vap}$, $J_{vap,base}$ | surface and basal vaporization mass fluxes | $kg\ m^{-2}\ s^{-1}$ |
| $k_B$ | Boltzmann constant | $J\ K^{-1}$ |
| $K_b$, $K_\alpha$, $K_\Phi$ | bond, angle, and torsional force constants | see Table S3 |
| Kn | Knudsen number | dimensionless |
| $L_{fus}$, $L_{solid}$, $L_{vap}$ | latent heats of fusion, solidification, and vaporization | $J\ kg^{-1}$ |
| $m$ | mass | kg |
| $\dot{m}_{in}$, $\dot{m}_{out}$ | vapor mass inflow and outflow rates | $kg\ s^{-1}$ |
| $\dot{m}_{vap}$ | vaporization mass rate | $kg\ s^{-1}$ |
| $m''$ | mass per unit area | $kg\ m^{-2}$ |
| $M_g$, $M_w$ | molar masses of glycerol and water | $kg\ mol^{-1}$ |
| $n$ | number of independent experiments; molecular count, molar quantity, or torsional multiplicity, as specified | dimensionless |
| $P_\infty$ | chamber pressure | Pa |
| $P_{gap}$, $P_{gap,0}$, $P_{gap,equ}$ | instantaneous gap pressure, initial gap pressure, and equilibrium gap pressure | Pa |
| $P_r$ | vaporization recoil pressure | Pa |
| $P_v$ | vapor pressure | Pa |
| $P_{w,l}$, $P_{w,s}$ | vapor pressures of liquid water and ice | Pa |
| $p$ | hydrodynamic pressure in the liquid momentum equation | Pa |

| | | |
|---|---|---|
| $\Delta P_{op,equ}$, $\Delta P_{op,crit}$, $\Delta P_{op,slip}$ | equilibrium overpressure, critical overpressure for liftoff, and slip-model overpressure | Pa |
| $Pe_{mass}$, $Pe_{thermal}$ | mass and thermal Péclet numbers | dimensionless |
| $q_{gas,eff}$, $q_{solid,eff}$, $q_{solid,local}$ | effective gas conduction, effective solid conduction, and local solid conduction heat fluxes | W m$^{-2}$ |
| $q_{in}$, $q_{out}$, $q_{vap}$ | heat influx, heat efflux, and vaporization cooling flux | W m$^{-2}$ |
| $q_i$, $q_j$ | partial atomic charges in the force field | elementary-charge units |
| $R$ | universal gas constant | J mol$^{-1}$ K$^{-1}$ |
| $R_c$, $R_d$ | droplet-substrate contact radius and equivalent droplet radius | m |
| Re | Reynolds number | dimensionless |
| $r$, $r_0$, $r_{ij}$ | bond length, equilibrium bond length, and interatomic distance | m |
| $T$ | temperature | K |
| $T_{d,base}$, $T_{d,bulk}$ | basal and bulk droplet temperatures | K |
| $T_f$, $T_{gt}$, $T_h$, $T_{int}$, $T_n$ | equilibrium freezing, glass transition, homogeneous nucleation, interfacial, and experimental nucleation temperatures | K |
| $T_s$, $T_v$, $T_\infty$ | bulk substrate, vapor, and chamber vapor temperatures | K |
| $t$, $t_{ini}$, $t_{melt}$, $t_{sens}$, $t_n$ | time, numerical start time, melting time, sensible heating time, and nucleation time | s |
| $u$ | characteristic liquid velocity | m s$^{-1}$ |
| $v_{eff}$ | effective normal velocity of vapor leaving the interface | m s$^{-1}$ |
| $V_d$, $V_{gap}$ | droplet and basal gap volumes | m$^3$ |
| $w_g$, $w_w$, $\tilde{w}_i$ | glycerol, liquid water, and local ice mass fractions | dimensionless |
| $x$ | coordinate normal to the droplet base | m |
| $x_g$, $x_{g,0}$, $\tilde{x}_g$ | droplet-average, initial, and local glycerol mole fractions | dimensionless |
| $x_{i,total}$ | system-average ice mole fraction | dimensionless |
| $z$ | droplet center height measured from the contact line | m |
| $a_w$ | water activity | dimensionless |
| $\alpha$, $\alpha_0$ | thermal diffusivity; in the MD force field, actual and equilibrium bond angles | m$^2$ s$^{-1}$; rad |
| $\alpha_{expan}$ | volumetric thermal expansion coefficient | K$^{-1}$ |
| $\beta$ | viscosity correlation exponent | dimensionless |
| $\gamma_d$, $\gamma_g$, $\gamma_w$ | surface tensions of glycerol-water, glycerol, and water | N m$^{-1}$ |
| $\Gamma$ | heat capacity ratio of water vapor | dimensionless |
| $\delta_{dif}$ | mass diffusion boundary layer thickness | m |
| $\varepsilon_{ij}$, $\sigma_{ij}$ | Lennard-Jones energy and length parameters | J mol$^{-1}$; m |
| $\theta_r$ | receding contact angle | degree |
| $\Lambda$ | thermal conductivity | W m$^{-1}$ K$^{-1}$ |
| $\lambda$ | molecular mean free path of water vapor | m |
| $\mu_d$, $\mu_v$ | droplet-average liquid viscosity, vapor viscosity | Pa s |
| $\rho_d$, $\rho_g$, $\rho_i$, $\rho_v$, $\rho_w$ | droplet-average, glycerol, ice, vapor, and liquid-water densities | kg m$^{-3}$ |
| $\sigma$ | accommodation coefficient in the Hertz-Knudsen-Schrage equation | dimensionless |
| $\tau_{dif}$, $\tau_{mass}$, $\tau_{thermal}$ | diffusion exposure, mass diffusion, and thermal diffusion times | s |
| $\varphi_g$, $\varphi_s$, $\varphi_{s,contact}$ | glycerol volume fraction, substrate solid fraction, and contact area fraction | dimensionless |
| $\chi$ | Flory-Huggins interaction parameter | dimensionless |
| $\Psi$ | accommodation coefficient in the slip-flow overpressure model | dimensionless |
| $\Phi$ | torsional angle | rad |

| | | |
|---|---|---|
| $\Delta c_w$, $\Delta \rho_d$, $\Delta w_w$ | water concentration, density, and water mass fraction differences between bulk and surface | kg $m^{-3}$; kg $m^{-3}$; dimensionless |

| Subscript | Meaning | Subscript | Meaning |
|---|---|---|---|
| 0 | initial state | 1, 2 | initial and target local states |
| base | droplet base | bulk | bulk droplet |
| c | contact | d | droplet or glycerol-water mixture average |
| eff | effective | equ | equilibrium |
| g | glycerol | gap | basal vapor gap |
| i | ice | in, out | influx and outflow |
| int | interface | l, s | liquid and solid water, when attached to $P_w$ |
| n | nucleation | op | overpressure |
| r | recoil or receding, as specified | s | substrate, unless attached to $P_w$ |
| v | vapor | w | water |
| ∞ | chamber environment | | |

## SUPPLEMENTARY REFERENCES


(1) Speight, J. *Lange's Handbook of Chemistry, Seventeenth Edition*; McGraw Hill LLC, 2016.

(2) Mark, J. E. *Polymer Data Handbook, Second Edition*; Oxford University Press, 2009.

(3) Andersson, S.; Dzhavadov, L. Thermal conductivity and heat capacity of amorphous SiO2: pressure and volume dependence. *Journal of Physics: Condensed Matter* **1992**, *4* (29), 6209. DOI: 10.1088/0953-8984/4/29/005.

(4) Verevkin, S. P.; Zaitsau, D. H.; Emel'yanenko, V. N.; Zhabina, A. A. Thermodynamic properties of glycerol: Experimental and theoretical study. *Fluid Phase Equilibria* **2015**, *397*, 87-94. DOI: 10.1016/j.fluid.2015.03.038.

(5) Blazhnov, I. V.; Malomuzh, N. P.; Lishchuk, S. V. Temperature dependence of density, thermal expansion coefficient and shear viscosity of supercooled glycerol as a reflection of its structure. *The Journal of Chemical Physics* **2004**, *121* (13), 6435-6441. DOI: 10.1063/1.1789474.

(6) Hare, D. E.; Sorensen, C. M. The density of supercooled water. II. Bulk samples cooled to the homogeneous nucleation limit. *The Journal of Chemical Physics* **1987**, *87* (8), 4840-4845. DOI: 10.1063/1.453710.

(7) Volk, A.; Kähler, C. J. Density model for aqueous glycerol solutions. *Experiments in Fluids* **2018**, *59* (5), 75. DOI: 10.1007/s00348-018-2527-y.

(8) Bioucas, F. E. B.; Koller, T. M.; Fröba, A. P. Thermal Conductivity of Glycerol at Atmospheric Pressure Between 268 K and 363 K by Using a Steady-State Parallel-Plate Instrument. *International Journal of Thermophysics* **2024**, *45* (4), 52. DOI: 10.1007/s10765-024-03347-x.

(9) Ramires, M. L. V.; Nieto de Castro, C. A.; Nagasaka, Y.; Nagashima, A.; Assael, M. J.; Wakeham, W. A. Standard Reference Data for the Thermal Conductivity of Water. *Journal of Physical and Chemical Reference Data* **1995**, *24* (3), 1377-1381. DOI: 10.1063/1.555963.

(10) Bastos, M.; Nilsson, S.-O.; Ribeiro da Silva, M. D. M. C.; Ribeiro da Silva, M. A. V.; Wadsö, I. Thermodynamic properties of glycerol enthalpies of combustion and vaporization and the heat capacity at 298.15 K. Enthalpies of solution in water at 288.15, 298.15, and 308.15 K. *The Journal of Chemical Thermodynamics* **1988**, *20* (11), 1353-1359. DOI: 10.1016/0021-9614(88)90173-5.

(11) Holten, V.; Bertrand, C. E.; Anisimov, M. A.; Sengers, J. V. Thermodynamics of supercooled water. *The Journal of Chemical Physics* **2012**, *136* (9). DOI: 10.1063/1.3690497.

(12) Thielicke, W.; Sonntag, R. Particle Image Velocimetry for MATLAB: Accuracy and enhanced algorithms in PIVlab. *Journal of Open Research Software* **2021**. DOI: 10.5334/jors.334.

(13) Dash, S.; Chandramohan, A.; Weibel, J. A.; Garimella, S. V. Buoyancy-induced on-the-spot mixing in droplets evaporating on nonwetting surfaces. *Physical Review E* **2014**, *90* (6), 062407. DOI: 10.1103/PhysRevE.90.062407.

(14) Yim, E.; Bouillant, A.; Gallaire, F. Buoyancy-driven convection of droplets on hot nonwetting surfaces. *Physical Review E* **2021**, *103* (5), 053105. DOI: 10.1103/PhysRevE.103.053105.

(15) Yu, J.; Pan, Z.; Weibel, J. A. Transition of buoyancy-driven flow from an axisymmetric to non-axisymmetric vortex inside an evaporating sessile droplet. *Journal of Fluid Mechanics* **2024**, *997*, A47. DOI: 10.1017/jfm.2024.628.
(16) Yan, X.; Au, S. C. Y.; Chan, S. C.; Chan, Y. L.; Leung, N. C.; Wu, W. Y.; Sin, D. T.; Zhao, G.; Chung, C. H. Y.; Mei, M.; et al. Unraveling the role of vaporization momentum in self-jumping dynamics of freezing supercooled droplets at reduced pressures. *Nature Communications* **2024**, *15* (1), 1567. DOI: 10.1038/s41467-024-45928-2.
(17) Lambert, J. H. *Photometria sive de mensura et gradibus luminis, colorum et umbrae*; sumptibus vidvae E. Klett, typis CP Detleffsen, 1760.
(18) Trejo González, J. A.; Longinotti, M. P.; Corti, H. R. The Viscosity of Glycerol−Water Mixtures Including the Supercooled Region. *Journal of Chemical & Engineering Data* **2011**, *56* (4), 1397-1406. DOI: 10.1021/je101164q.
(19) D'Errico, G.; Ortona, O.; Capuano, F.; Vitagliano, V. Diffusion Coefficients for the Binary System Glycerol + Water at 25 °C. A Velocity Correlation Study. *Journal of Chemical & Engineering Data* **2004**, *49* (6), 1665-1670. DOI: 10.1021/je049917u.
(20) Cristancho, D. M.; Delgado, D. R.; Martínez, F.; Abolghassemi Fakhree, M. A.; Jouyban, A. Volumetric properties of glycerol+ water mixtures at several temperatures and correlation with the Jouyban-Acree model. *Revista colombiana de ciencias quimico-farmaceuticas* **2011**, *40* (1), 92-115.
(21) Higbie, R. The rate of absorption of pure gas into a still liquid during short periods of exposure. *Trans. Am. Inst. Chem. Engrs.* **1935**, *31*, 365-389.
(22) Thompson, A. P.; Aktulga, H. M.; Berger, R.; Bolintineanu, D. S.; Brown, W. M.; Crozier, P. S.; in 't Veld, P. J.; Kohlmeyer, A.; Moore, S. G.; Nguyen, T. D.; et al. LAMMPS - a flexible simulation tool for particle-based materials modeling at the atomic, meso, and continuum scales. *Computer Physics Communications* **2022**, *271*, 108171. DOI: 10.1016/j.cpc.2021.108171.
(23) Abascal, J. L. F.; Sanz, E.; García Fernández, R.; Vega, C. A potential model for the study of ices and amorphous water: TIP4P/Ice. *The Journal of Chemical Physics* **2005**, *122* (23). DOI: 10.1063/1.1931662.
(24) Molinero, V.; Moore, E. B. Water Modeled As an Intermediate Element between Carbon and Silicon. *The Journal of Physical Chemistry B* **2009**, *113* (13), 4008-4016. DOI: 10.1021/jp805227c.
(25) Bussi, G.; Donadio, D.; Parrinello, M. Canonical sampling through velocity rescaling. *The Journal of Chemical Physics* **2007**, *126* (1). DOI: 10.1063/1.2408420.
(26) Parrinello, M.; Rahman, A. Polymorphic transitions in single crystals: A new molecular dynamics method. *Journal of Applied Physics* **1981**, *52* (12), 7182-7190. DOI: 10.1063/1.328693.
(27) Egorov, A. V.; Lyubartsev, A. P.; Laaksonen, A. Molecular Dynamics Simulation Study of Glycerol–Water Liquid Mixtures. *The Journal of Physical Chemistry B* **2011**, *115* (49), 14572-14581. DOI: 10.1021/jp208758r.
(28) Chelli, R.; Procacci, P.; Cardini, G.; G. Della Valle, R.; Califano, S. Glycerol condensed phases Part I. A molecular dynamics study. *Physical Chemistry Chemical Physics* **1999**, *1* (5), 871-877, 10.1039/A808958B. DOI: 10.1039/A808958B.

(29) Blieck, J.; Affouard, F.; Bordat, P.; Lerbret, A.; Descamps, M. Molecular dynamics simulations of glycerol glass-forming liquid. *Chemical Physics* **2005**, *317* (2), 253-257. DOI: 10.1016/j.chemphys.2005.05.045.
(30) Reiling, S.; Schlenkrich, M.; Brickmann, J. Force field parameters for carbohydrates. *Journal of Computational Chemistry* **1996**, *17* (4), 450-468. DOI: 10.1002/(SICI)1096-987X(199603)17:4<450::AID-JCC6>3.0.CO;2-T.
(31) Bernal, J. D.; Fowler, R. H. A Theory of Water and Ionic Solution, with Particular Reference to Hydrogen and Hydroxyl Ions. *The Journal of Chemical Physics* **1933**, *1* (8), 515-548. DOI: 10.1063/1.1749327.
(32) Nguyen, A. H.; Molinero, V. Identification of Clathrate Hydrates, Hexagonal Ice, Cubic Ice, and Liquid Water in Simulations: the CHILL+ Algorithm. *The Journal of Physical Chemistry B* **2015**, *119* (29), 9369-9376. DOI: 10.1021/jp510289t.
(33) Murray, B. J.; Knopf, D. A.; Bertram, A. K. The formation of cubic ice under conditions relevant to Earth's atmosphere. *Nature* **2005**, *434* (7030), 202-205. DOI: 10.1038/nature03403.
(34) Hansen, T. C.; Koza, M. M.; Kuhs, W. F. Formation and annealing of cubic ice: I. Modelling of stacking faults. *Journal of Physics: Condensed Matter* **2008**, *20* (28), 285104. DOI: 10.1088/0953-8984/20/28/285104.
(35) Moore, E. B.; Molinero, V. Is it cubic? Ice crystallization from deeply supercooled water. *Physical Chemistry Chemical Physics* **2011**, *13* (44), 20008-20016. DOI: 10.1039/C1CP22022E.
(36) Malkin, T. L.; Murray, B. J.; Brukhno, A. V.; Anwar, J.; Salzmann, C. G. Structure of ice crystallized from supercooled water. *Proceedings of the National Academy of Sciences* **2012**, *109* (4), 1041-1045. DOI: 10.1073/pnas.1113059109.
(37) Johnston, J. C.; Molinero, V. Crystallization, Melting, and Structure of Water Nanoparticles at Atmospherically Relevant Temperatures. *Journal of the American Chemical Society* **2012**, *134* (15), 6650-6659. DOI: 10.1021/ja210878c.
(38) Thürmer, K.; Nie, S. Formation of hexagonal and cubic ice during low-temperature growth. *Proceedings of the National Academy of Sciences* **2013**, *110* (29), 11757-11762. DOI: 10.1073/pnas.1303001110.
(39) Amaya, A. J.; Pathak, H.; Modak, V. P.; Laksmono, H.; Loh, N. D.; Sellberg, J. A.; Sierra, R. G.; McQueen, T. A.; Hayes, M. J.; Williams, G. J.; et al. How Cubic Can Ice Be? *The Journal of Physical Chemistry Letters* **2017**, *8* (14), 3216-3222. DOI: 10.1021/acs.jpclett.7b01142.
(40) Li, M.; Zhang, J.; Niu, H.; Lei, Y.-K.; Han, X.; Yang, L.; Ye, Z.; Yang, Y. I.; Gao, Y. Q. Phase Transition between Crystalline Variants of Ordinary Ice. *The Journal of Physical Chemistry Letters* **2022**, *13* (36), 8601-8606. DOI: 10.1021/acs.jpclett.2c02176.
(41) Alba-Simionesco, C.; Judeinstein, P.; Longeville, S.; Osta, O.; Porcher, F.; Caupin, F.; Tarjus, G. Interplay of vitrification and ice formation in a cryoprotectant aqueous solution at low temperature. *Proceedings of the National Academy of Sciences* **2022**, *119* (12), e2112248119. DOI: 10.1073/pnas.2112248119.
(42) Schutzius, T. M.; Jung, S.; Maitra, T.; Graeber, G.; Köhme, M.; Poulikakos, D. Spontaneous droplet trampolining on rigid superhydrophobic surfaces. *Nature* **2015**, *527* (7576), 82-85. DOI: 10.1038/nature15738.

(43) Lambley, H.; Graeber, G.; Vogt, R.; Gaugler, L. C.; Baumann, E.; Schutzius, T. M.; Poulikakos, D. Freezing-induced wetting transitions on superhydrophobic surfaces. *Nature Physics* **2023**, *19* (5), 649-655. DOI: 10.1038/s41567-023-01946-3.
(44) Stan, C. A.; Kalita, A.; Marte, S.; Kaldawi, T. F.; Willmott, P. R.; Boutet, S. Rocket drops: The self-propulsion of supercooled freezing drops. *Physical Review Fluids* **2023**, *8* (2), L021601. DOI: 10.1103/PhysRevFluids.8.L021601.
(45) Boreyko, J. B. Freeze in or breeze out. *Nature Physics* **2023**, *19* (5), 609-610. DOI: 10.1038/s41567-023-01964-1.
(46) Meng, Z.; Zhang, P. Freezing dynamics of supercooled micro-sized water droplets. *International Journal of Heat and Mass Transfer* **2022**, *193*, 122955. DOI: 10.1016/j.ijheatmasstransfer.2022.122955.
(47) Chaudhary, G.; Li, R. Freezing of water droplets on solid surfaces: An experimental and numerical study. *Experimental Thermal and Fluid Science* **2014**, *57*, 86-93. DOI: 10.1016/j.expthermflusci.2014.04.007.
(48) Lane, L. B. Freezing Points of Glycerol and Its Aqueous Solutions. *Industrial & Engineering Chemistry* **1925**, *17* (9), 924-924.
(49) Poling, B. E.; Prausnitz, J. M.; O'connell, J. P. *The properties of gases and liquids*; Mcgraw-hill New York, 2001.
(50) He, X.; Fowler, A.; Toner, M. Water activity and mobility in solutions of glycerol and small molecular weight sugars: Implication for cryo- and lyopreservation. *Journal of Applied Physics* **2006**, *100* (7). DOI: 10.1063/1.2336304.
(51) Murphy, D. M.; Koop, T. Review of the vapour pressures of ice and supercooled water for atmospheric applications. *Quarterly Journal of the Royal Meteorological Society* **2005**, *131* (608), 1539-1565. DOI: 10.1256/qj.04.94.
(52) C, S. Kinetic Theory of Gases. *Nature* **1938**, *142* (3594), 494-495. DOI: 10.1038/142494a0.
(53) Takamura, K.; Fischer, H.; Morrow, N. R. Physical properties of aqueous glycerol solutions. *Journal of Petroleum Science and Engineering* **2012**, *98-99*, 50-60. DOI: 10.1016/j.petrol.2012.09.003.
(54) Kalová, J.; Mareš, R. Surface Tension in the Supercooled Water Region. *International Journal of Thermophysics* **2021**, *42* (9), 131. DOI: 10.1007/s10765-021-02884-z.
(55) Mori, Y. H.; Tsui, N.; Kiyomiya, M. Surface and interfacial tensions and their combined properties in seven binary, immiscible liquid-liquid-vapor systems. *Journal of Chemical & Engineering Data* **1984**, *29* (4), 407-412. DOI: 10.1021/je00038a012.
(56) Iqbal, M. J.; Rauf, M. A.; Ijaz, N. Surface tension measurements of glycerol with organic cosolvents. *Journal of Chemical & Engineering Data* **1992**, *37* (1), 45-47. DOI: 10.1021/je00005a015.
(57) Erfani, A.; Khosharay, S.; Aichele, C. P. Surface tension and interfacial compositions of binary glycerol/alcohol mixtures. *The Journal of Chemical Thermodynamics* **2019**, *135*, 241-251. DOI: 10.1016/j.jct.2019.03.014.
(58) Liu, L.; Shuo, G.; Chen, J.; Wang, Z.; Du, W.; Li, B. Density and surface tension of binary mixtures of 1, 2-propylene glycol and glycerol under atmospheric pressure from 303.15 K to 393.15 K and correlating with the Jouyban–Acree model. *Physics and Chemistry of Liquids* **2022**, *60* (5), 682-695. DOI: 10.1080/00319104.2022.2032053.

(59) Carslaw, H.; Jaeger, J. *Conduction of heat in solids, Clarendon*; Oxford, 1959.

(60) Henderson-Sellers, B. A new formula for latent heat of vaporization of water as a function of temperature. *Quarterly Journal of the Royal Meteorological Society* **1984**, *110* (466), 1186-1190. DOI: 10.1002/qj.49711046626.

(61) Mishima, O.; Stanley, H. E. The relationship between liquid, supercooled and glassy water. *Nature* **1998**, *396* (6709), 329-335. DOI: 10.1038/24540.

(62) Murata, K.-i.; Tanaka, H. Liquid–liquid transition without macroscopic phase separation in a water–glycerol mixture. *Nature Materials* **2012**, *11* (5), 436-443. DOI: 10.1038/nmat3271.

(63) Zhao, Z.; Angell, C. A. Apparent First-Order Liquid–Liquid Transition with Pre-transition Density Anomaly, in Water-Rich Ideal Solutions. *Angewandte Chemie International Edition* **2016**, *55* (7), 2474-2477. DOI: 10.1002/anie.201510717.

(64) Suzuki, Y.; Mishima, O. Experimentally proven liquid-liquid critical point of dilute glycerol-water solution at 150 K. *The Journal of Chemical Physics* **2014**, *141* (9). DOI: 10.1063/1.4894416.

(65) Popov, I.; Greenbaum, A.; Sokolov, A. P.; Feldman, Y. The puzzling first-order phase transition in water–glycerol mixtures. *Physical Chemistry Chemical Physics* **2015**, *17* (27), 18063-18071. DOI: 10.1039/C5CP02851E.

(66) Bachler, J.; Fuentes-Landete, V.; Jahn, D. A.; Wong, J.; Giovambattista, N.; Loerting, T. Glass polymorphism in glycerol–water mixtures: II. Experimental studies. *Physical Chemistry Chemical Physics* **2016**, *18* (16), 11058-11068, 10.1039/C5CP08069J. DOI: 10.1039/C5CP08069J.

(67) Ni, Y.; Skinner, J. L. Evidence for a liquid-liquid critical point in supercooled water within the E3B3 model and a possible interpretation of the kink in the homogeneous nucleation line. *The Journal of Chemical Physics* **2016**, *144* (21). DOI: 10.1063/1.4952991.

(68) Ito, K.; Moynihan, C. T.; Angell, C. A. Thermodynamic determination of fragility in liquids and a fragile-to-strong liquid transition in water. *Nature* **1999**, *398* (6727), 492-495. DOI: 10.1038/19042.

(69) Xu, L.; Kumar, P.; Buldyrev, S. V.; Chen, S.-H.; Poole, P. H.; Sciortino, F.; Stanley, H. E. Relation between the Widom line and the dynamic crossover in systems with a liquid–liquid phase transition. *Proceedings of the National Academy of Sciences* **2005**, *102* (46), 16558-16562. DOI: 10.1073/pnas.0507870102.

(70) Hestand, N. J.; Skinner, J. L. Perspective: Crossing the Widom line in no man's land: Experiments, simulations, and the location of the liquid-liquid critical point in supercooled water. *The Journal of Chemical Physics* **2018**, *149* (14). DOI: 10.1063/1.5046687.

(71) Miyata, K.; Kanno, H. Supercooling behavior of aqueous solutions of alcohols and saccharides. *Journal of Molecular Liquids* **2005**, *119* (1), 189-193. DOI: 10.1016/j.molliq.2004.10.026.

(72) Abascal, J. L. F.; Vega, C. Widom line and the liquid–liquid critical point for the TIP4P/2005 water model. *The Journal of Chemical Physics* **2010**, *133* (23). DOI: 10.1063/1.3506860.
